# Biocatalytic Thioketal Cleavage Enabled by Enzymatic Bromide Recycling by Vanadium-Dependent Haloperoxidases

**DOI:** 10.1021/acs.orglett.5c01137

**Published:** 2025-05-20

**Authors:** Manik Sharma, Yue Li, Kyle F. Biegasiewicz

**Affiliations:** † Department of Chemistry, 1371 Emory University, Atlanta, Georgia 30322, United States

## Abstract

Thioketals are an important class of molecules used for the preparation and protection of carbonyl compounds in chemical synthesis. Selective cleavage of thioketals requires the use of harsh conditions and reagents that limit the use of thioketals in chemoenzymatic synthesis. Herein, we describe a biocatalytic strategy for the cleavage of thioketals using enzymatic bromide recycling by vanadium-dependent haloperoxidase (VHPO) enzymes. This reaction design involves halogenation-mediated thioketal cleavage through repetitive enzyme-mediated formation of hypobromous acid with a catalytic quantity of bromide salt and hydrogen peroxide as the terminal oxidant. This protocol is demonstrated on a broad range of 1,3-dithiolanes in high yield and excellent chemoselectivity, performed on a gram scale, run with lysate and whole cells, and applied to the cleavage of 1,3-dithianes and 1,3-oxathiolanes.

## Graphical abstract

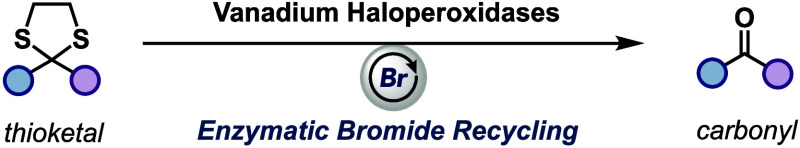

Thioketals are a class of sulfur-containing compounds that have broad application in the synthesis and protection of carbonyls in chemical synthesis.^2,3^ Despite their demonstrated utility in organic chemistry, deprotection of thioketals often involves the use of stoichiometric heavy metals,^4^ strong acids,^5^ alkylating agents, or stoichiometric oxidants that are either hazardous or produce stoichiometric quantities of byproducts.^2,3,6,7^ While photochemical methods for thioketal cleavage have also been developed using oxygen as the terminal oxidant,^8^ the reliance on the production of singlet oxygen limits their functional group tolerance. Efforts have also been made using catalytic quantities of metal-based catalysts with hydrogen peroxide (H_2_O_2_) as the terminal oxidant,^9^ but these methods require high catalyst loadings and operate under conditions that are largely bioincompatible, limiting their potential application in chemoenzymatic synthesis (Figure 1a).

**Figure 1:**
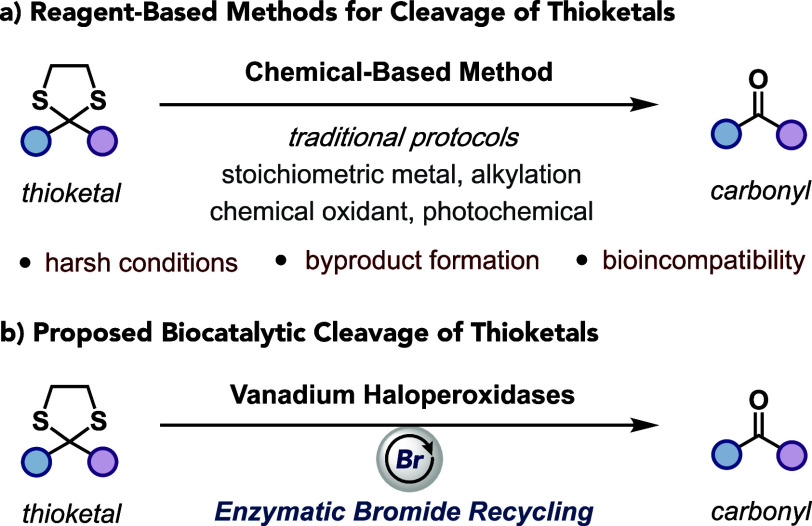
(a) Reagent-based methods for cleavage of thioketals. (b) Proposed biocatalytic deprotection of thioketals.

Enzymes are an attractive alternative to current methods for thioketal cleavage because of their catalytic efficiency and inherent sustainability parameters.^10^ Despite the promise of using enzymes for thioketal cleavage, a biocatalytic method for this transformation has remained elusive. We recently hypothesized that the vanadium-dependent haloperoxidase (VHPO) class of enzymes could address this challenge based on their recent emergence as a biocatalyst platform in synthetic organic chemistry.^11^ In nature, VHPOs are responsible for electrophilic halogenation of a broad range of organic substrates through catalytic oxidation of halides using H_2_O_2_ as the terminal oxidant.^12^ We recently realized the synthetic potential of VHPOs in performing halogenation-mediated processes using an enzymatic halide recycling (EHR) mechanism.^13,14^ EHR involves the repetitive oxidation of a catalytic quantity of halide to generate hypohalous acid as a halogenating agent to perform halogenation-mediated reactions. Herein, we report that VHPOs are a viable catalyst platform for thioketal cleavage using enzymatic bromide recycling (Figure 1b).

Our studies began by investigating the conversion of 2-methyl-2-phenyl-1,3-dithiolane (**1**) to give acetophenone (**2**).^2^ The chloroperoxidase from *Curvularia inaequalis* (*Ci*VCPO) was initially tested based on its documented utility in synthetic transformations.^12a,15^ When **1** is treated with *Ci*VCPO (0.0125 mol %), sodium orthovanadate (Na_3_VO_4_, 1 mM), potassium bromide (KBr, 1.0 equiv), and hydrogen peroxide (H_2_O_2_, 1.0 equiv) in 1,4-piperazinediethanesulfonic acid (PIPES) buffer (150 mM, pH 6.5) containing acetonitrile (MeCN) as a cosolvent (30% v/v), **2** is obtained in 84% yield after 1.5 h (Figure 2, entry 1). Other structurally diverse VHPOs were also explored, including bromoperoxidases from *Acaryochloris marina* (*Am*VBPO),^16^ *Corallina officinalis* (*Co*VBPO),^17^ and *Corallina pilulifera* (*Cp*VBPO) (Figure 2, entries 2–4, respectively).^18^ Notably, *Cp*VBPO demonstrated a superior performance, generating **2** in 90% yield. Control reactions validated the critical role of each reaction component, including the enzyme (*Cp*VBPO), Na_3_VO_4_, KBr, and H_2_O_2_ (Figure 2, entries 5–8, respectively). Gratifyingly, by simply increasing the stoichiometry of H_2_O_2_ to 3.0 equiv, the reaction could be run under EHR conditions with only 0.3 equiv of bromide, providing **2** in 98% yield (Figure 2, entry 9). When the reaction is performed with a H_2_O_2_ loading of >3.0 equiv, a slight decrease in yield is observed (Figure S1). Additionally, KBr loadings between 0.1 and 4.0 equiv are well tolerated, with loadings of >1.0 equiv providing no additional improvement in yield (Figure S2). The pH and buffer type played critical roles in reaction performance (Figure S3). The reaction performs in an excellent yield (98%) across a collection of polar protic and aprotic solvents (MeOH, EtOH, ^*i*^PrOH, and MeCN) (Figure S4), with MeCN being the optimal solvent for substrate solubility in a loading of 10–50% (v/v) (Figure S5).

**Figure 2:**
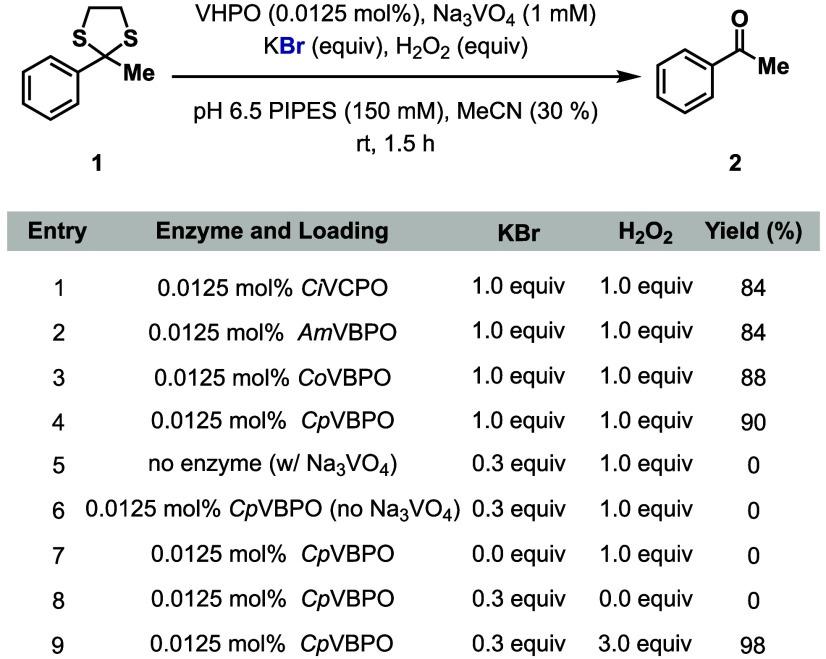
Optimization experiments for biocatalytic thioketal cleavage (**1a**). Reaction conditions: **1** (4.0 μmol, 0.8 mg), VHPO (0.0125 mol %), Na_3_VO_4_ (1 mM), KBr (0.3–1.0 equiv), H_2_O_2_ (1.0–3.0 equiv), PIPES buffer (150 mM, 300 μL, pH 6.5), MeCN (300 μL), 1 mL total reaction volume, 1.5 h, rt. Yields determined by HPLC based on a calibration curve. See the Supporting Information for details.

With the optimized conditions in hand, we investigated the substrate scope of VHPO-catalyzed thioketal cleavage. A broad range of substituents were tolerated at the *para* position of the starting aryl ring, including bromo, chloro, fluoro, methoxy, methyl, *tert*-butyl, nitro, phenyl, and a methyl ester (Figure 3, **3–11**, respectively), affording 86–92% yields with total turnover numbers (TTNs) of 6880–7360. Methyl and methoxy substituents were well tolerated at the *meta* position (90–91% yields and TTNs of 7200–7280 (Figure 3, **12** and **13**, respectively) and the *ortho* position (87–90% yields and TTNs of 6960–7200). When the aromatic was modified to a thiophene, the reaction proceeded with both methyl- and phenyl-ketone substituents in 89–90% yields with TTNs of 7120–7200 (Figure 3, **16** and **17**, respectively). When the aromatic is changed to more bulky rings, including naphthyl- and dihydrobenzofuran-substituted ketones, the reaction affords 90–91% yields with TTNs of 7200–7280 (Figure 3, **18** and **19**, respectively). The reaction is also effective on 1,3-dithiolanes derived from tetralone, propiophenone, and 4-phenylcyclohexanone (86–93% yields and TTNs of 6880–7440 (Figure 3, **20–22**, respectively). The reaction was performed on a thioacetal derived from benzaldehyde, affording a 92% yield and a TTN of 7360 (Figure 3, **23**). Gratifyingly, the protocol was also effective for cleavage of more functionalized thioacetals derived from vanillin, 4-dimethylaminobenzaldehyde, 3-pyridinecarboxyaldehyde, and 3-indolecarboxyaldehyde in 85–89% yields with TTNs of 6800–7120 (Figure 3, **24–27**, respectively). Finally, the acetal derived from acetophenone (**28**) was unreactive in this catalyst system.

**Figure 3:**
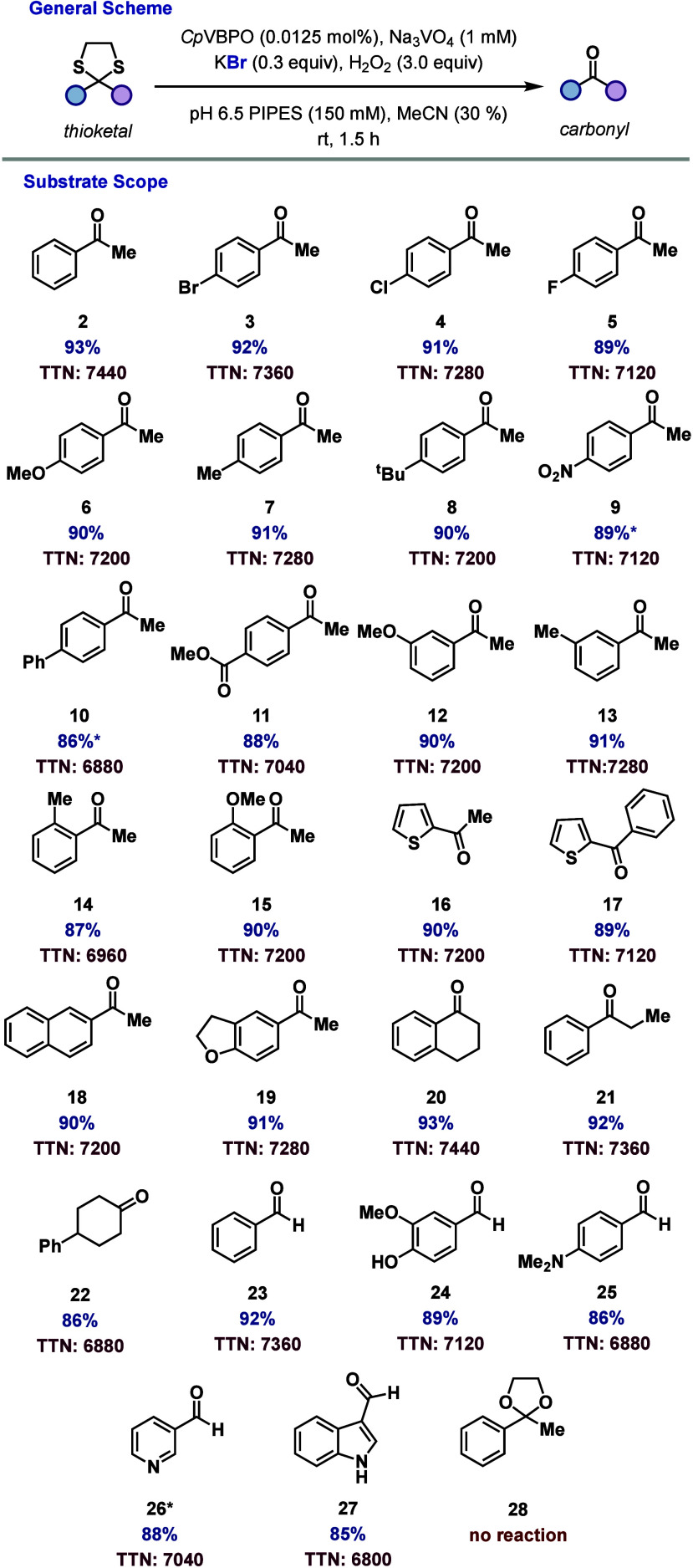
Substrate scope for VHPO-catalyzed thioketal cleavage. Reaction conditions: (1) substrate (0.8 mmol), *Cp*VBPO (0.0125 mol %), Na_3_VO_4_ (final concentration of 1 mM), KBr (0.3 equiv), H_2_O_2_ (3.0 equiv), PIPES buffer (150 mM, pH 6.5), MeCN (30%), 1.5 h, rt, *2.0 h run time. (2) Yields determined by isolation. TTNs were determined by dividing the quantity of the resulting product by the concentration of the enzyme used. See the Supporting Information for more details.

A proposed mechanism for VHPO-mediated thioketal cleavage is outlined in Figure 4. In analogy to previously proposed mechanisms,^12^ the vanadate cofactor of a VHPO is coordinated to a histidine residue (**I**). Exposure to H_2_O_2_ causes the displacement of two water molecules and the formation of peroxovanadium intermediate **II**. Subsequent bromide-mediated opening of **II** leads to vanadium-bound hypobromite (**III**), which upon displacement with water leads to the generation of hypobromous acid (HOBr). In analogy to the mechanism proposed by Tong,^9e^ the HOBr is responsible for direct bromination of the starting thioketal to give a brominated thioketal (**IV**) that undergoes hydrolysis to generate the desired carbonyl compound, a cyclic disulfide (**V**), and bromide that can be recycled for an ensuing oxidation event by the VHPO.

**Figure 4:**
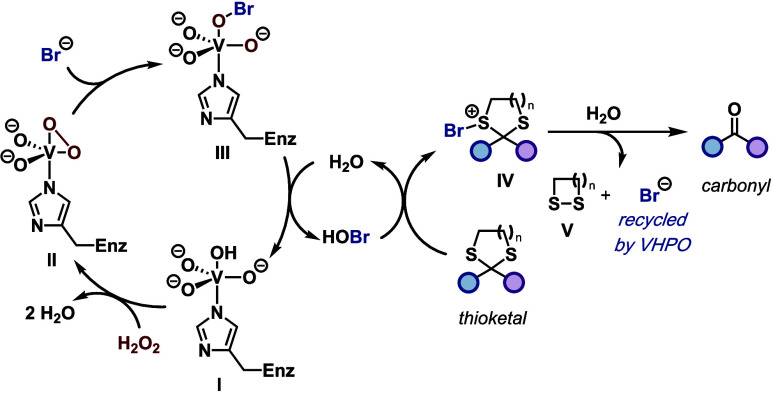
Proposed mechanism for VHPO-mediated thioketal cleavage.

We next evaluated the scalability and practical applicability of our VHPO-catalyzed thioketal cleavage protocol through several key experiments. The conversion of **1** into **2** was readily performed on a gram scale with no change in reaction performance (Figure 5a). Reactions were conducted using wet and dry lysate and whole cells, resulting in yields ranging from 89% to 90% (Figure 5b). The VHPO-mediated thioketal cleavage protocol was also readily extended to a 1,3-dithiane derived from acetophenone (**29**) to give **2** in 92% yield with a TTN of 7360 (Figure 5c). Finally, this protocol could be readily translated to 1,3-oxathiolane cleavage in the conversion of oxathiolane **30** into **2** in 93% yield with a TTN of 7440 (Figure 5d).

**Figure 5:**
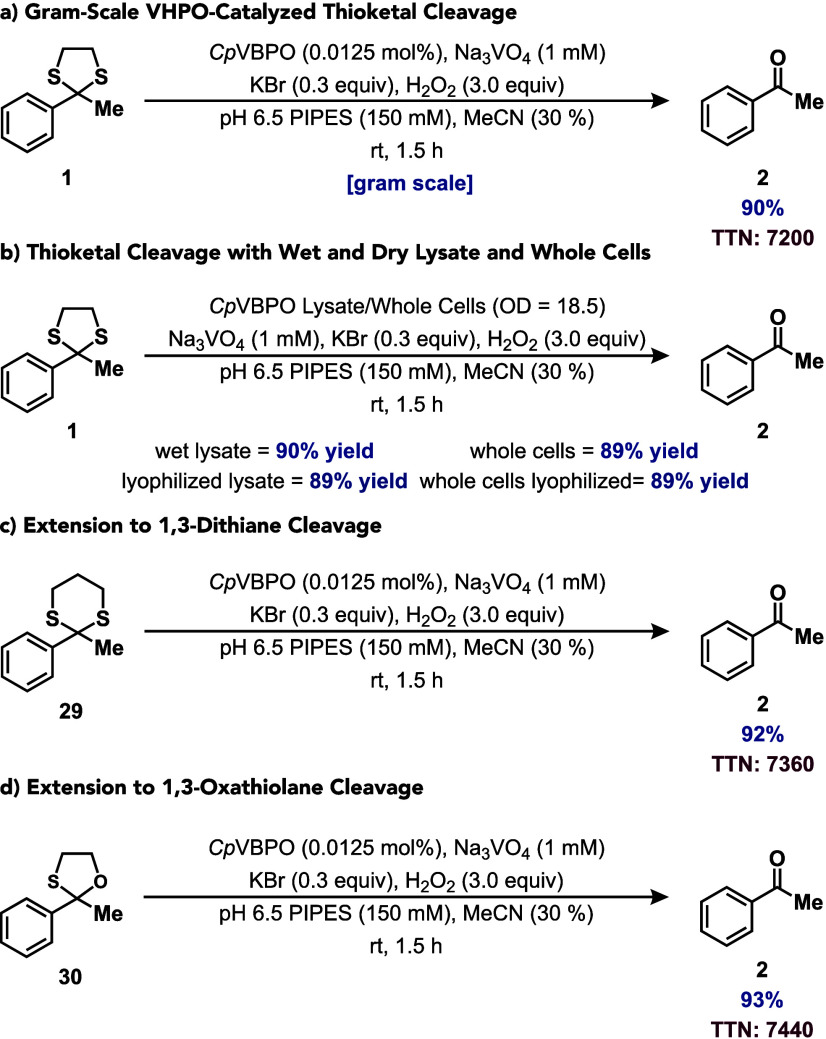
(a) Gram-scale VHPO-catalyzed thioketal cleavage. (b) Thioketal cleavage with wet and dry lysate and whole cells. (c) Extension to 1,3-dithiane cleavage. (d) Extension to 1,3-oxathiolane cleavage.

In conclusion, we discovered that VHPOs are viable biocatalysts for thioketal cleavage to afford the corresponding carbonyl compounds. The reaction is effective across a wide range of 1,3-dithiolanes, and the protocol can be extended to thioacetals and oxathiolanes. The reaction is scalable and can be run in wet and dry lysates and whole cells. This study not only provides a sustainable and biocompatible protocol for dithiane cleavage but also expands the application of VHPO-mediated enzymatic halide recycling in chemical synthesis.

## Supplementary Material



## Data Availability

The data underlying this study are available in the published article and its Supporting Information.

## Notes

Protein expression, experimental procedures, NMR spectra, NMR data, and Figures S1–S5 (PDF)
