# Supplementary material for: Biocatalytic Thioketal Cleavage Enabled by Enzymatic Bromide Recycling by Vanadium-Dependent Haloperoxidases
Source: Org Lett. 2025 May 20;27(22):5584–8. doi: 10.1021/acs.orglett.5c01137 (PMC12150324; doi:10.1021/acs.orglett.5c01137)

Supplementary Materials for

Biocatalytic Thioketal Cleavage Enabled by Enzymatic Bromide Recycling by Vanadium-Dependent Haloperoxidases

Manik Sharma[+‡], Yue Li[+‡], Kyle F. Biegasiewicz[+*]

[+] Department of Chemistry, Emory University, Atlanta, Georgia 30322, USA

[‡] Authors Contributed Equally

This PDF file includes:

Materials and Methods
Product Characterizations
Supplementary Text
Supplementary Figures S1-S5
Spectral Data
References

Table of Contents

General Information……………………………………………………………………… 3

Procedures for the Preparation of Lysate and Whole Cells Expressing *Cp*VBPO…… 4

Synthesis and Characterization of Thioketal Substrates……….……………….......… 5

General Procedures for VHPO-Catalyzed Thioketal Hydrolysis……………...…..... 19

Product Characterization for Carbonyl Compounds…………………….……….… 21

Additional Reaction Procedures……………………………………….…..….… 36

Optimization Data………………………………………………………… .……. 41

References……………………………………………………………………………… 44

Spectroscopic Data…………………………………………………………...... 46

General Experimental Information

General: Unless specified, all reagents and solvents used in this study were purchased from commercial suppliers and used as received (Combi-Blocks, Sigma-Aldrich, Oakwood Chemicals, Fischer Scientific, VWR). All nonaqueous reactions were performed using glassware that was flame-dried and capped with a rubber septum under nitrogen atmosphere using an inlet and outlet needle connected to a mineral oil bubbler. All aqueous reactions were conducted using glassware without flame-drying prior to experimental set up and without nitrogen atmosphere. For experiments requiring dried or degassed solvent, it was obtained from a solvent purification system from Pure Process Technology. Unless otherwise indicated, deionized water (H_2O) was used in any experiments where H_2O is included in the procedure.

Chromatography: Flash chromatography was performed on SiliaFlash® P60 (230-400 mesh, particle size 0.040-0.063 mm) using the listed solvent systems in each procedure. Thin-layer chromatography (TLC) was performed using Uniplate HLF 250 micron F254 precoated glass plates and preparative TLC was performed on Uniplate GF 1000 micron F254 precoated glass plates. For TLC analysis, a short-wave UV lamp and/or plate staining was used.

Spectroscopy and HRMS Anaylsis: ^{1}H- and ^{13}C-NMR were obtained on a Bruker AVIII or Bruker NEO (400 and 101 MHz, respectively). Chemical shifts are reported in ppm (δ) downfield from tetramethylsilane and are internally referenced to the internal deuterated solvent indicated. ^{1}H-NMR data is reported as follows: chemical shift [multiplicity, coupling constant (Hz), number of hydrogens]. Multiplicities are reported as follows: s (singlet), b (broad signal), d (doublet), dd (doublet of doublets), ddd (doublet of doublet of doublets), t (triplet), dt (doublet of triplets), tt (triplet of triplets), q (quartet), dq (doublet of quartets), p (pentet), m (multiplet). High-resolution mass spectra were obtained on a Thermo Finnigan LTQ-FTMS spectrometer using APCI with an orbitrap mass analyzer.

Analytical: Analytical high-performance liquid chromatography (HPLC) was carried out using a Shimadzu LCMS-2020 System with a Kromasil EternityXT-2.5-C18 column (Dimensions: 4.6x50mm, Batch/Serial: 0000016627/A, Part No. XH2CLA05).

Protein Expression and Purification: All protein expression and purification were performed using previously reported methods.[1]

Procedures for the Preparation of Lysate and Whole cells Expressing *Cp*VBPO

Procedure for the Preparation of Wet Lysate Expressing *Cp*VBPO: *E. coli* cells expressing *Cp*VBPO were pelleted using centrifugation at 3.5 krpm for 20 minutes at 10 °C in a Sorvall ST Plus centrifuge, resuspended in buffer (25 mM pH 6.5 PIPES/ H_2SO_4 buffer, 2.5 mM $Ca(NO_3)_2$, 25 mM Na_2SO_4) adjusted to an of OD_{600} = 18.5. Cells were lysed using Qsonica Q500 sonicator with a ½ inch probe on ice in 15 s pulses at 32% amplitude, 30 W in 10 bursts with 55 s between bursts. The lysed cell solution was clarified using centrifugation at 13.0 krpm for 20 minutes at 10 °C to remove cell debris and subsequently transferred into a separate 50 mL conical tube. The clarified cell lysate solution was then aliquoted into 2 mL microcentrifuge tubes, which were then flash frozen using liquid nitrogen and stored at -80 °C until further use.

Procedure for the Preparation of Lyophilized Cell Lysate Expressing *Cp*VBPO: *E. coli* cells expressing *Cp*VBPO were pelleted using centrifugation at 3.5 krpm for 20 minutes at 10 °C in a Sorvall ST Plus centrifuge, resuspended in buffer (25 mM pH 6.5 PIPES/H_2SO_4 buffer, 2.5 mM $Ca(NO_3)_2$, 25 mM Na_2SO_4) adjusted to the OD_{600} = 18.5. Cells were lysed using Qsonica Q500 sonicator with a ½ inch probe on ice in 15 s pulses at 32% amplitude, 30 W in 10 bursts with 55 s between bursts. The lysed cell solution was clarified using centrifugation at 13.0 krpm for 20 minutes at 10 °C to remove cell debris and subsequently transferred into a separate 50 mL conical tube. The clarified cell lysate solution was then flash frozen and lyophilized to yield dried cell lysate that was stored in 4 °C until further use.

Procedure for the Preparation of Whole Cells Expressing *Cp*VBPO: *E. coli* cells expressing *Cp*VBPO were pelleted using centrifugation at 3.5 krpm for 20 minutes at 10 °C in a Sorvall ST Plus centrifuge, resuspended in buffer (25 mM pH 6.5 PIPES/ H_2SO_4 buffer, 2.5 mM $Ca(NO_3)_2$, 25 mM Na_2SO_4) adjusted to the OD_{600} = 18.5. The whole cell solution was then aliquoted into 2 mL microcentrifuge tubes and were then flash frozen using liquid nitrogen and stored at -80 °C until further use.

Procedure for the Preparation of Lyophilized Whole Cells Expressing *Cp*VBPO: *E. coli* cells expressing *Cp*VBPO were pelleted using centrifugation at 3.5 krpm for 20 minutes at 10 °C in a Sorvall ST Plus centrifuge, resuspended in buffer (25 mM pH 6.5 PIPES/ H_2SO_4 buffer, 2.5 mM $Ca(NO_3)_2$, 25 mM Na_2SO_4) adjusted to the OD_{600} = 18.5. The whole cell solution was then flash frozen and lyophilized to yield dried cell lysate and was stored at 4 °C until further use.

Synthesis and Characterization of Thioketal Substrates

General Procedure for the Preparation of Thioketal/Thioacetal Substrates (General Procedure A):

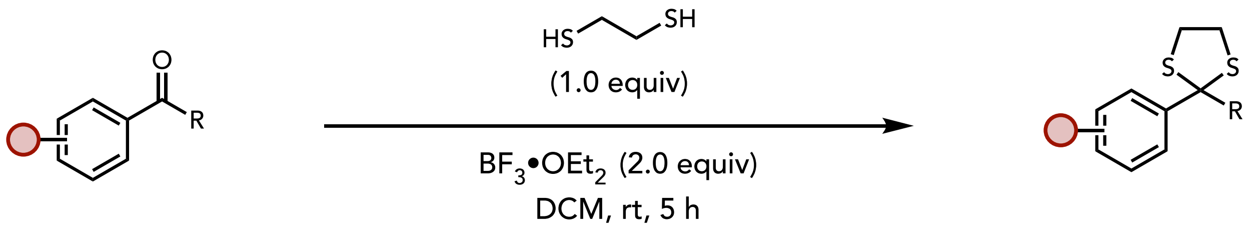

The following was adapted according to a literature procedure.[2] A solution of the corresponding ketones/aldehydes (8 mmol, 1.0 equiv) in CH_2Cl_2 (40 mL, 0.2 M) was prepared. To this solution 1,2-ethanedithiol (0.67 mL, 8 mmol, 1.0 equiv) and BF_3•OEt_2 (1.97 mL, 16 mmol, 2.0 equiv) were added at room temperature. The solution was allowed to stir at room temperature for 5 h. After the indicated time, the reaction mixture was quenched using saturated aqueous $NaHCO_3$ (100 mL) and extracted with CH_2Cl_2 (3 x 50 mL). The combined organic extracts were washed with 50 mL of brine, then dried over anhydrous sodium sulfate, and concentrated under reduced pressure. The resulting crude residue was purified by flash column chromatography, yielding the desired substrate.

General Procedure for the Preparation of Thioacetal Substrates (General Procedure B):

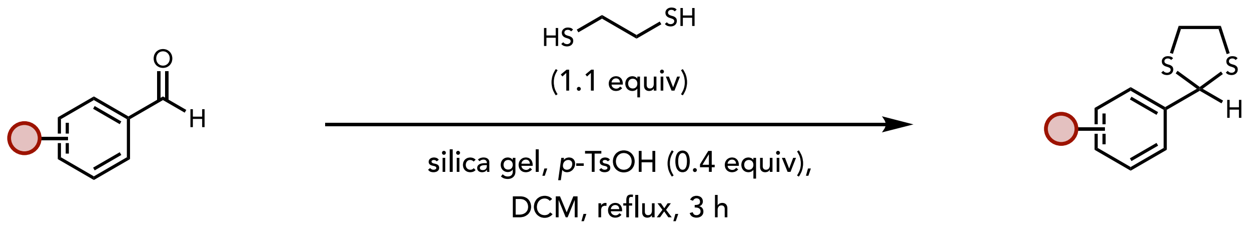

The following was adapted according to a literature procedure.[3] A 100 mL round-bottom flask equipped with a condenser was charged with silica gel (8.0 g), CH_2Cl_2 (40 mL, 0.13 M), an aldehyde (5.0 mmol), 1,2-ethanedithiol (0.46 mL, 5.50 mmol, 1.5 equiv), and *p*-toluenesulfonic acid monohydrate (38.0 mg, 0.2 mmol, 0.4 equiv). The resulting heterogeneous mixture was refluxed using sand bath for 3 h. After the indicated time reaction mixture was filtered through a sintered- glass funnel. The solid residue was washed with CH_2Cl_2 (100 mL) The solvent was evaporated under reduced pressure and the resulting crude residue was purified by flash column chromatography, yielding the desired substrate.

Characterization of Dithiolanes:

2-Methyl-2-phenyl-1,3-dithiolane (1)[4]

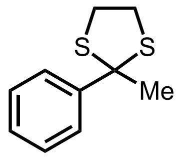

Synthesized from commercially available acetophenone following General Procedure A and was obtained as a colorless liquid.

Yield: 75% (1.57 g)

Purification: Eluted in 5% EtOAc in Hexanes

^{1}H NMR (400 MHz, $CDCl_3$) δ 7.78 – 7.73 (m, 2H), 7.32 (ddd, J = 7.8, 6.9, 1.3 Hz, 2H), 7.26 – 7.20 (m, 1H), 3.51 – 3.35 (m, 4H), 2.16 (s, 3H).

^{13}C NMR (101 MHz, $CDCl_3$) δ 146.0, 128.2, 127.2, 126.9, 68.7, 40.5, 34.0.

2-(4-Bromophenyl)-2-methyl-1,3-dithiolane (SM-2)[5]

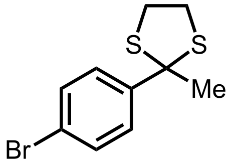

Synthesized from commercially available 1-(4-bromophenyl)ethan-1-one following General Procedure A and was obtained as a colorless liquid.

Yield: 80% (1.76 g)

Purification: Eluted in 5% EtOAc in Hexanes

^{1}H NMR (400 MHz, $CDCl_3$) δ 7.67 – 7.60 (m, 2H), 7.46 – 7.38 (m, 2H), 3.50 – 3.31 (m, 4H), 2.12 (s, 3H).

^{13}C NMR (101 MHz, $CDCl_3$) δ 145.4, 131.1, 128.8, 121.2, 68.1, 40.5, 33.6.

2-(4-Clorophenyl)-2-methyl-1,3-dithiolane (SM-3)[4]

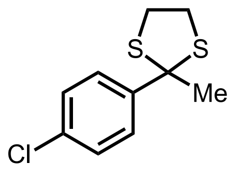

Synthesized from commercially available 1-(4-chlorophenyl)ethan-1-one following General Procedure A and was obtained as a colorless liquid.

Yield: 82% (1.51 g)

Purification: Eluted in 5% EtOAc in Hexanes

^{1}H NMR (400 MHz, $CDCl_3$) δ 7.73 – 7.65 (m, 2H), 7.30 – 7.24 (m, 2H), 3.52 – 3.31 (m, 4H), 2.13 (s, 3H).

^{13}C NMR (101 MHz, $CDCl_3$) δ 144.9, 133.0, 128.5, 128.2, 68.1, 40.6, 33.7.

2-(4-Fluorophenyl)-2-methyl-1,3-dithiolane (SM-4)[2]

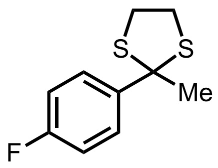

Synthesized from commercially available 1-(4-fluorophenyl)ethan-1-one following General Procedure A and was obtained as a colorless liquid.

Yield: 78% (1.71 g)

Purification: Eluted in 5% EtOAc in Hexanes

^{1}H NMR (400 MHz, $CDCl_3$) δ 7.78 – 7.69 (m, 2H), 7.02 – 6.94 (m, 2H), 3.52 – 3.34 (m, 4H), 2.14 (s, 3H).

^{13}C NMR (101 MHz, $CDCl_3$) δ 161.9 (d, J = 247.4 Hz), 141.8 (d, J = 3.2 Hz), 128.8 (d, J = 8.1 Hz), 114.8 (d, J = 21.5 Hz), 68.1, 40.53, 34.0.

2-(4-Methoxyphenyl)-2-methyl-1,3-dithiolane (SM-5)[4]

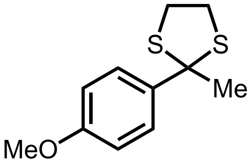

Synthesized from commercially available 1-(4-methoxyphenyl)ethan-1-one following General Procedure A and was obtained as a colorless liquid.

Yield: 73% (1.32 g)

Purification: Eluted in 5% EtOAc in Hexanes

^{1}H NMR (400 MHz, $CDCl_3$) δ 7.68 (d, J = 8.9 Hz, 2H), 6.86 – 6.81 (m, 2H), 3.80 (s, 3H), 3.50 – 3.37 (m, 4H), 2.15 (s, 3H).

^{13}C NMR (101 MHz, $CDCl_3$) δ 158.7, 137.8, 128.2, 113.3, 68.3, 55.4, 40.5, 34.0.

2-Methyl-2-(p-tolyl)-1,3-dithiolane (SM-6)[4]

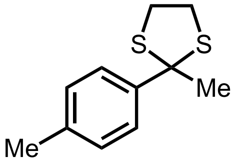

Synthesized from commercially available 1-(p-tolyl)ethan-1-one following General Procedure A and was obtained as a white solid.

Yield: 76% (1.28 g)

Purification: Eluted in 5% EtOAc in Hexanes

^{1}H NMR (400 MHz, $CDCl_3$) δ 7.64 (d, J = 8.3 Hz, 2H), 7.13 (d, J = 8.2 Hz, 2H), 3.51 – 3.35 (m, 4H), 2.34 (s, 3H), 2.15 (s, 3H).

^{13}C NMR (101 MHz, $CDCl_3$) δ 143.0, 137.0, 128.8, 126.8, 68.5, 40.5, 34.0, 21.1.

2-(4-(tert-Butyl)phenyl)-2-methyl-1,3-dithiolane (SM-7)[6]

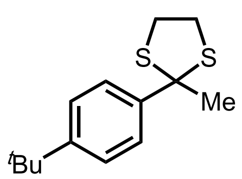

Synthesized from commercially available 1-(4-(tert-butyl)phenyl)ethan-1-one following General Procedure A and was obtained as a white solid.

Yield: 82% (1.66 g)

Purification: Eluted in 5% EtOAc in Hexanes

^{1}H NMR (400 MHz, $CDCl_3$) δ 7.67 (d, J = 8.6 Hz, 2H), 7.33 (d, J = 8.6 Hz, 2H), 3.51 – 3.38 (m, 4H), 2.16 (s, 3H), 1.31 (s, 9H).

^{13}C NMR (101 MHz, $CDCl_3$) δ 150.1, 142.8, 126.6, 125.1, 68.4, 40.5, 34.5, 34.1, 31.41.

2-Methyl-2-(4-nitrophenyl)-1,3-dithiolane (SM-8)[4]

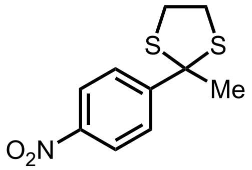

Synthesized from commercially available 1-(4-nitrophenyl)ethan-1-one following General Procedure A and was obtained as a colorless liquid.

Yield: 75% (1.45 g)

Purification: Eluted in 10% EtOAc in Hexanes

^{1}H NMR (400 MHz, $CDCl_3$) δ 8.19 – 8.08 (m, 2H), 7.96 – 7.87 (m, 2H), 3.54 – 3.31 (m, 4H), 2.15 (s, 3H).

^{13}C NMR (101 MHz, $CDCl_3$) δ 153.9, 146.9, 128.0, 123.3, 67.8, 40.8, 33.3.

2-([1,1'-Biphenyl]-4-yl)-2-methyl-1,3-dithiolane (SM-9)[2]

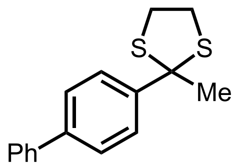

Synthesized from commercially available 1-([1,1'-biphenyl]-4-yl)ethan-1-one acid following General Procedure A and was obtained as a colorless liquid.

Yield: 80% (1.74 g)

Purification: Eluted in 5% EtOAc in Hexanes

^{1}H NMR (400 MHz, $CDCl_3$) δ 7.85 – 7.80 (m, 2H), 7.61 – 7.52 (m, 4H), 7.47 – 7.41 (m, 2H), 7.38 – 7.32 (m, 1H), 3.54 – 3.39 (m, 4H), 2.20 (s, 3H).

^{13}C NMR (101 MHz, $CDCl_3$) δ 145.1, 140.7, 140.1, 128.9, 127.5, 127.2, 126.9, 68.5, 40.6, 33.9.

Methyl 4-(2-methyl-1,3-dithiolan-2-yl)benzoate (SM-10)

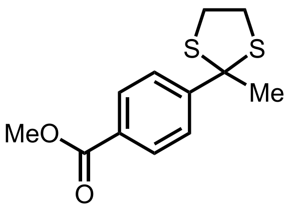

Synthesized from commercially available methyl 4-acetylbenzoate following General Procedure A and was obtained as a colorless liquid.

Yield: 71% (2.03 g)

Purification: Eluted in 10% EtOAc in Hexanes

^{1}H NMR (400 MHz, $CDCl_3$) δ 8.03 – 7.93 (m, 2H), 7.86 – 7.76 (m, 2H), 3.91 (s, 3H), 3.52 – 3.31 (m, 4H), 2.15 (s, 3H).

^{13}C NMR (101 MHz, $CDCl_3$) δ 166.9, 151.5, 129.5, 129.0, 127.0, 68.4, 52.3, 40.6, 33.6.

HRMS (APCI) m/z: calculated for $C_{12}H_{15}O_2S_2$ $[M+H]^+$: 255.0508. Found: 255.0508.

2-(3-Methoxyphenyl)-2-methyl-1,3-dithiolane (SM-11)[7]

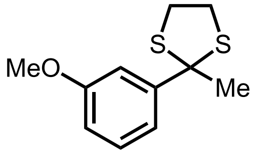

Synthesized from commercially available 1-(3-methoxyphenyl)ethan-1-one following General Procedure A and was obtained as a colorless liquid.

Yield: 77% (1.39 g)

Purification: Eluted in 5% EtOAc in Hexanes

^{1}H NMR (400 MHz, $CDCl_3$) δ 7.36 – 7.31 (m, 2H), 7.26 – 7.21 (m, 1H), 6.78 (ddd, J = 8.1, 2.5, 1.1 Hz, 1H), 3.82 (s, 3H), 3.50 – 3.33 (m, 4H), 2.14 (s, 3H).

^{13}C NMR (101 MHz, $CDCl_3$) δ 159.3, 147.8, 129.2, 119.3, 113.2, 112.2, 68.7, 55.4, 40.4, 33.9.

2-Methyl-2-(m-tolyl)-1,3-dithiolane (SM-12)[4]

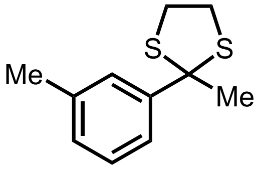

Synthesized from commercially available 1-(m-tolyl)ethan-1-one following General Procedure A and was obtained as a colorless liquid.

Yield: 80% (1.35 g)

Purification: Eluted in 5% EtOAc in Hexanes

^{1}H NMR (400 MHz, $CDCl_3$) δ 7.59 – 7.53 (m, 2H), 7.21 (t, J = 7.6 Hz, 1H), 7.06 (ddt, J = 7.5, 1.8, 0.9 Hz, 1H), 3.51 – 3.36 (m, 4H), 2.37 (s, 3H), 2.15 (s, 3H).

^{13}C NMR (101 MHz, $CDCl_3$) δ 145.9, 137.8, 128.0, 128.0, 127.6, 123.9, 68.7, 40.4, 34.2, 21.7.

2-Methyl-2-(o-tolyl)-1,3-dithiolane (SM-13)[4]

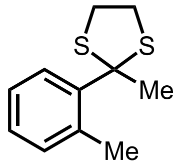

Synthesized from commercially available 1-(o-tolyl)ethan-1-one following General Procedure A and was obtained as a colorless liquid.

Yield: 78% (1.31 g)

Purification: Eluted in 5% EtOAc in Hexanes

^{1}H NMR (400 MHz, $CDCl_3$) δ 7.93 (dd, *J* = 6.9, 1.8 Hz, 1H), 7.21 – 7.12 (m, 3H), 3.49 – 3.29 (m, 4H), 2.67 (s, 3H), 2.19 (s, 3H).

^{13}C NMR (101 MHz, $CDCl_3$) δ 142.7, 136.4, 133.0, 127.5, 126.9, 125.7, 69.6, 39.9, 33.4, 22.8.

2-(2-Methoxyphenyl)-2-methyl-1,3-dithiolane (SM-14)

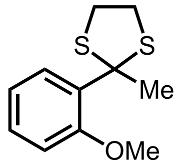

Synthesized from commercially available 1-(2-methoxyphenyl)ethan-1-one following General Procedure A and was obtained as a colorless liquid.

Yield: 75% (1.36 g)

Purification: Eluted in 5% EtOAc in Hexanes

^{1}H NMR (400 MHz, $CDCl_3$) δ 7.86 (dd, *J* = 7.6, 1.7 Hz, 1H), 7.28 – 7.22 (m, 1H), 6.91 (td, *J* = 7.9, 1.1 Hz, 2H), 3.92 (s, 3H), 3.43 – 3.32 (m, 2H), 3.29 – 3.17 (m, 2H), 2.15 (s, 3H).

^{13}C NMR (101 MHz, $CDCl_3$) δ 156.9, 134.5, 128.7, 126.6, 120.3, 112.1, 68.0, 55.6, 39.5, 32.6.

HRMS (APCI) m/z: calculated for $C_{11}H_{15}OS_2$ $[M+H]^+$: 227.0559. Found: 227.0562.

2-Methyl-2-(thiophen-2-yl)-1,3-dithiolane (SM-15)[8]

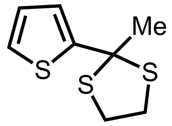

Synthesized from commercially available 1-(thiophen-2-yl)ethan-1-one following General Procedure A and was obtained as a colorless liquid.

Yield: 69% (1.62 g)

Purification: Eluted in 5% EtOAc in Hexanes

^{1}H NMR (400 MHz, $CDCl_3$) δ 7.19 (dd, *J* = 5.3, 1.3 Hz, 1H), 7.11 (dd, *J* = 3.7, 1.3 Hz, 1H), 6.89 (dd, *J* = 5.2, 3.6 Hz, 1H), 3.49 (s, 4H), 2.24 (s, 3H).

^{13}C NMR (101 MHz, $CDCl_3$) δ 153.6, 126.8, 125.3, 124.9, 64.4, 41.0, 34.0.

2-Phenyl-2-(thiophen-2-yl)-1,3-dithiolane (SM-16)

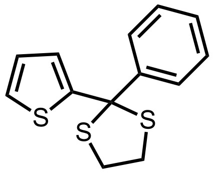

Synthesized from commercially available phenyl(thiophen-2-yl)methanone following General Procedure A and was obtained as a white solid.

Yield: 78% (1.65 g)

Purification: Eluted in 5% EtOAc in Hexanes

^{1}H NMR (400 MHz, $CDCl_3$) δ 7.71 – 7.66 (m, 2H), 7.34 – 7.23 (m, 4H), 6.95 (dd, *J* = 3.6, 1.3 Hz, 1H), 6.89 (dd, *J* = 5.1, 3.6 Hz, 1H), 3.51 (d, *J* = 0.9 Hz, 4H).

^{13}C NMR (101 MHz, $CDCl_3$) δ 152.0, 143.5, 128.1, 128.1, 128.0, 127.9, 126.8, 126.1, 72.8, 40.6.

HRMS (APCI) m/z: calculated for $C_{13}H_{13}S_3$ $[M+H]^+$: 265.0174. Found: 265.0176.

2-Methyl-2-(naphthalen-2-yl)-1,3-dithiolane (SM-17)[9]

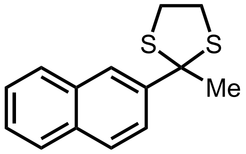

Synthesized from commercially available 1-(naphthalen-2-yl)ethan-1-one following General Procedure A and was obtained as a white solid.

Yield: 75% (1.48 g)

Purification: Eluted in 5% EtOAc in Hexanes

^{1}H NMR (400 MHz, $CDCl_3$) δ 8.18 (d, *J* = 2.0 Hz, 1H), 7.89 – 7.78 (m, 4H), 7.52 – 7.44 (m, 2H), 3.56 – 3.38 (m, 4H), 2.27 (s, 3H).

^{13}C NMR (101 MHz, $CDCl_3$) δ 143.1, 132.8, 132.5, 128.4, 128.0, 127.5, 126.3, 126.2, 124.8, 68.8, 40.6, 33.5.

5-(2-Methyl-1,3-dithiolan-2-yl)-2,3-dihydrobenzofuran (SM-18)

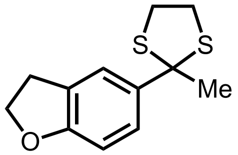

Synthesized from commercially available 1-(2,3-dihydrobenzofuran-5-yl)ethan-1-one following General Procedure A and was obtained as a white solid.

Yield: 82% (1.56 g)

Purification: Eluted in 10% EtOAc in Hexanes

^{1}H NMR (400 MHz, $CDCl_3$) δ 7.62 (dt, *J* = 2.2, 1.1 Hz, 1H), 7.49 (dt, *J* = 8.4, 1.4 Hz, 1H), 6.69 (d, *J* = 8.4 Hz, 1H), 4.57 (t, *J* = 8.7 Hz, 2H), 3.51 – 3.38 (m, 4H), 3.20 (t, *J* = 8.6 Hz, 2H), 2.14 (s, 3H).

^{13}C NMR (101 MHz, $CDCl_3$) δ 159.3, 137.7, 127.0, 126.8, 124.0, 108.4, 71.7, 68.6, 40.6, 34.4, 29.8.

HRMS (APCI) m/z: calculated for $C_{12}H_{15}OS_2$ $[M+H]^+$: 239.0559. Found: 239.0558.

3,4-Dihydro-2H-spiro[naphthalene-1,2'-[1,3]dithiolane] (SM-19)[10]

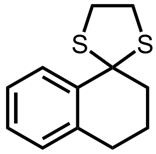

Synthesized from commercially available 3,4-Dihydronaphthalen-1(2H)-one following General Procedure A and was obtained as a white solid.

Yield: 75% (1.30 g)

Purification: Eluted in 5% EtOAc in Hexanes

^{1}H NMR (400 MHz, $CDCl_3$) δ 7.95 (dd, J = 8.0, 1.5 Hz, 1H), 7.21 – 7.16 (m, 1H), 7.12 (td, J = 7.4, 1.5 Hz, 1H), 7.00 (dd, J = 7.6, 1.5 Hz, 1H), 3.63 – 3.43 (m, 4H), 2.81 (t, J = 6.4 Hz, 2H), 2.46 – 2.34 (m, 2H), 2.07 – 1.96 (m, 2H).

^{13}C NMR (101 MHz, $CDCl_3$) δ 139.1, 137.4, 131.0, 128.8, 127.3, 126.2, 68.8, 43.9, 41.0, 29.6, 22.9.

2-Ethyl-2-phenyl-1,3-dithiolane (SM-20)

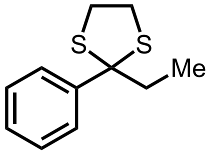

Synthesized from commercially available propiophenone following General Procedure A and was obtained as a colorless liquid.

Yield: 76% (1.28 g)

Purification: Eluted in 5% EtOAc in Hexanes

^{1}H NMR (400 MHz, $CDCl_3$) δ 7.74 – 7.64 (m, 2H), 7.37 – 7.27 (m, 2H), 7.26 – 7.18 (m, 1H), 3.42 – 3.22 (m, 4H), 2.39 (q, J = 7.3 Hz, 2H), 0.91 (t, J = 7.3 Hz, 3H).

^{13}C NMR (101 MHz, $CDCl_3$) δ 144.8, 128.0, 127.3, 127.0, 75.2, 39.3, 39.2, 12.2.

HRMS (APCI) m/z: calculated for $C_{11}H_{15}S_2$ $[M+H]^+$: 211.0610. Found: 211.0612

8-Phenyl-1,4-dithiaspiro[4.5]decane (SM-21)

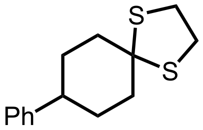

Synthesized from commercially available 4-phenylcyclohexan-1-one following General Procedure A and was obtained as a white solid.

Yield: 75% (1.50 g)

Purification: Eluted in 5% EtOAc in Hexanes

^{1}H NMR (400 MHz, $CDCl_3$) δ 7.35 – 7.28 (m, 2H), 7.26 – 7.18 (m, 3H), 3.38 – 3.29 (m, 4H), 2.53 (tt, J = 12.2, 3.7 Hz, 1H), 2.33 – 2.24 (m, 2H), 2.10 (td, J = 13.2, 3.7 Hz, 2H), 1.99 – 1.90 (m, 2H), 1.87 – 1.74 (m, 2H).

^{13}C NMR (101 MHz, $CDCl_3$) δ 146.5, 128.5, 126.9, 126.3, 68.2, 43.1, 43.0, 39.0, 38.2, 33.8.

HRMS (APCI) m/z: calculated for $C_{14}H_{19}S_2$ $[M+H]^+$: 251.0923. Found: 251.0925.

2-Phenyl-1,3-dithiolane (SM-22)[4]

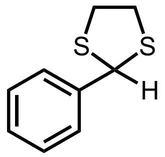

Synthesized from commercially available Benzaldehyde following General Procedure A and was obtained as a colorless liquid.

Yield: 76% (1.11 g)

Purification: Eluted in 5% EtOAc in Hexanes

^{1}H NMR (400 MHz, $CDCl_3$) δ 7.56 – 7.51 (m, 2H), 7.35 – 7.24 (m, 3H), 5.65 (s, 1H), 3.55 – 3.32 (m, 4H).

^{13}C NMR (101 MHz, $CDCl_3$) δ 140.4, 128.6, 128.2, 128.1, 56.4, 40.4.

4-(1,3-Dithiolan-2-yl)-2-methoxyphenol (SM-23)[11]

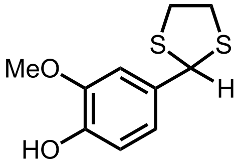

Synthesized from commercially available 4-hydroxy-3-methoxybenzaldehyde following General Procedure B and was obtained as a white solid.

Yield: 85% (0.97 g)

Purification: Eluted in 20% EtOAc in Hexanes

^{1}H NMR (400 MHz, $CDCl_3$) δ 7.10 (d, *J* = 2.1 Hz, 1H), 7.00 (dd, *J* = 8.2, 2.1 Hz, 1H), 6.83 (d, *J* = 8.2 Hz, 1H), 5.63 (d, *J* = 1.1 Hz, 2H), 3.91 (s, 3H), 3.55 – 3.46 (m, 2H), 3.39 – 3.30 (m, 2H).

^{13}C NMR (101 MHz, $CDCl_3$) δ 146.6, 145.7, 131.4, 121.2, 114.1, 110.4, 56.9, 56.1, 40.30.

4-(1,3-Dithiolan-2-yl)-N,N-dimethylaniline (SM-24)[11]

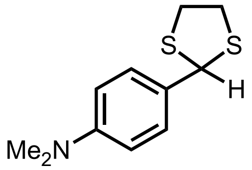

Synthesized from commercially available 4-(dimethylamino)benzaldehyde following General Procedure A and was obtained as a yellow solid.

Yield: 85% (1.53 g)

Purification: Eluted in 10% EtOAc in Hexanes

^{1}H NMR (400 MHz, $CDCl_3$) δ 7.47 – 7.36 (m, 2H), 6.69 – 6.63 (m, 2H), 5.65 (s, 1H), 3.55 – 3.46 (m, 2H), 3.38 – 3.29 (m, 2H), 2.94 (s, 6H).

^{13}C NMR (101 MHz, $CDCl_3$) δ 150.6, 128.9, 126.7, 112.4, 56.7, 40.7, 40.30.

3-(1,3-Dithiolan-2-yl)pyridine (SM-25)[12]

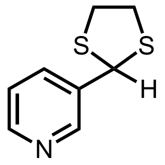

Synthesized from commercially available nicotinaldehyde following General Procedure A with the reaction run for 18 hours at reflux using THF as reaction solvent and was obtained as a colorless liquid.

Yield: 70% (1.02 g)

Purification: Eluted in 50% EtOAc in Hexanes

^{1}H NMR (400 MHz, $CDCl_3$) δ 8.66 (d, J = 2.3 Hz, 1H), 8.48 (dd, J = 4.8, 1.6 Hz, 1H), 7.86 (dt, J = 7.9, 2.1 Hz, 1H), 7.27 – 7.20 (m, 1H), 5.59 (s, 1H), 3.53 – 3.44 (m, 2H), 3.40 – 3.32 (m, 2H).

^{13}C NMR (101 MHz, $CDCl_3$) δ 149.4, 149.3, 136.7, 135.7, 123.5, 53.4, 40.5.

3-(1,3-Dithiolan-2-yl)-1H-indole (SM-26)[13]

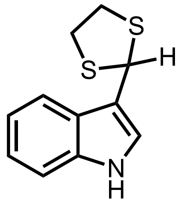

Synthesized from commercially available 1H-indole-3-carbaldehyde following General Procedure B and was obtained as a colorless liquid.

Yield: 71% (0.79 g)

Purification: Eluted in 20% EtOAc in Hexanes

^{1}H NMR (400 MHz, $CDCl_3$) δ 8.01 (s, 1H), 7.83 (ddt, J = 7.8, 1.5, 0.8 Hz, 1H), 7.38 – 7.29 (m, 2H), 7.19 (dddd, J = 22.5, 8.1, 7.1, 1.2 Hz, 2H), 6.06 (d, J = 0.8 Hz, 1H), 3.54 – 3.45 (m, 2H), 3.42 – 3.34 (m, 2H).

^{13}C NMR (101 MHz, $CDCl_3$) δ 137.0, 126.0, 123.3, 122.7, 119.9, 119.9, 115.2, 111.5, 49.03, 39.6.

General Analytical Procedure for the Biocatalytic Hydrolysis of Thioketals with *Cp*VBPO (General Procedure C):

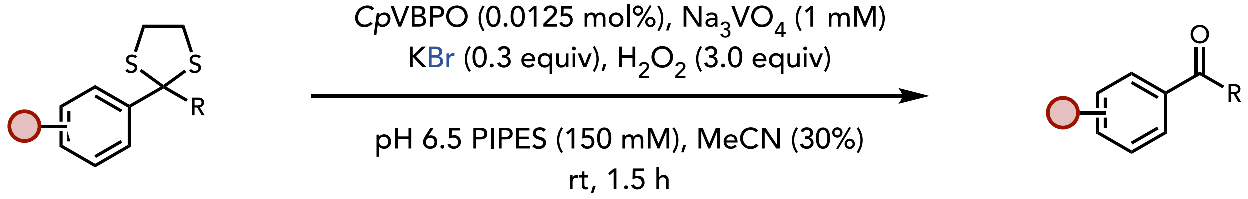

An enzyme aliquot of the VHPO from *Corallina pilulifera* (*Cp*VBPO, 10 µM, 50 µL) was removed from a -80 °C freezer and allowed to warm to room temperature over 5 min. After thawing, a 250 mM solution of aqueous Na_3VO_4 (4 µL) was added to the enzyme aliquot, and the resulting mixture was centrifuged for 10 seconds using a Chemglass Life Sciences MLX-108-CLS mini centrifuge and then placed at room temperature until further use. To a 1-dram vial was then added H_2O purified by an Elga purification system (335.2 µL), 500 mM pH 6.5 PIPES buffer (300 µL), and 176 mM aqueous KBr (6.82 µL, 0.3 equiv) followed by addition of 200 uL MeCN. A 40 mM solution of the dithiolane substrate in MeCN (100 µL, 1.0 equiv, 0.004 mmol substrate) was then added. The aliquot containing the *Cp*VBPO (0.0125 mol%, 1 µM in reaction) and Na_3VO_4 (0.25 equiv) was then added to the reaction mixture followed by a 10% stock of H_2O_2 (3.95 µL, 3.0 equiv). The vial was then capped and placed on a shaker at room temperature for 1.5 hr. After this time, the reaction mixture was diluted with MeCN (650 µL), transferred to an Eppendorf tube, and centrifuged in a Benchmark MC-24™ Touch Centrifuge at 12,500 rpm for 5 min. After centrifugation, 650 µL of the top layer of the reaction mixture was transferred to an LCMS vial, which was then placed on an LCMS for analysis.

**100 µL of 8 mg/mL solution of 1,3,5-tribromobenzene was added as an internal standard for yield confirmation, where applicable.*

General Preparative Procedure for the Biocatalytic Hydrolysis of Thioketals with *Cp*VBPO (General Procedure D):

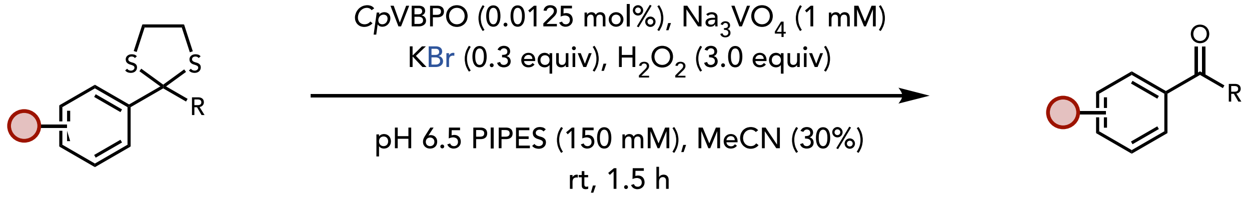

An enzyme aliquot of the VHPO from *Corallina pilulifera* (*Cp*VBPO, 10 µM, 10 mL) was removed from a -80 °C freezer and allowed to warm to room temperature over 10 min. After thawing, the enzyme aliquot was combined with 250 mM solution of aqueous Na_3VO_4 (800 µL). This solution was then allowed to sit at room temperature for 30 minutes. To a 250 mL round bottom flask containing a magnetic stir bar was added H_2O purified by an Elga purification system (67.0 mL), 500 mM pH 6.5 PIPES buffer (60 mL) and 176 mM aqueous KBr (1.36 mL, 0.3 equiv). This was followed by the addition of 40 mL MeCN, a solution of the corresponding dithiolane substrate (1.0 equiv, 0.800 mmol) in MeCN (10 mL), and an additional 10 mL of MeCN. The contents of the centrifuge tube containing *Cp*VBPO (0.0125 mol%, 1 µM in reaction) and Na_3VO_4 (0.25 equiv) were added to the reaction mixture followed by a 10% stock of H_2O_2 (790 µL, 3 equiv). The reaction was then left to stir at room temperature at 900 rpm for 1.5 hr. After this time, the reaction mixture was concentrated to remove the MeCN and transferred to a separatory funnel. Additional H_2O (60 mL) was added, and the mixture was extracted with ethyl acetate (3 x 75 mL). The combined organic layers were then washed with brine (100 mL), dried over sodium sulfate, and concentrated under reduced pressure. The resulting crude sample was purified on a silica gel hand column to obtain the pure product.

This Procedure was also used for the VHPO-catalyzed hydrolysis of substrates **(29) & **(30)**.

Product Characterization for Carbonyl Compounds

Acetophenone (2)[2]

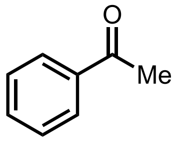

Synthesized from 2-Methyl-2-phenyl-1,3-dithiolane (1) following General Procedure D and was obtained as a colorless liquid.

Yield: 93% (89.3 mg)

Purification: Eluted in 10% EtOAc in Hexanes

^{1}H NMR (400 MHz, $CDCl_3$) δ 7.95 (dt, *J* = 7.0, 1.5 Hz, 2H), 7.59 – 7.52 (m, 1H), 7.45 (ddd, *J* = 8.2, 6.6, 1.3 Hz, 2H), 2.60 (d, *J* = 1.4 Hz, 3H).

^{13}C NMR (101 MHz, $CDCl_3$) δ 198.3, 137.2, 133.2, 128.7, 128.4, 26.7.

Standard Curve for Analytical Runs:
Procedure for using standard curve is as follows: 1,3,5-tribromobenzene (8 mg/mL solution, 100 µL) is added to 900 µL of the reaction mixture and yield is determined by LCMS analysis based on the below standard curve. LCMS conditions: 2.5 µL injection volume, 0.5 mL/min mobile phase rate, 10-98% solvent B over 6.25 min. Mobile Phase: Solvent A – H_2O w/ 0.1% formic acid, Solvent B – MeCN w/ 0.1% formic acid.

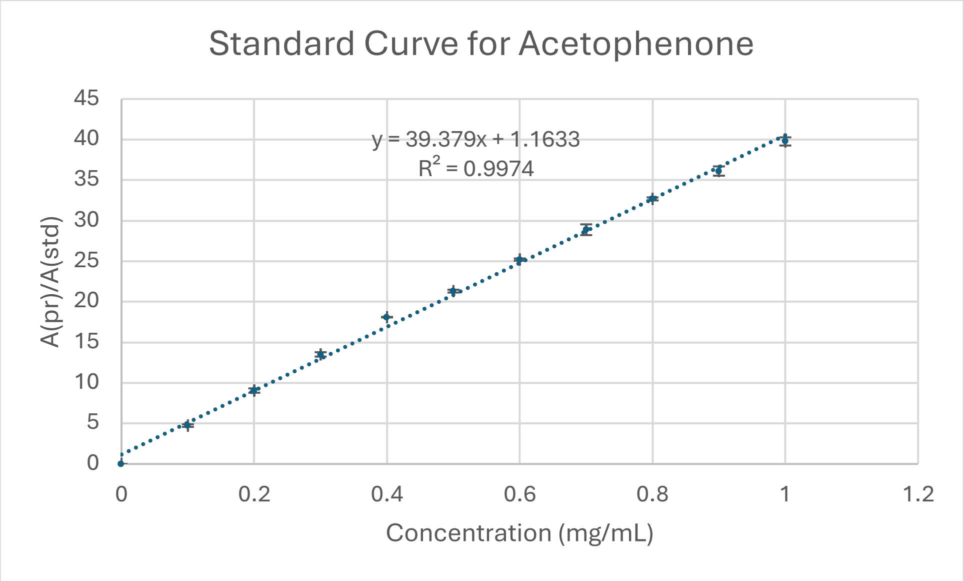

1-(4-Bromophenyl)ethan-1-one (3)[2]

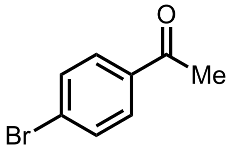

Synthesized from 2-(4-Bromophenyl)-2-methyl-1,3-dithiolane (SM-2) following General Procedure D and was obtained as a white solid.

Yield: 92% (146.3 mg)

Purification: Eluted in 10% EtOAc in Hexanes

^{1}H NMR (400 MHz, $CDCl_3$) δ 7.85 – 7.78 (m, 2H), 7.64 – 7.56 (m, 2H), 2.58 (s, 3H).

^{13}C NMR (101 MHz, $CDCl_3$) δ 197.0, 135.9, 131.9, 129.9, 128.3, 26.6.

1-(4-Chlorophenyl)ethan-1-one (4)[2]

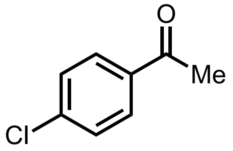

Synthesized from 2-(4-Chlorophenyl)-2-methyl-1,3-dithiolane (SM-3) following General Procedure D and was obtained as a colorless liquid.

Yield: 91% (112.5 mg)

Purification: Eluted in 10% EtOAc in Hexanes

^{1}H NMR (400 MHz, $CDCl_3$) δ 7.88 (d, J = 8.7 Hz, 2H), 7.42 (d, J = 8.6 Hz, 2H), 2.58 (s, 3H).

^{13}C NMR (101 MHz, $CDCl_3$) δ 197.0, 139.7, 135.5, 129.8, 129.0, 26.7.

1-(4-Fluorophenyl)ethan-1-one (5)[2]

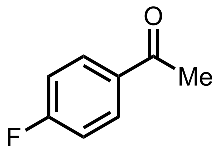

Synthesized from 2-(4-Fluorophenyl)-2-methyl-1,3-dithiolane (SM-4) following General Procedure D and was obtained as a colorless liquid.

Yield: 89% (98.1 mg)

Purification: Eluted in 10% EtOAc in Hexanes

^{1}H NMR (400 MHz, $CDCl_3$) δ 8.02 – 7.91 (m, 2H), 7.16 – 7.07 (m, 2H), 2.57 (s, 3H).

^{13}C NMR (101 MHz, $CDCl_3$) δ 196.6, 165.8 (d, J = 255.8 Hz), 133.7 (d, J = 3.0 Hz), 131.0 (d, J = 9.3 Hz), 115.7 (d, J = 22.0 Hz), 26.6.

1-(4-Methoxyphenyl)ethan-1-one (6)[14]

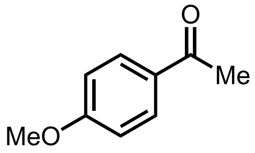

Synthesized from 2-(4-Methoxyphenyl)-2-methyl-1,3-dithiolane (SM-5) following General Procedure D and was obtained as a white solid.

Yield: 90% (108.2 mg)

Purification: Eluted in 10% EtOAc in Hexanes

^{1}H NMR (400 MHz, $CDCl_3$) δ 8.02 – 7.90 (m, 2H), 7.02 – 6.92 (m, 2H), 3.89 (s, 3H), 2.58 (s, 3H).

^{13}C NMR (101 MHz, $CDCl_3$) δ 196.9, 163.5, 130.6, 130.3, 113.7, 55.5, 26.4.

1-(*p*-Tolyl)ethan-1-one (7)[14]

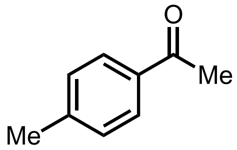

Synthesized from 2-Methyl-2-(p-tolyl)-1,3-dithiolane (SM-6) following General Procedure D and was obtained as a colorless liquid.

Yield: 91% (96.7 mg)

Purification: Eluted in 10% EtOAc in Hexanes

^{1}H NMR (400 MHz, $CDCl_3$) δ 7.95 – 7.79 (m, 2H), 7.29 – 7.22 (m, 2H), 2.57 (s, 3H), 2.40 (s, 3H).

^{13}C NMR (101 MHz, $CDCl_3$) δ 198.0, 144.0, 134.7, 129.3, 128.5, 26.6, 21.7.

1-(4-(*tert*-Butyl)phenyl)ethan-1-one (8)[15]

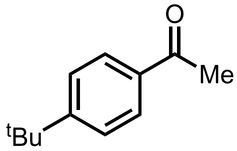

Synthesized from 2-(4-(tert-Butyl)phenyl)-2-methyl-1,3-dithiolanemide (SM-7) following General Procedure D and was obtained as a colorless liquid.

Yield: 90% (126.5 mg)

Purification: Eluted in 10% EtOAc in Hexanes

^{1}H NMR (400 MHz, $CDCl_3$) δ 7.93 (d, J = 8.5 Hz, 2H), 7.50 (d, J = 8.5 Hz, 2H), 2.61 (s, 3H), 1.37 (s, 9H).

^{13}C NMR (101 MHz, $CDCl_3$) δ 198.0, 156.9, 134.6, 128.3, 125.5, 35.1, 31.1, 26.6.

1-(4-Nitrophenyl)ethan-1-one (9)[16]

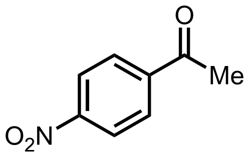

Synthesized from 2-Methyl-2-(4-nitrophenyl)-1,3-dithiolane (SM-8) following General Procedure D with the reaction run for 2 hours and was obtained as a light orange solid.

Yield: 89% (117.1 mg)

Purification: Eluted in 15% EtOAc in Hexanes

^{1}H NMR (400 MHz, $CDCl_3$) δ 8.32 – 8.27 (m, 2H), 8.12 – 8.08 (m, 2H), 2.67 (s, 3H).

^{13}C NMR (101 MHz, $CDCl_3$) δ 196.4, 150.5, 141.5, 129.4, 124.0, 27.1.

1-([1,1'-Biphenyl]-4-yl)ethan-1-one (10)[17]

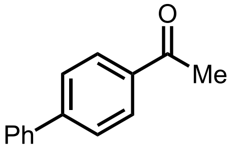

Synthesized from 2-([1,1'-Biphenyl]-4-yl)-2-methyl-1,3-dithiolane (SM-9) following General Procedure D with the reaction run for 2 hours and was obtained as a light brown solid.

Yield: 86% (134.8 mg)

Purification: Eluted in 10% EtOAc in Hexanes

^{1}H NMR (400 MHz, $CDCl_3$) δ 8.04 (d, J = 8.2 Hz, 2H), 7.66 (dd, J = 23.4, 7.8 Hz, 4H), 7.44 (dt, J = 28.8, 7.3 Hz, 3H), 2.65 (s, 3H).

^{13}C NMR (101 MHz, $CDCl_3$) δ 198.1, 145.9, 140.0, 136.1, 129.2, 129.1, 128.4, 127.6, 127.4, 127.4, 26.9.

Methyl 4-acetylbenzoate (11)[15]

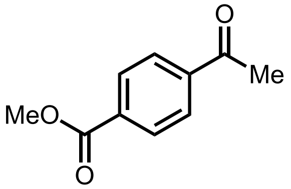

Synthesized from Methyl 4-(2-methyl-1,3-dithiolan-2-yl)benzoate (SM-10) following General Procedure D and was obtained as a light yellow solid.

Yield: 88% (125.0 mg)

Purification: Eluted in 15% EtOAc in Hexanes

^{1}H NMR (400 MHz, $CDCl_3$) δ 8.15 – 8.10 (m, 2H), 8.03 – 7.98 (m, 2H), 3.95 (s, 3H), 2.64 (s, 3H).

^{13}C NMR (101 MHz, $CDCl_3$) δ 197.7, 166.4, 140.3, 134.0, 130.0, 128.4, 52.6, 27.06.

1-(3-Methoxyphenyl)ethan-1-one (12)[14]

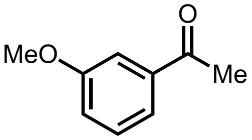

Synthesized from 2-(3-Methoxyphenyl)-2-methyl-1,3-dithiolane (SM-11) following General Procedure D and was obtained as a colorless liquid.

Yield: 90% (108.3 mg)

Purification: Eluted in 10% EtOAc in Hexanes

^{1}H NMR (400 MHz, $CDCl_3$) δ 7.52 (ddd, J = 7.6, 1.6, 1.0 Hz, 1H), 7.47 (dd, J = 2.7, 1.6 Hz, 1H), 7.35 (t, J = 7.9 Hz, 1H), 7.09 (ddd, J = 8.2, 2.7, 1.0 Hz, 1H), 3.84 (s, 3H), 2.58 (s, 3H).

^{13}C NMR (101 MHz, $CDCl_3$) δ 198.0, 159.9, 138.6, 129.6, 121.2, 119.7, 112.4, 55.5, 26.8.

1-(*m*-Tolyl)ethan-1-one (13)[14]

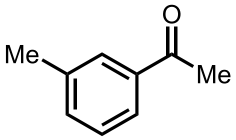

Synthesized from 2-Methyl-2-(m-tolyl)-1,3-dithiolane (SM-12) following General Procedure D and was obtained as a colorless liquid.

Yield: 91% (97.9 mg)

Purification: Eluted in 10% EtOAc in Hexanes

^{1}H NMR (400 MHz, $CDCl_3$) δ 7.82 – 7.70 (m, 2H), 7.40 – 7.31 (m, 2H), 2.59 (s, 3H), 2.41 (d, *J* = 0.9 Hz, 3H).

^{13}C NMR (101 MHz, $CDCl_3$) δ 198.5, 138.5, 137.3, 134.0, 128.9, 128.6, 125.7, 26.8, 21.4.

1-(*o*-Tolyl)ethan-1-one (14)[14]

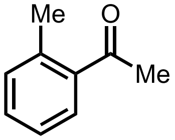

Synthesized from 2-Methyl-2-(o-tolyl)-1,3-dithiolane (SM-13) following General Procedure D and was obtained as a colorless liquid.

Yield: 87% (94.1 mg)

Purification: Eluted in 10% EtOAc in Hexanes

^{1}H NMR (400 MHz, $CDCl_3$) δ 7.70 (dd, *J* = 7.7, 1.4 Hz, 1H), 7.41 – 7.35 (m, 1H), 7.29 – 7.23 (m, 2H), 2.59 (s, 3H), 2.53 (s, 3H).

^{13}C NMR (101 MHz, $CDCl_3$) δ 201.9, 138.5, 137.8, 132.2, 131.7, 129.5, 125.8, 29.7, 21.7.

1-(2-Methoxyphenyl)ethan-1-one (15)[14]

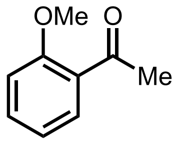

Synthesized from 2-(2-Methoxyphenyl)-2-methyl-1,3-dithiolanebenzamide (SM-14) following General Procedure D and was obtained as a colorless liquid.

Yield: 90% (107.9 mg)

Purification: Eluted in 10% EtOAc in Hexanes

^{1}H NMR (400 MHz, $CDCl_3$) δ 7.73 (dd, J = 7.7, 1.9 Hz, 1H), 7.46 (ddd, J = 8.3, 7.3, 1.8 Hz, 1H), 7.02 – 6.94 (m, 2H), 3.90 (s, 3H), 2.61 (s, 3H).

^{13}C NMR (101 MHz, $CDCl_3$) δ 200.1, 159.0, 133.8, 130.4, 120.6, 111.6, 55.6, 32.0.

1-(Thiophen-2-yl)ethan-1-one (16)[2]

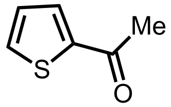

Synthesized from 2-Methyl-2-(thiophen-2-yl)-1,3-dithiolane (SM-15) following General Procedure D and was obtained as a colorless liquid.

Yield: 90% (90.8 mg)

Purification: Eluted in 10% EtOAc in Hexanes

^{1}H NMR (400 MHz, $CDCl_3$) δ 7.69 (dd, J = 3.7, 1.2 Hz, 1H), 7.63 (dd, J = 4.9, 1.2 Hz, 1H), 7.12 (dd, J = 5.0, 3.7 Hz, 1H), 2.56 (s, 3H).

^{13}C NMR (101 MHz, $CDCl_3$) δ 190.9, 144.7, 133.9, 132.6, 128.2, 27.1.

Phenyl(thiophen-2-yl)methanone (17)[2]

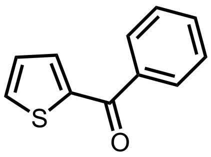

Synthesized from 2-Phenyl-2-(thiophen-2-yl)-1,3-dithiolane (SM-16) following General Procedure D and was obtained as a white solid.

Yield: 89% (134.0 mg)

Purification: Eluted in 10% EtOAc in Hexanes

^{1}H NMR (400 MHz, $CDCl_3$) δ 7.89 – 7.84 (m, 2H), 7.73 (dd, J = 4.9, 1.1 Hz, 1H), 7.65 (dd, J = 3.8, 1.2 Hz, 1H), 7.63 – 7.57 (m, 1H), 7.53 – 7.47 (m, 2H), 7.17 (dd, J = 5.0, 3.8 Hz, 1H).

^{13}C NMR (101 MHz, $CDCl_3$) δ 188.4, 143.8, 138.2, 135.0, 134.4, 132.4, 129.3, 128.6, 128.1.

1-(Naphthalen-2-yl)ethan-1-one (18)[2]

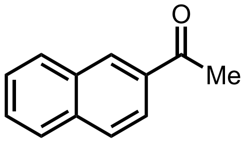

Synthesized from 2-Methyl-2-(naphthalen-2-yl)-1,3-dithiolane (SM-17) following General Procedure D and was obtained as a white solid.

Yield: 90% (122.1 mg)

Purification: Eluted in 10% EtOAc in Hexanes

^{1}H NMR (400 MHz, $CDCl_3$) δ 8.49 – 8.46 (m, 1H), 8.04 (dd, J = 8.7, 1.8 Hz, 1H), 7.99 – 7.95 (m, 1H), 7.91 – 7.86 (m, 2H), 7.58 (dddd, J = 19.4, 8.1, 6.9, 1.4 Hz, 2H), 2.73 (s, 3H).

^{13}C NMR (101 MHz, $CDCl_3$) δ 198.3, 135.7, 134.6, 132.7, 130.3, 129.7, 128.6, 127.9, 126.9, 124.0, 26.8.

1-(2,3-Dihydrobenzofuran-5-yl)ethan-1-one (19)[18]

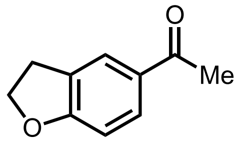

Synthesized from 5-(2-Methyl-1,3-dithiolan-2-yl)-2,3-dihydrobenzofuran (SM-18) following General Procedure D and was obtained as a white solid.

Yield: 91% (117.8 mg)

Purification: Eluted in 10% EtOAc in Hexanes

^{1}H NMR (400 MHz, $CDCl_3$) δ 7.84 (q, J = 1.5 Hz, 1H), 7.79 (dt, J = 8.4, 1.4 Hz, 1H), 6.79 (d, J = 8.4 Hz, 1H), 4.65 (t, J = 8.8 Hz, 2H), 3.24 (t, J = 8.8 Hz, 2H), 2.53 (s, 3H).

^{13}C NMR (101 MHz, $CDCl_3$) δ 196.8, 164.5, 130.7, 130.5, 125.6, 109.0, 72.3, 29.1, 26.6.

3,4-Dihydronaphthalen-1(2H)-one (20)[2]

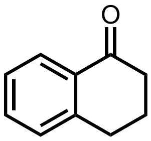

Synthesized from 3,4-Dihydro-2H-spiro[naphthalene-1,2'-[1,3]dithiolane] (SM-19) following General Procedure D and was obtained as a colorless liquid.

Yield: 93% (108.3 mg)

Purification: Eluted in 10% EtOAc in Hexanes

^{1}H NMR (400 MHz, $CDCl_3$) δ 8.03 (dd, J = 7.8, 1.5 Hz, 1H), 7.46 (td, J = 7.5, 1.5 Hz, 1H), 7.33 – 7.23 (m, 2H), 2.96 (t, J = 6.1 Hz, 2H), 2.65 (dd, J = 7.3, 5.8 Hz, 2H), 2.14 (p, J = 6.5 Hz, 2H).

^{13}C NMR (101 MHz, $CDCl_3$) δ 198.5, 144.6, 133.5, 132.7, 128.9, 127.3, 126.7, 39.3, 29.8, 23.4.

Propiophenone (21)[17]

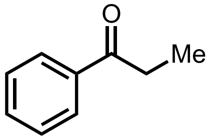

Synthesized from 2-Ethyl-2-phenyl-1,3-dithiolane (SM-20) following General Procedure D and was obtained as a colorless liquid.

Yield: 92% (98.1 mg)

Purification: Eluted in 10% EtOAc in Hexanes

^{1}H NMR (400 MHz, $CDCl_3$) δ 7.99 – 7.93 (m, 2H), 7.58 – 7.52 (m, 1H), 7.48 – 7.43 (m, 2H), 3.01 (q, J = 7.2 Hz, 2H), 1.23 (t, J = 7.2 Hz, 3H).

^{13}C NMR (101 MHz, $CDCl_3$) δ 201.0, 137.0, 133.0, 128.7, 128.1, 31.9, 8.4.

4-Phenylcyclohexan-1-one (22)[2]

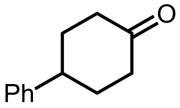

Synthesized from 8-Phenyl-1,4-dithiaspiro[4.5]decane (SM-21) following General Procedure D and was obtained as a white solid.

Yield: 86% (119.4 mg)

Purification: Eluted in 10% EtOAc in Hexanes

^{1}H NMR (400 MHz, $CDCl_3$) δ 7.37 – 7.30 (m, 2H), 7.28 – 7.22 (m, 3H), 3.04 (tt, J = 12.1, 3.4 Hz, 1H), 2.52 (dd, J = 11.1, 4.9 Hz, 4H), 2.29 – 2.19 (m, 2H), 2.04 – 1.88 (m, 2H).

^{13}C NMR (101 MHz, $CDCl_3$) δ 211.3, 144.9, 128.7, 126.8, 126.7, 42.9, 41.5, 34.1.

Benzaldehyde (23)[19]

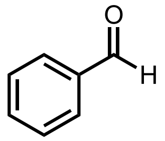

Synthesized from 2-Phenyl-1,3-dithiolane (SM-22) following General Procedure D and was obtained as a colorless liquid.

Yield: 92% (78.6 mg)

Purification: Eluted in 10% EtOAc in Hexanes

^{1}H NMR (400 MHz, $CDCl_3$) δ 10.03 (s, 1H), 7.93 – 7.84 (m, 2H), 7.67 – 7.61 (m, 1H), 7.57 – 7.51 (m, 2H).

^{13}C NMR (101 MHz, $CDCl_3$) δ 192.6, 136.6, 134.6, 129.9, 129.1.

4-Hydroxy-3-methoxybenzaldehyde (24)[19]

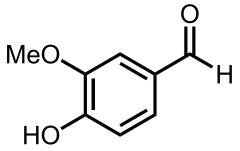

Synthesized from 4-(1,3-dithiolan-2-yl)-2-methoxyphenol (SM-23) following General Procedure D and was obtained as a white solid.

Yield: 89% (108.5 mg)

Purification: Eluted in 30% EtOAc in Hexanes

^{1}H NMR (400 MHz, $CDCl_3$) δ 9.82 (s, 1H), 7.45 – 7.34 (m, 2H), 7.04 (d, J = 8.5 Hz, 1H), 6.28 (s, 1H), 3.96 (s, 3H).

^{13}C NMR (101 MHz, $CDCl_3$) δ 191.1, 151.8, 147.3, 130.0, 114.5, 108.9, 56.3.

4-(Dimethylamino)benzaldehyde (25)[20]

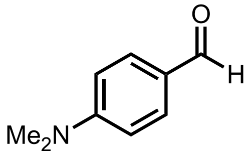

Synthesized from 4-(1,3-dithiolan-2-yl)-N,N-dimethylaniline (SM-24) following General Procedure D and was obtained as a blue solid.

Yield: 86% (102.9 mg)

Purification: Eluted in 15% EtOAc in Hexanes

^{1}H NMR (400 MHz, $CDCl_3$) δ 9.74 (s, 1H), 7.81 – 7.66 (m, 2H), 6.75 – 6.64 (m, 2H), 3.08 (s, 6H).

^{13}C NMR (101 MHz, $CDCl_3$) δ 190.5, 154.5, 132.1, 125.3, 111.1, 40.2.

Nicotinaldehyde (26)[17]

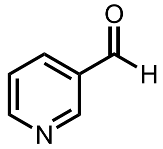

Synthesized from 3-(1,3-dithiolan-2-yl)pyridine (SM-24) following General Procedure D with the reaction run for 2 hours and was obtained as a yellow liquid.

Yield: 88% (75.7 mg)

Purification: Eluted in 60% EtOAc in Hexanes

^{1}H NMR (400 MHz, $CDCl_3$) δ 10.11 (s, 1H), 9.07 (dd, J = 2.2, 0.9 Hz, 1H), 8.84 (dd, J = 4.8, 1.8 Hz, 1H), 8.17 (dt, J = 7.9, 2.0 Hz, 1H), 7.48 (ddt, J = 7.9, 4.9, 0.7 Hz, 1H).

^{13}C NMR (101 MHz, $CDCl_3$) δ 190.9, 154.9, 152.3, 135.9, 131.5, 124.2.

1H-indole-3-carbaldehyde (27)[2]

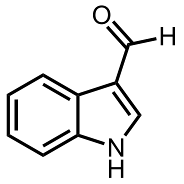

Synthesized from 3-(1,3-dithiolan-2-yl)-1H-indole (SM-24) following General Procedure D and was obtained as a light yellow solid.

Yield: 85% (116.1 mg)

Purification: Eluted in 30% EtOAc in Hexanes

^{1}H NMR (400 MHz, DMSO-d_6) δ 12.12 (s, 1H), 9.93 (s, 1H), 8.27 (s, 1H), 8.08 (dt, J = 7.5, 1.0 Hz, 1H), 7.50 (dt, J = 8.0, 0.9 Hz, 1H), 7.32 – 7.13 (m, 2H).

^{13}C NMR (101 MHz, DMSO-d_6) δ 184.9, 138.4, 137.0, 124.1, 123.4, 120.8, 118.2, 112.4.

Procedure for the Preparation of 2-Methyl-2-phenyl-1,3-dioxolane (28)

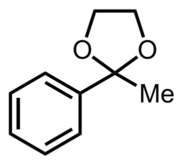

The following was adapted according to a literature procedure.[21] To a solution of acetophenone (0.58 mL, 5 mmol, 1.0 equiv), triethyl orthoformate (1.83 mL, 11 mmol, 2.2 equiv) and ethylene glycol (1.12 mL, 20 mmol, 4.0 equiv) was added tetrabutyl ammonium tribromide (24.1 mg, 0.05 mmol, 0.1 equiv). The homogenous reaction was allowed to stir at room temperature for 1 h. After the indicated time reaction mixture was quenched using saturated aqueous $NaHCO_3$ (10 mL) and extracted with ethyl acetate (3 x 25 mL). The combined organic extracts were washed with 30 mL of brine, then dried over anhydrous sodium sulfate, and concentrated under reduced pressure. The resulting crude residue was purified by flash column chromatography, yielding the desired product.

Yield: 67% (550.1 mg)

Purification: Eluted in 10% EtOAc in Hexanes

^{1}H NMR (400 MHz, $CDCl_3$) δ 7.54 – 7.43 (m, 2H), 7.38 – 7.26 (m, 3H), 4.09 – 3.99 (m, 2H), 3.83 – 3.73 (m, 2H), 1.66 (s, 3H).

^{13}C NMR (101 MHz, $CDCl_3$) δ 143.4, 128.3, 128.0, 125.4, 109.0, 64.6, 27.8.

Additional Reaction Procedures

Gram Scale Procedure for the Biocatalytic Hydrolysis of Thioketals (General Procedure E):

Seven enzyme aliquots of the VHPO from *Corallina pilulifera* (*Cp*VBPO, 10.0 μM, 10.0 mL) were removed from a -80 °C freezer and allowed to warm to room temperature over 10 min. After thawing the enzyme aliquots, 70 mL solution of *Corallina pilulifera* (*Cp*VBPO, 10.0 μM) was transferred to a 250 mL round bottom flask and combined with 250 mM solution of aqueous Na_3VO_4 (5.60 mL). This solution was then allowed to stir at room temperature for 30 minutes. To a 2 L round bottom flask containing a magnetic stir bar was added H_2O purified by an Elga purification system (469 mL), 500 mM pH 6.5 PIPES buffer (420 mL) and 176 mM aqueous KBr (9.52 mL, 0.3 equiv). This was followed by the addition of 300 mL MeCN, a solution of 2-methyl-2-phenyl-1,3-dithiolane (1.10 g, 5.6 mmol) in MeCN (100 mL), and an additional 20 mL of MeCN. The contents of the 250 mL round bottom flask containing *Cp*VBPO (0.025 mol%, 1 μM in reaction) and Na_3VO_4 (0.25 equiv) were added to the reaction mixture followed by a 10% stock of H_2O_2 (5.50 mL, 3.0 equiv). The reaction was then left to stir at room temperature at 900 rpm for 1.5 hr. After this time, the reaction mixture was concentrated to remove the MeCN and transferred to a separatory funnel. Additional H_2O (100 mL) was added, and the mixture was extracted with ethyl acetate (3 x 200 mL). The combined organic layers were then washed with brine (100 mL), dried over sodium sulfate, and concentrated under reduced pressure. The resulting crude sample was purified on a silica gel hand column to obtain the pure acetophenone (2) as a colorless liquid in 90% yield, 0.61 g.

General Analytical Procedure for the Biocatalytic Hydrolysis of Thioketals using Wet Cell Lysate Expressing *Cp*VBPO (General Procedure F):

A wet cell lysate aliquot containing VHPO from *Corallina pilulifera* (*Cp*VBPO, 10μL) was removed from a -80 °C freezer and warmed to room temperature over 5 min. After thawing, a 250 mM solution of aqueous Na_3VO_4 (4 μL) was added to the cell lysate aliquot, and the resulting mixture was centrifuged for 10 seconds using a Chemglass Life Sciences MLX-108-CLS mini centrifuge and then placed at room temperature until further use. To a 1-dram vial was then added H_2O purified by an Elga purification system (375.2 μL), 500 mM pH 6.5 PIPES buffer (300 μL), and 176 mM aqueous KBr (6.82 μL, 0.3 equiv) followed by addition of 200 μL MeCN. A 40 mM solution of the dithiolane substrate in MeCN (100 μL, 1.0 equiv, 0.004 mmol substrate) was then added. The cell lysate aliquot containing the *Cp*VBPO (10 μL) and Na_3VO_4 (0.25 equiv) was then added to the reaction mixture followed by the addition of a 10% stock of H_2O_2 (3.95 μL, 3.0 equiv). The vial was then capped and placed on a shaker at room temperature for 1.5 hr. After this time, the reaction mixture was diluted with MeCN (650 μL), transferred to an Eppendorf tube, and centrifuged in a Benchmark MC-24™ Touch Centrifuge at 12,500 rpm for 5 min. After centrifugation, 650 μL of the top layer of the reaction mixture was transferred to an LCMS vial, which was then placed on an LCMS for analysis.*

**100 μL of 8 mg/mL solution of 1,3,5-tribromobenzene was added as an internal standard for yield confirmation, where applicable.*

General Analytical Procedure for the Biocatalytic Hydrolysis of Thioketals using Whole Cells Expressing *Cp*VBPO (General Procedure G):

An aliquot containing whole *E. coli* cell expressing VHPO from *Corallina pilulifera* (*Cp*VBPO, 10 μL, adjusted to OD_{600} = 18.5) was removed from a -80 °C freezer and warmed to room temperature over 5 min. After thawing, a 250 mM solution of aqueous Na_3VO_4 (4 μL) was added to the whole cell aliquot, and the resulting mixture was centrifuged for 10 seconds using a Chemglass Life Sciences MLX-108-CLS mini centrifuge and then placed at room temperature until further use. To a 1-dram vial was then added H_2O purified by an Elga purification system (375.2 μL), 500 mM pH 6.5 PIPES buffer (300 μL), and 176 mM aqueous KBr (6.82 μL, 0.3 equiv) followed by addition of 200 μL MeCN. A 40 mM solution of the dithiolane substrate in MeCN (100 μL, 1.0 equiv, 0.004 mmol substrate) was then added. The aliquot containing the whole *E. coli* cells expressing *Cp*VBPO (10 μL, adjusted to OD_{600} = 18.5) and Na_3VO_4 (0.25 equiv) was then added to the reaction mixture followed by the addition of a 10% stock of H_2O_2 (3.95 μL, 3.0 equiv). The vial was then capped and placed on a shaker at room temperature for 1.5 hr. After this time, the reaction mixture was diluted with MeCN (650 μL), transferred to an Eppendorf tube, and centrifuged in a Benchmark MC-24™ Touch Centrifuge at 12,500 rpm for 5 min. After centrifugation, 650 μL of the top layer of the reaction mixture was transferred to an LCMS vial, which was then placed on an LCMS for analysis.*

**100 μL of 8 mg/mL solution of 1,3,5-tribromobenzene was added as an internal standard for yield confirmation, where applicable.*

General Analytical Procedure for the Biocatalytic Hydrolysis of Thioketals using Lyophilized Cell Lysate Expressing *Cp*VBPO (General Procedure H):

To a PCR tube, 0.6 mg of lyophilized cell lysate was added followed by addition of 500 mM pH 6.5 PIPES buffer (100 μL) and 250 mM solution of aqueous Na_3VO_4 (4 μL). The resulting mixture was centrifuged for 10 seconds using a Chemglass Life Sciences MLX-108-CLS mini centrifuge and then placed at room temperature until further use. To a 1-dram vial was then added H_2O purified by an Elga purification system (385.2 μL), 500 mM pH 6.5 PIPES buffer (200 μL), and 176 mM aqueous KBr (6.82 μL, 0.3 equiv) followed by addition of 200 μL MeCN. A 40 mM solution of the dithiolane substrate in MeCN (100 μL, 1.0 equiv, 0.004 mmol substrate) was then added. The aliquot containing the lyophilized cell lysate and Na_3VO_4 (0.25 equiv) was then added to the reaction mixture followed by the addition of a 10% stock of H_2O_2 (3.95 μL, 3.0 equiv). The vial was then capped and placed on a shaker at room temperature for 1.5 hr. After this time, the reaction mixture was diluted with MeCN (650 μL), transferred to an Eppendorf tube, and

centrifuged in a Benchmark MC-24TM Touch Centrifuge at 12,500 rpm for 5 min. After centrifugation, 650 µL of the top layer of the reaction mixture was transferred to an LCMS vial, which was then placed on an LCMS for analysis.*

**100 µL of 8 mg/mL solution of 1,3,5-tribromobenzene was added as an internal standard for yield confirmation, where applicable.*

General Analytical Procedure for the Biocatalytic Hydrolysis of Thioketals using Lyophilized Whole Cells Expressing *Cp*VBPO (General Procedure I):

To a PCR tube, 0.8 mg of lyophilized whole cell was added followed by addition of of 500 mM pH 6.5 PIPES buffer (100 µL) and 250 mM solution of aqueous Na_3VO_4 (4 µL). The resulting mixture was centrifuged for 10 seconds using a Chemglass Life Sciences MLX-108-CLS mini centrifuge and then placed at room temperature until further use. To a 1-dram vial was then added H_2O purified by an Elga purification system (385.2 µL), 500 mM pH 6.5 PIPES buffer (200 µL), and 176 mM aqueous KBr (6.82 µL, 0.3 equiv) followed by addition of 200 µL MeCN. A 40 mM solution of the dithiolane substrate in MeCN (100 µL, 1.0 equiv, 0.004 mmol substrate) was then added. The aliquot containing the lyophilized whole cells and Na_3VO_4 (0.25 equiv) was then added to the reaction mixture followed by the addition of a 10% stock of H_2O_2 (3.95 µL, 3.0 equiv). The vial was then capped and placed on a shaker at room temperature for 1.5 hr. After this time, the reaction mixture was diluted with MeCN (650 µL), transferred to an Eppendorf tube, and centrifuged in a Benchmark MC-24TM Touch Centrifuge at 12,500 rpm for 5 min. After centrifugation, 650 µL of the top layer of the reaction mixture was transferred to an LCMS vial, which was then placed on an LCMS for analysis.*

**100 µL of 8 mg/mL solution of 1,3,5-tribromobenzene was added as an internal standard for yield confirmation, where applicable.*

Procedure for the Preparation of 2-Methyl-2-phenyl-1,3-dithiane (29)

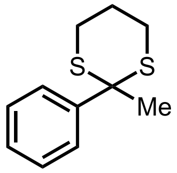

The following was adapted according to a literature procedure.[2] A solution of acetophenone (8 mmol, 1.0 equiv) in CH_2Cl_2 (40 mL, 0.2 M) was prepared. To this solution 1,3-propanedithiol (0.80 mL, 8 mmol, 1.0 equiv) and BF_3•OEt_2 (1.97 mL, 16 mmol, 2.0 equiv) were added at room temperature. The solution was allowed to stir at room temperature for 5 h. After the indicated time, the reaction mixture was quenched using saturated aqueous $NaHCO_3$ (100 mL) and extracted CH_2Cl_2 (3 x 50 mL). The combined organic extracts were washed with 50 mL of brine, then dried over anhydrous sodium sulfate, and concentrated under reduced pressure. The resulting crude residue was purified by flash column chromatography, yielding 2-Methyl-2-phenyl-1,3-dithiane as a colorless liquid.

Yield: 71% (1.19 g)

Purification: Eluted in 10% EtOAc in Hexanes

^{1}H NMR (400 MHz, $CDCl_3$) δ 7.97 – 7.92 (m, 2H), 7.41 – 7.35 (m, 2H), 7.28 – 7.24 (m, 1H), 2.77 – 2.68 (m, 4H), 1.94 (ddt, *J* = 7.6, 6.3, 3.8 Hz, 2H), 1.80 (s, 3H).

^{13}C NMR (101 MHz, $CDCl_3$) δ 143.9, 128.7, 127.9, 127.2, 54.1, 32.9, 28.2, 24.8.

Procedure for the Preparation of 2-Methyl-2-phenyl-1,3-oxathiolane (30)

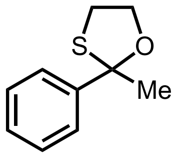

The following was adapted according to a literature procedure.[22] A solution of acetophenone (2.33 mL, 20 mmol, 1.0 equiv) in ether (25 mL, 0.8 M). To this solution 2-mercaptoethanol (1.76 mL, 25 mmol, 1.25 equiv) and BF_3•OEt_2 (2.46 mL, 20 mmol, 1.0 equiv) were added at room temperature. The solution was refluxed using sand bath for 16 h. After the indicated time, the reaction mixture was quenched using saturated aqueous $NaHCO_3$ (100 mL) and extracted ethyl acetate (3 x 100 mL). The combined organic extracts were washed with100 mL of brine, then dried over anhydrous sodium sulfate, and concentrated under reduced pressure. The resulting crude residue was purified by flash column chromatography to yield 2-Methyl-2-phenyl-1,3-oxathiolane as a colorless liquid.

Yield: 60% (2.16 g)

Purification: Eluted in 5% EtOAc in Hexanes

^{1}H NMR (400 MHz, $CDCl_3$) δ 7.56 – 7.45 (m, 2H), 7.33 (s, 2H), 7.27 – 7.22 (m, 1H), 4.36 (ddd, *J* = 9.2, 6.3, 4.5 Hz, 1H), 4.03 (ddd, *J* = 9.2, 7.5, 6.0 Hz, 1H), 3.24 (ddd, *J* = 10.0, 7.5, 6.3 Hz, 1H), 3.09 (ddd, *J* = 10.2, 6.0, 4.5 Hz, 1H), 1.92 (s, 3H).

^{13}C NMR (101 MHz, $CDCl_3$) δ 146.9, 128.3, 127.4, 125.0, 95.8, 70.8, 34.6, 32.5.

<u>Optimization Data</u>

All optimization reactions were performed in triplicate using the Analytical Procedure for the hydrolysis of thioketals with *Cp*VBPO (General Procedure C– located in main manuscript). The only variable changed is the one indicated in the Figures below. (*Note: 100 µL of an 8 mg/mL solution of 1,3,5-tribromobenzene was added as an internal standard for yield confirmation, where applicable)*

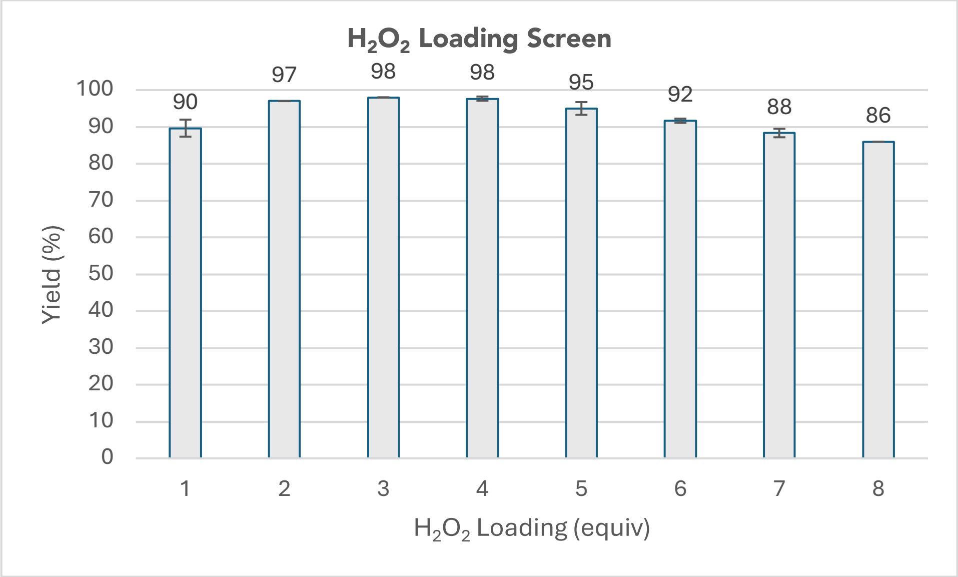

Figure S1. Hydrogen Peroxide (H_2O_2) Loading Screen

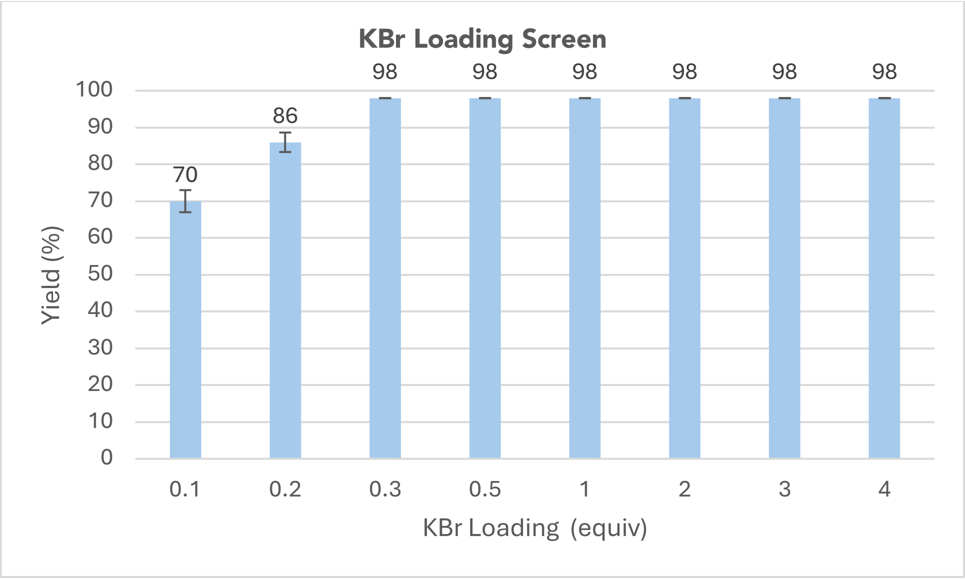

Figure S2. Potassium Bromide (KBr) Loading Screen

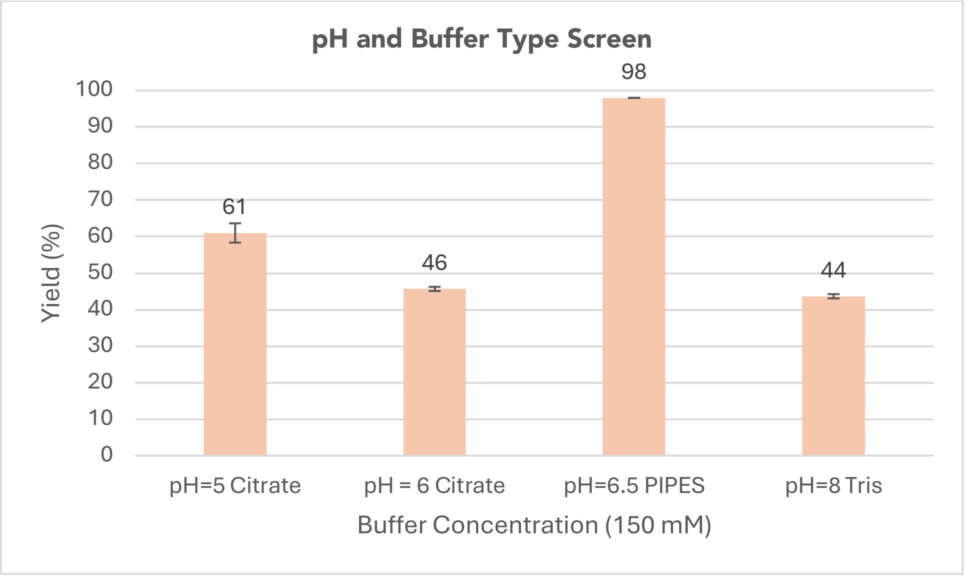

Figure S3. pH and Buffer Type Screen

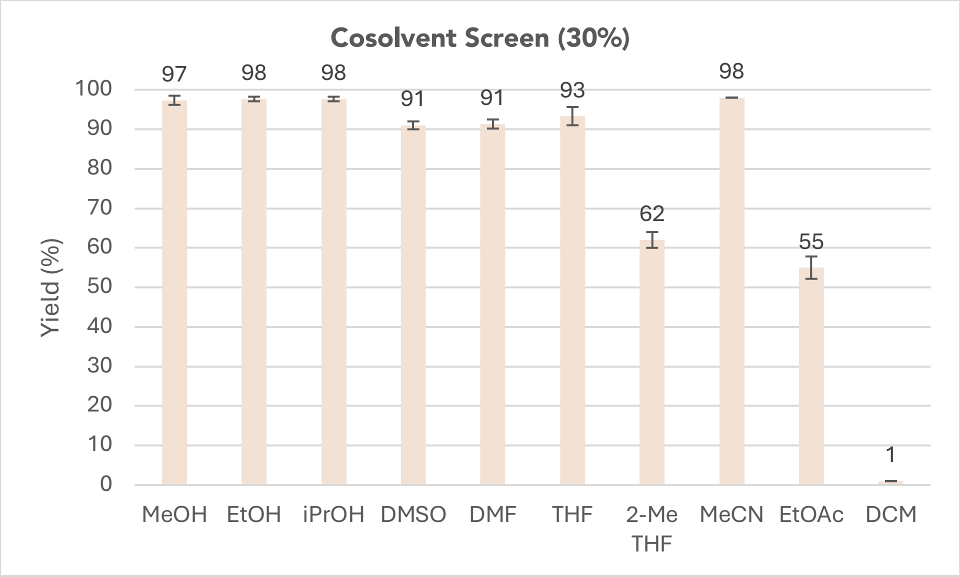

Figure S4. Cosolvent Screen

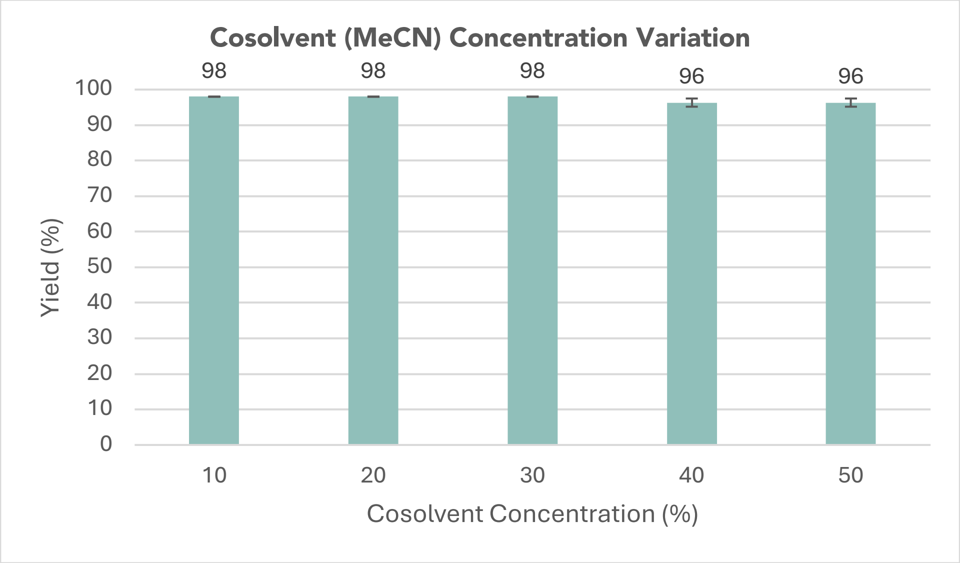

Figure S5. Cosolvent (MeCN) Loading Screen

References:

1. Wells, C. E.; Ramos, L. P. T.; Harstad, L. J.; Hessefort, L. Z.; Lee, H. J.; Sharma, M.; Biegasiewicz, K. F. Decarboxylative Bromooxidation of Indoles by a Vanadium Haloperoxidase. *ACS Catal.* **2023**, *13*, 4622-4628.
2. Zhao, G.; Wang, Y.; Wang, C.; Lei, H.; Yi, B.; Tong, R. Fenton-like chemistry enables catalytic oxidative desulfurization of thioacetals and thioketals with hydrogen peroxide. *Green Chem.* **2022**, *24*, 4041–4049.
3. Ali, M. H.; Goretti Gomes, M. A Simple and Efficient Heterogeneous Procedure for Thioacetalization of Aldehydes and Ketones. *Synthesis* **2005**, *8*, 1326–1332.
4. Aoyama, T.; Suzuki, T.; Nagaoka, T.; Takido, T.; Kodomari, M. Silica-Gel Supported Sulfamic Acid (SA/SiO_2) as an Efficient and Reusable Catalyst for Conversion of Ketones into Oxathioacetals and Dithioacetals. *Synth. Commun.* **2013**, *43*, 553–566.
5. Lamb, B. M.; Barbas III, C. F. Selective arylthiolane deprotection by singlet oxygen: a promising tool for sensors and prodrugs. *Chem. Commun.* **2015**, *51*, 3196–3199.
6. Yu, L.; Lai, G.-Q.; Zhang, P.; Li, Z.; Luh, T.-Y. Sequential olefination–dimerisation of benzylic dithioacetals by the nickel-catalysed reaction with methyl Grignard or zinc reagent. *RSC Adv.* **2022**, *12*, 9653–9659.
7. Hajipour, A. R.; Zarei, A.; Khazdooz, L.; Zahmatkesh, S.; Ruoho, A. E. A Mild and Chemoselective Catalyst for Thioacetalization Under Solvent Free Conditions. *Phosphorus, Sulfur, and Silicon and the Related Elements*, **2006**, *181*, 387–395.
8. Rudrawar, S.; Besra, R.; Chakraborti, A. Perchloric Acid Adsorbed on Silica Gel ($HClO_4$-SiO_2) as an Extremely Efficient and Reusable Catalyst for 1,3-Dithiolane/Dithiane Formation. *Synthesis* **2006**, *2006*, 2767–2771.
9. Saito, K.; Kondo, K.; Akiyama, T. $B(C_6F_5)_3$-Catalyzed Hydrodesulfurization Using Hydrosilanes – Metal-Free Reduction of Sulfides. *Org. Lett.* **2015**, *17*, 3366–3369.
10. Zhao, G.; Yuan, L.; Alami, M.; Provot, O. Chlorotrimethylsilane and Sodium Iodide: A Remarkable Metal-Free Association for the Desulfurization of Benzylic Dithioketals under Mild Conditions. *Adv. Synth. Catal.* **2018**, *360*, 2522–2536.
11. Ali, M. H.; Goretti Gomes, M. A Simple and Efficient Heterogeneous Procedure for Thioacetalization of Aldehydes and Ketones. *Synthesis* **2005**, *8*, 1326–1332.
12. Kamitori, Y.; Hojo, M.; Masuda, R.; Kimura, T.; Yoshida, T. (1986). Selective protection of carbonyl compounds. Silica gel treated with thionyl chloride as an effective catalyst for thioacetalization. *J. Org. Chem.* **1986**, *51*, 1427–1431.
13. Dong, K.; Li, J.; Li, R.-P.; Mao, M.; Liu, J.; Wang, X.; Tang, S. One-Pot Sequential Synthesis of 3,3′- or 2,3′-Bis(indolyl)methanes by Using 1,3-Dithiane as the Methylene Source *J. Org. Chem.* **2022**, *87*, 14930–14939.
14. Xu, C.; Du, W.; Zeng, Y.; Dai, B.; Guo, H. Reactivity Switch Enabled by Counterion: Highly Chemoselective Dimerization and Hydration of Terminal Alkynes. *Org. Lett.* **2014**, *16*, 948–951.

15. Moriyama, K.; Takemura, M.; Togo, H. Direct and Selective Benzylic Oxidation of Alkylarenes via C–H Abstraction Using Alkali Metal Bromides. *Org. Lett.* **2012**, *14*, 2414–2417.
16. Moorthy, J. N.; Senapati, K.; Parida, K. N.; Jhulki, S.; Sooraj, K.; Nair, N. N. *Twist* Does a *Twist* to the Reactivity: Stoichiometric and Catalytic Oxidations with *Twisted* Tetramethyl-IBX. *J. Org. Chem.* **2011**, *76*, 9593–9601.
17. Yang, X.; Guo, Y.; Tong, H.; Guo, H.; Liu, R.; Zhou, R. Photochemical Hydrogen Atom Transfer Catalysis for Dehydrogenation of Alcohols to Form Carbonyls. *Org. Lett.* **2023**, *25*, 5486–5491.
18. Pramanik, C.; Bapat, K.; Chaudhari, A.; Tripathy, N. K.; Gurjar, M. K. A New Solvent System (Cyclopentyl Methyl Ether–Water) in Process Development of Darifenacin HBr. *Org. Process Res. Dev*. **2012**, *16*, 1591–1597.
19. Konwar, M.; Das, A. Cyclometalated Ruthenium-Complex-Catalyzed Selective Oxidation of Olefins to Carbonyls. *Org. Lett.* **2024**, *26*, 10235–10240.
20. Liu, X.; Xia, Q.; Zhang, Y.; Chen, C.; Chen, W. Cu-NHC-TEMPO Catalyzed Aerobic Oxidation of Primary Alcohols to Aldehydes. *J. Org. Chem.* **2013**, *78*, 8531–8536.
21. Gopinath, R.; Haque, Sk. J.; Patel, B. K. Tetrabutylammonium Tribromide (TBATB) as An Efficient Generator of HBr for an Efficient Chemoselective Reagent for Acetalization of Carbonyl Compounds. *J. Org. Chem.* **2002**, *67*, 5842–5845.
22. Chauhan, S. M. S.; Kumar, A.; Sahoo, B. B. Deprotection of 1,3-Oxathiolanes to Carbonyl Compounds with Montmorillonite K10. *Indian J. Chem. Sect. B* **2004**, *42B,* 2635-2637.

Spectroscopic Data

2-Methyl-2-phenyl-1,3-dithiolane (1)

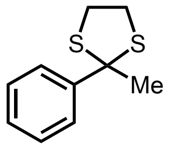

^{1}H-NMR
400 MHz
$CDCl_3$

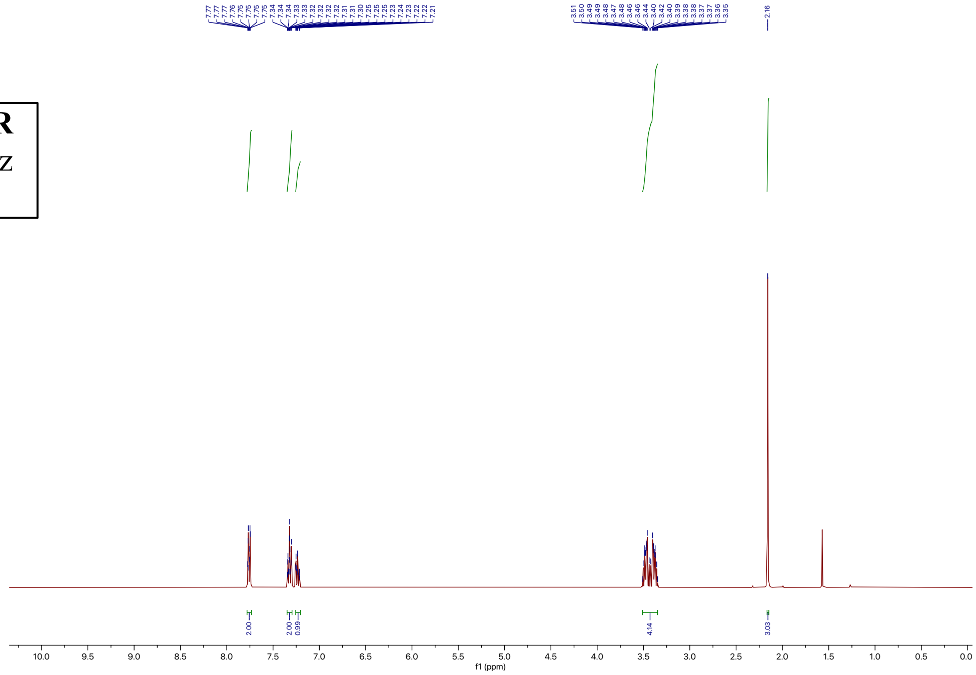

^{13}C-NMR
101 MHz
$CDCl_3$

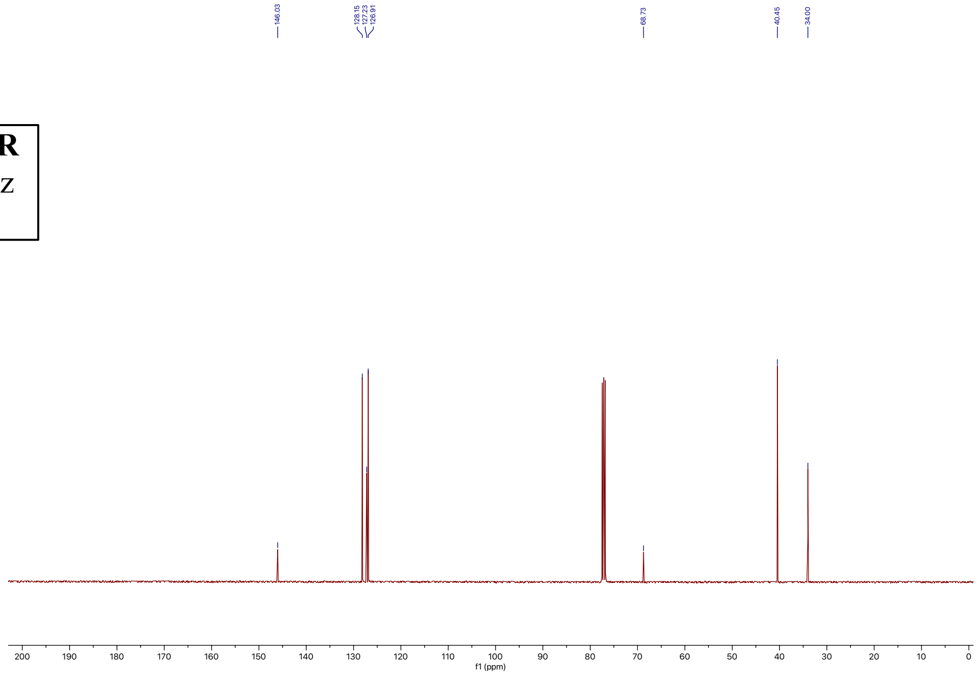

2-(4-Bromophenyl)-2-methyl-1,3-dithiolane (SM-2)

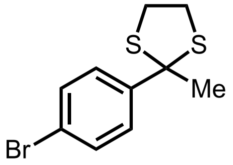

^{1}H-NMR
400 MHz
$CDCl_3$

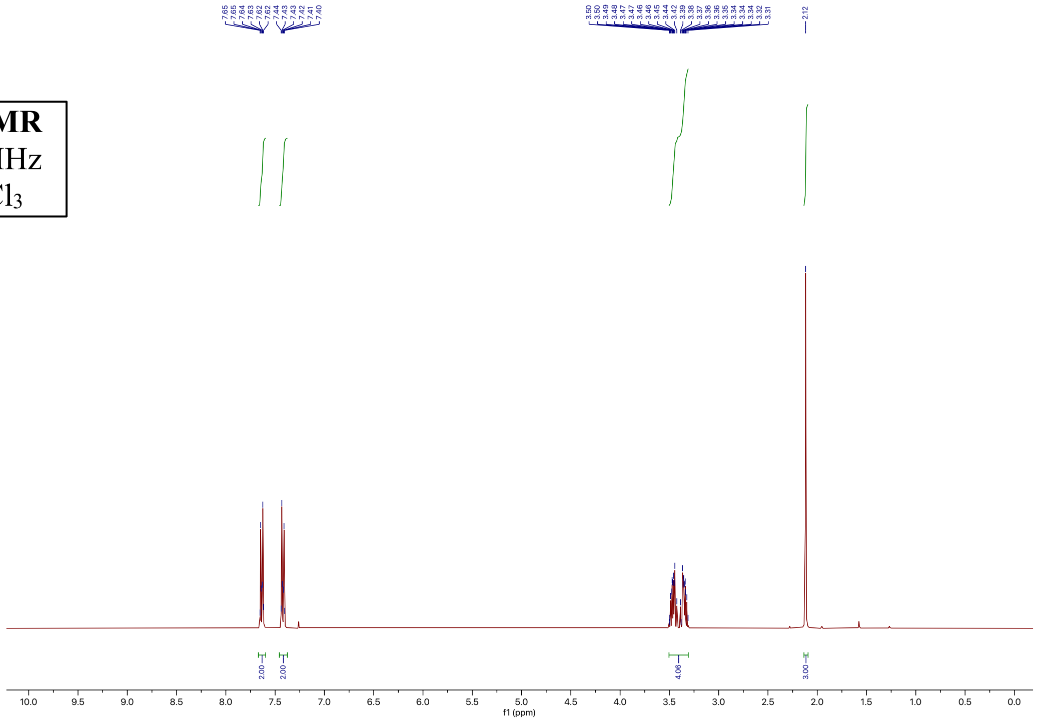

^{13}C-NMR
101 MHz
$CDCl_3$

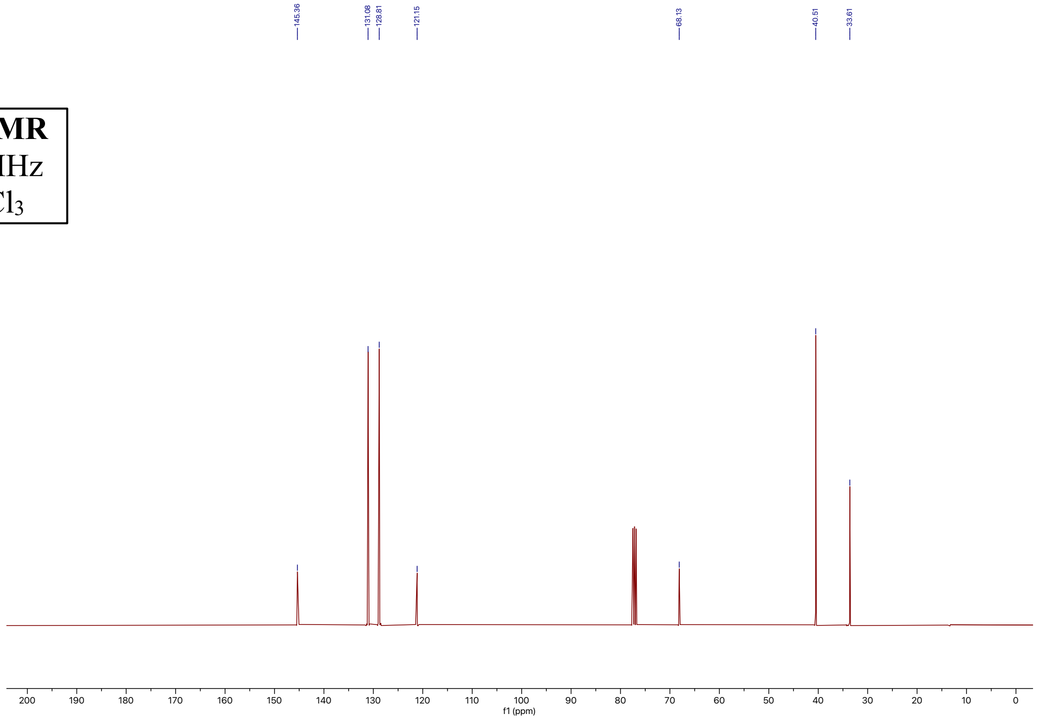

2-(4-Clorophenyl)-2-methyl-1,3-dithiolane (SM-3)

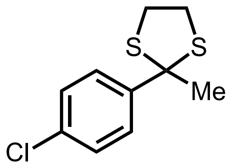

^{1}H-NMR
400 MHz
$CDCl_3$

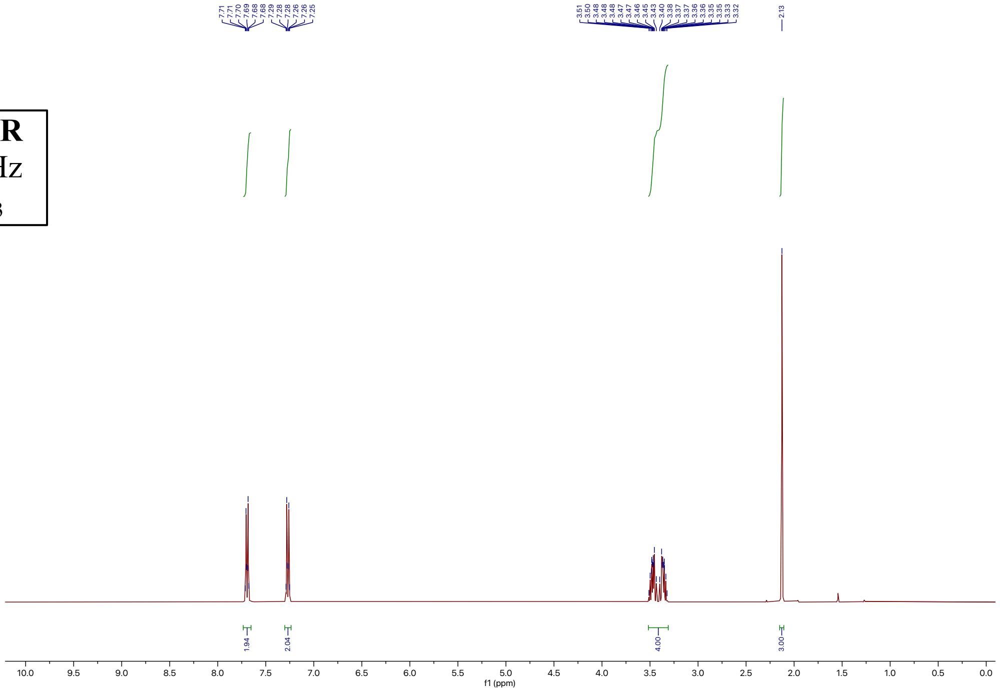

^{13}C-NMR
101 MHz
$CDCl_3$

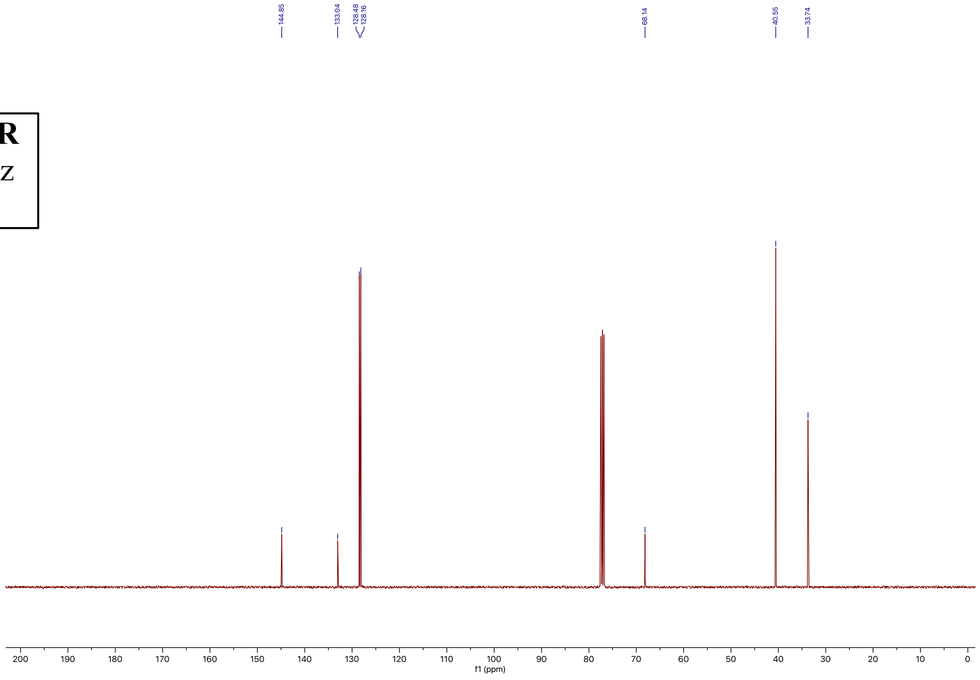

2-(4-Fluorophenyl)-2-methyl-1,3-dithiolane (SM-4)

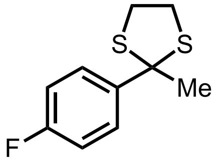

^{1}H-NMR
400 MHz
$CDCl_3$

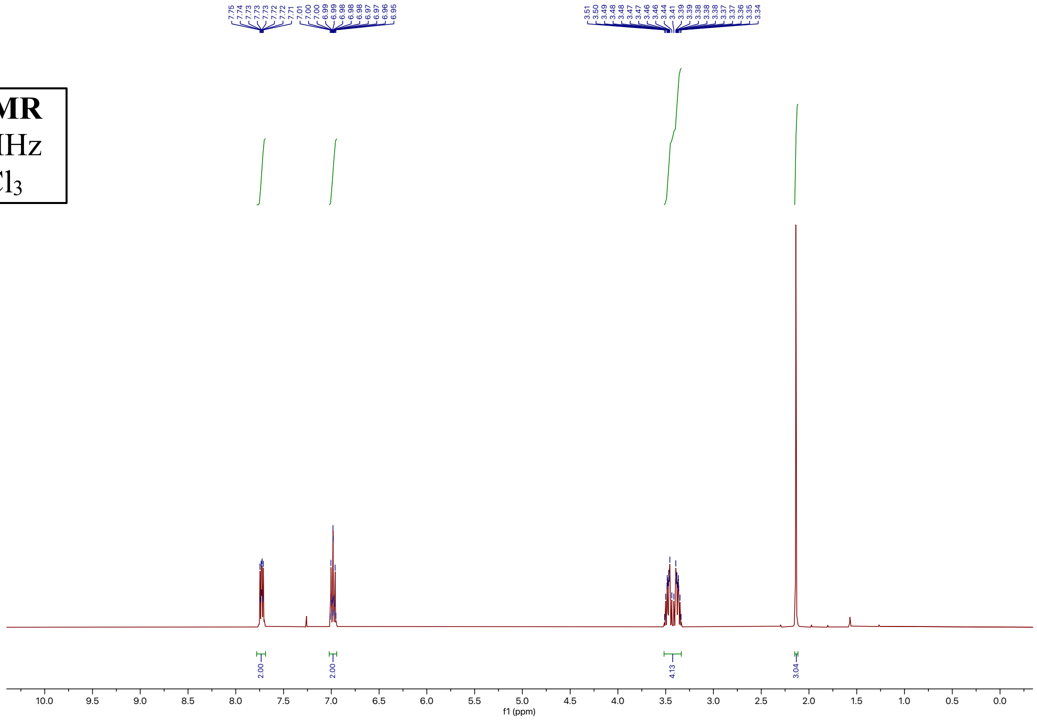

^{13}C-NMR
101 MHz
$CDCl_3$

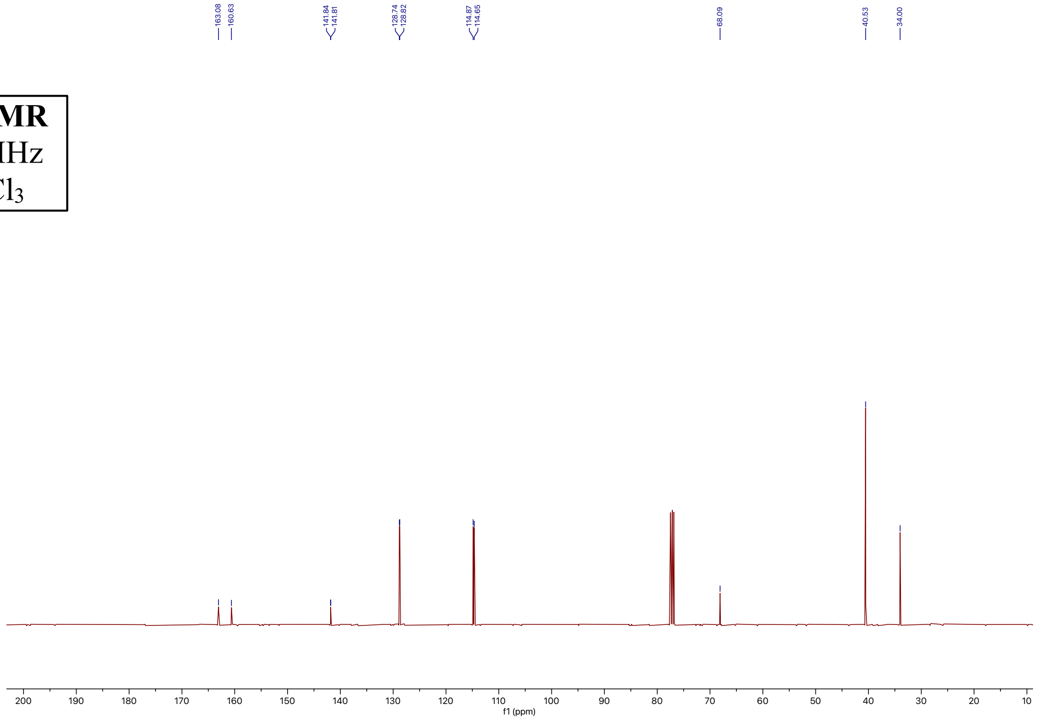

2-(4-Methoxyphenyl)-2-methyl-1,3-dithiolane (SM-5)

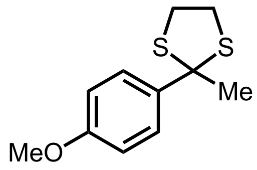

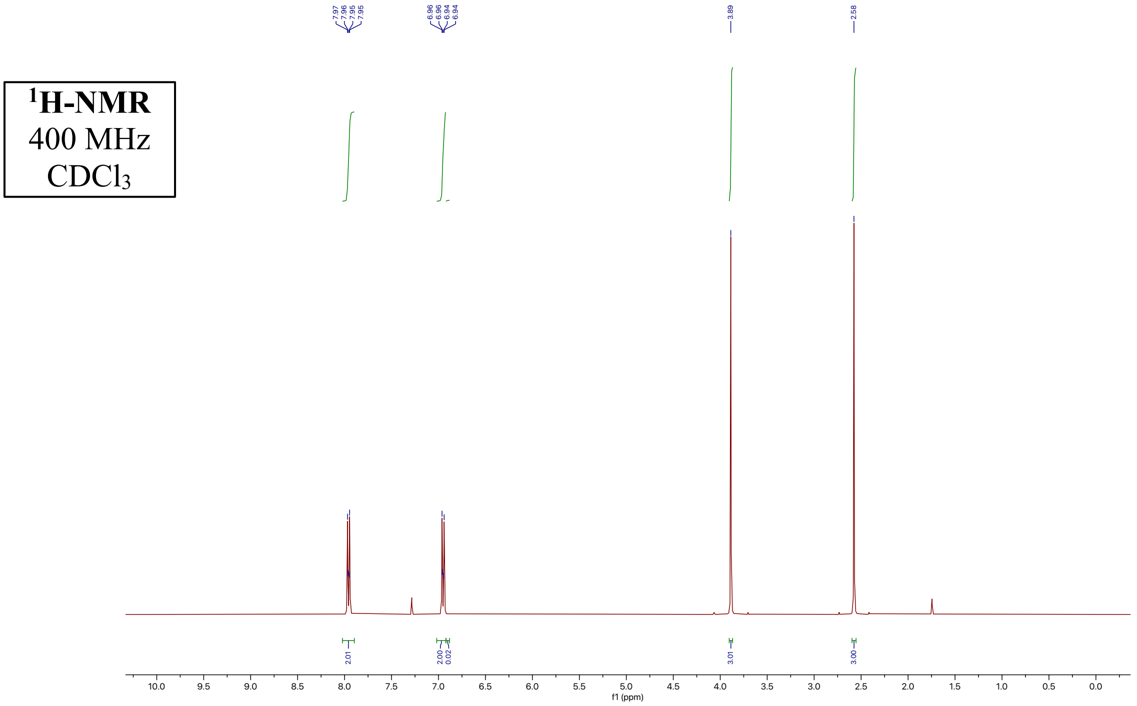

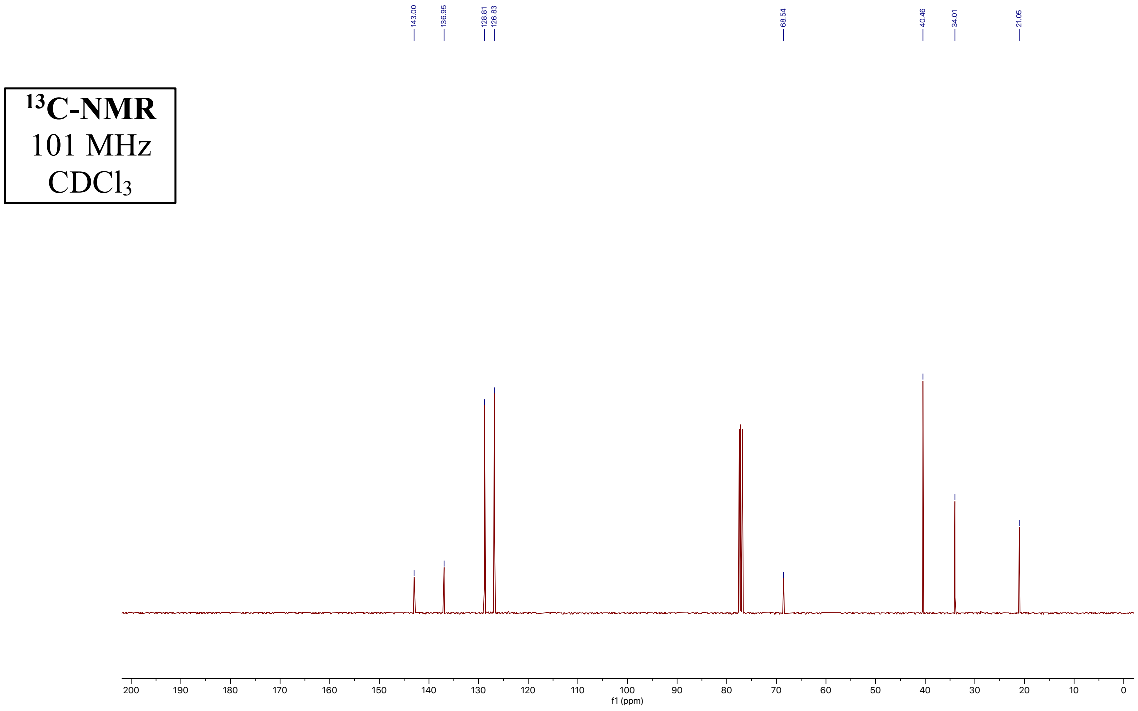

2-Methyl-2-(p-tolyl)-1,3-dithiolane (SM-6)

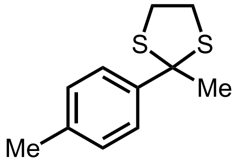

^{1}H-NMR
400 MHz
$CDCl_3$

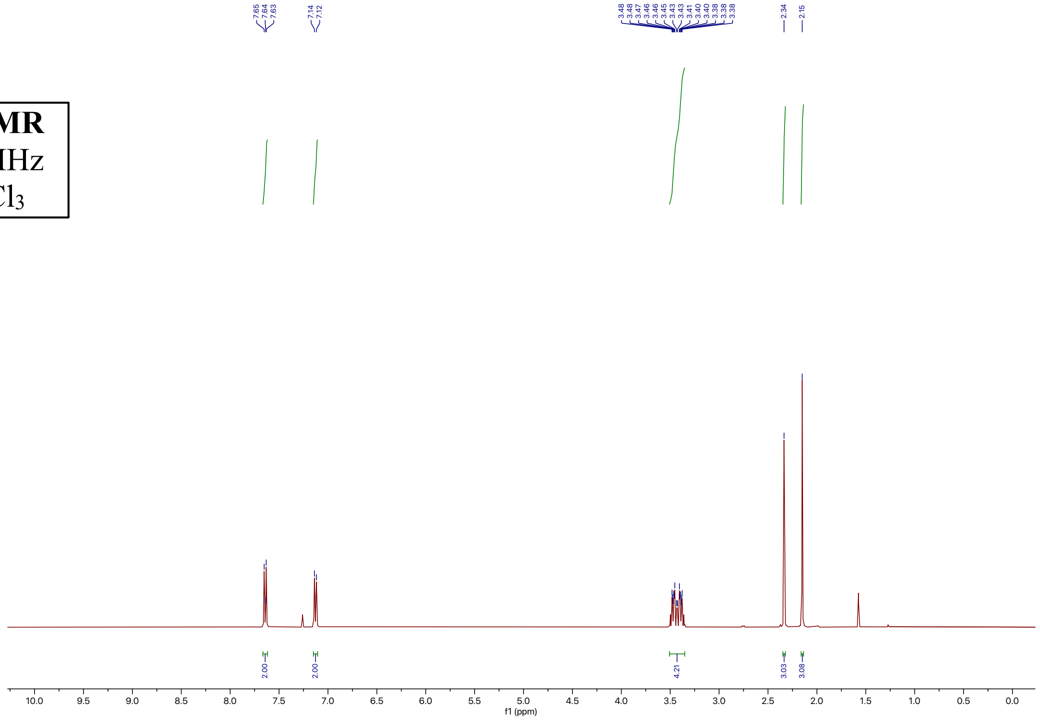

^{13}C-NMR
101 MHz
$CDCl_3$

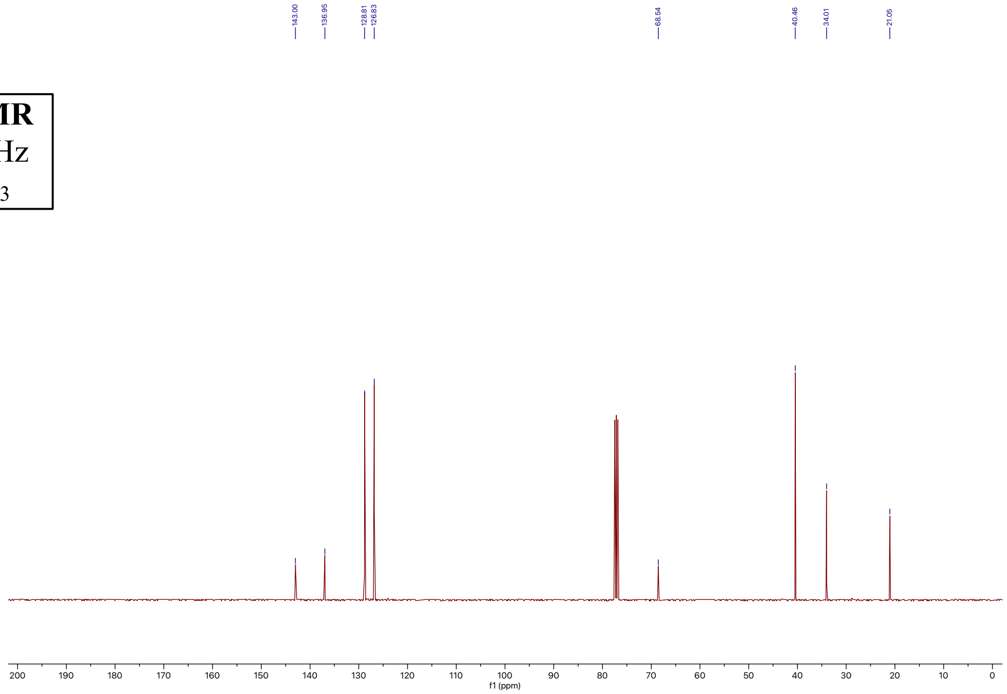

2-(4-(tert-Butyl)phenyl)-2-methyl-1,3-dithiolane (SM-7)

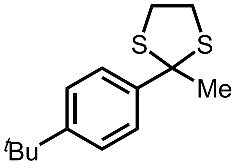

^{1}H-NMR
400 MHz
$CDCl_3$

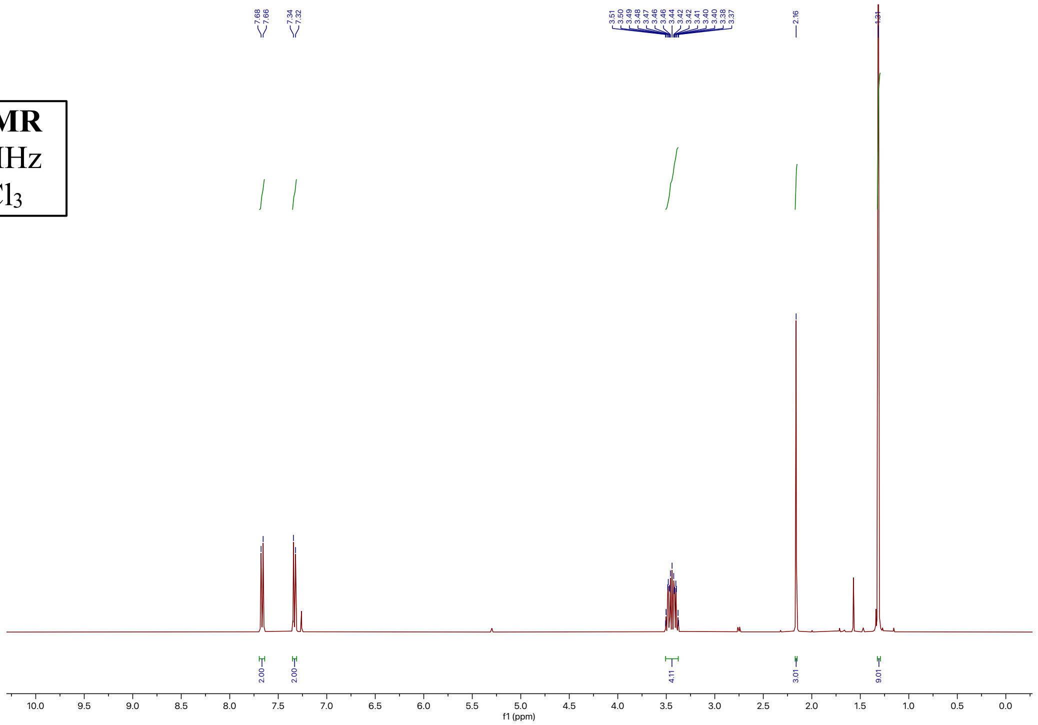

^{13}C-NMR
101 MHz
$CDCl_3$

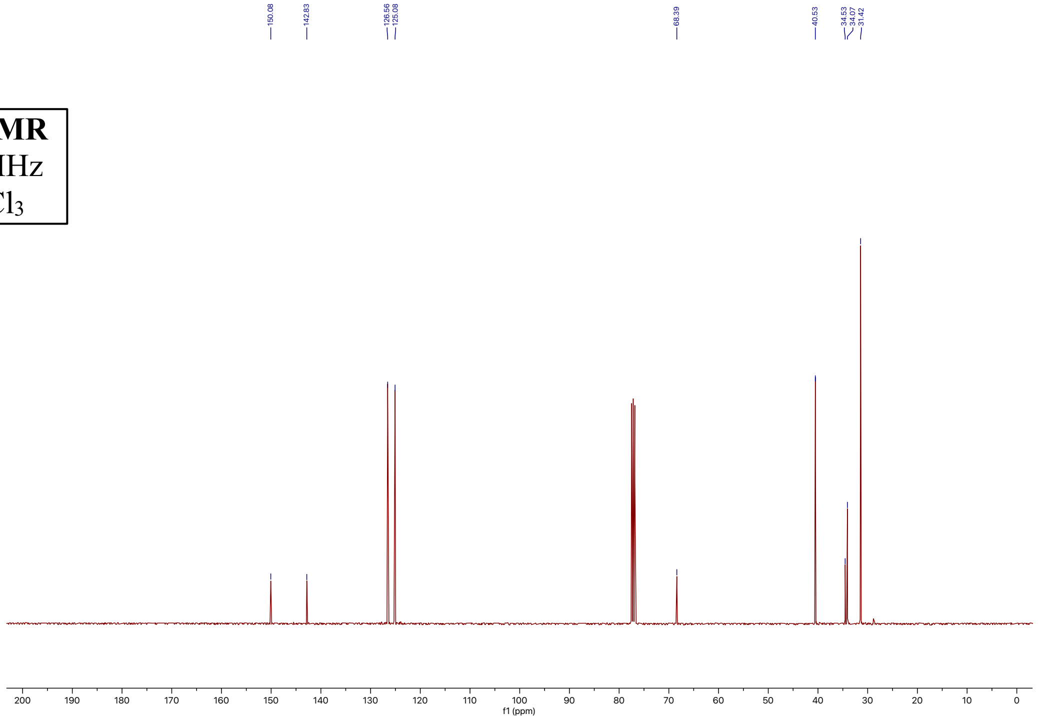

2-Methyl-2-(4-nitrophenyl)-1,3-dithiolane (SM-8)

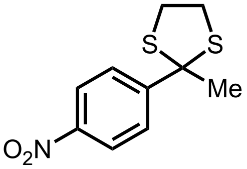

^{1}H-NMR
400 MHz
$CDCl_3$

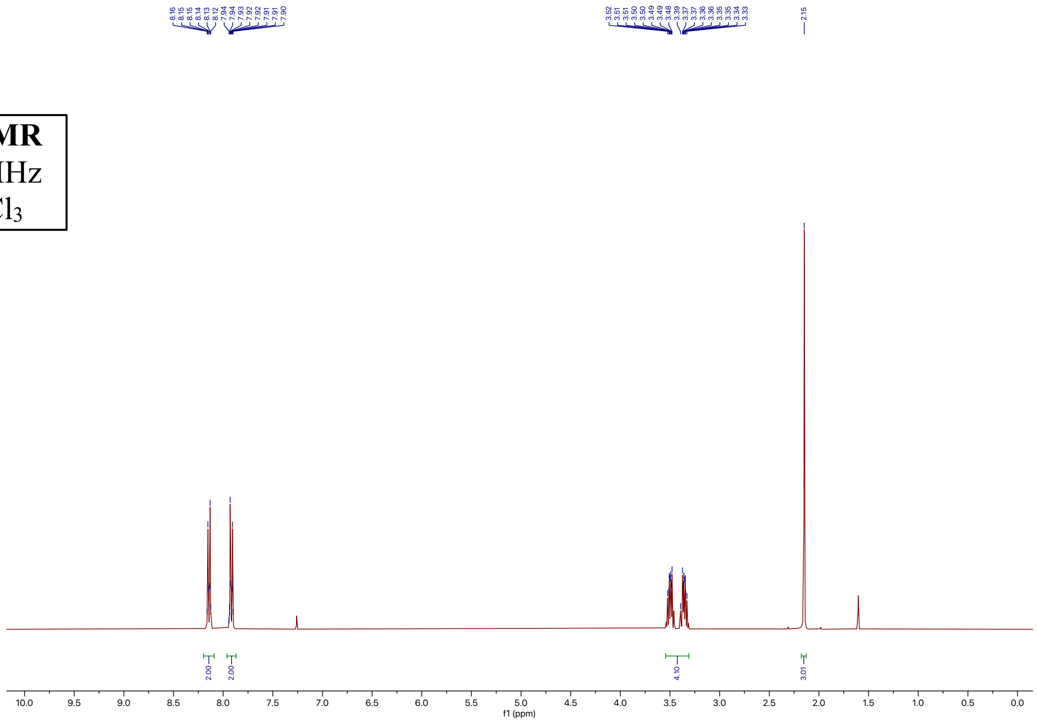

^{13}C-NMR
101 MHz
$CDCl_3$

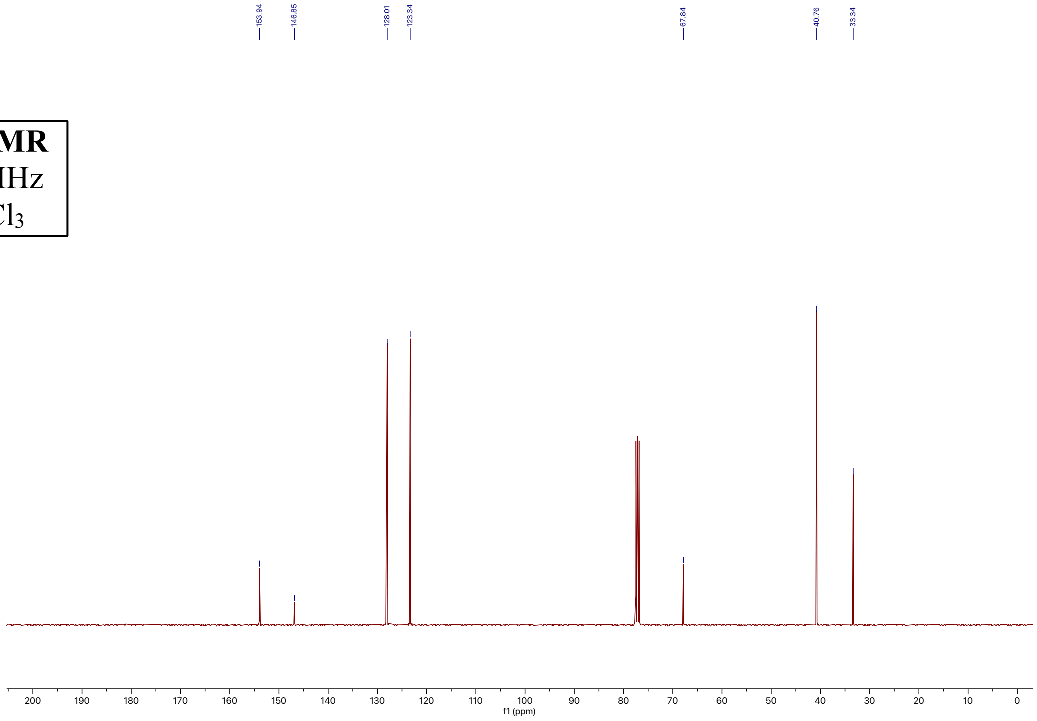

2-([1,1'-Biphenyl]-4-yl)-2-methyl-1,3-dithiolane (SM-9)

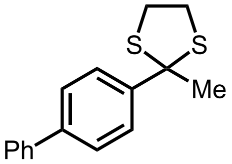

^{1}H-NMR
400 MHz
$CDCl_3$

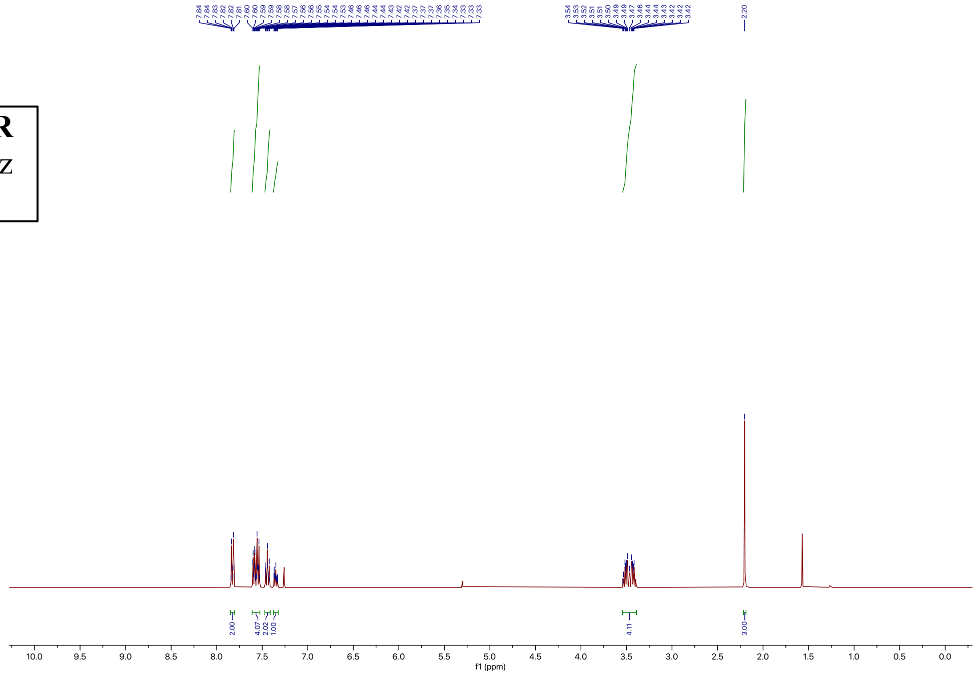

^{13}C-NMR
101 MHz
$CDCl_3$

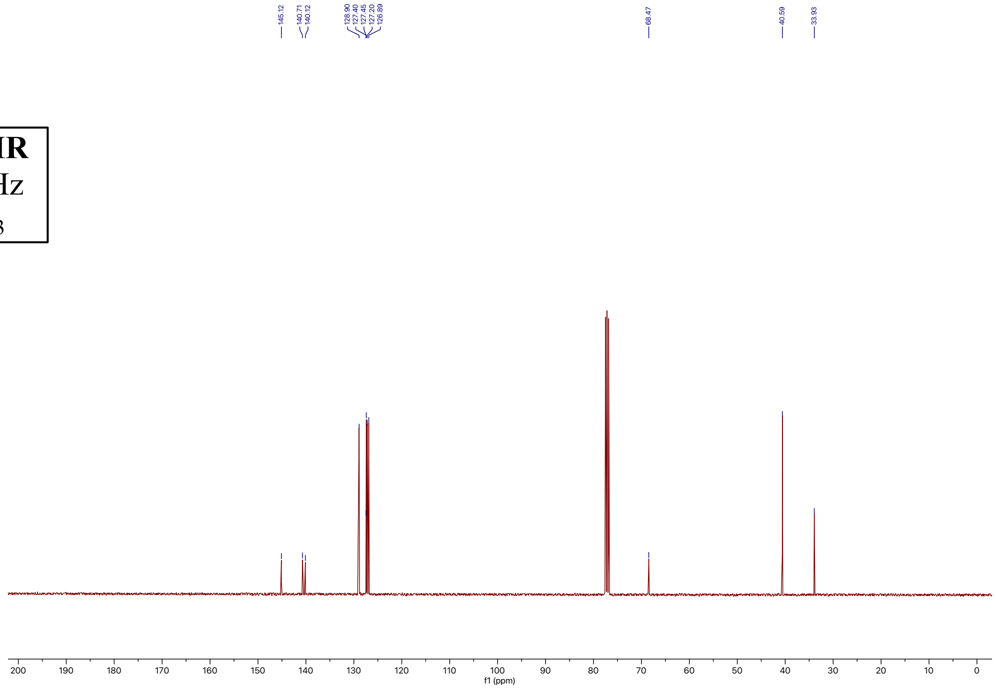

Methyl 4-(2-methyl-1,3-dithiolan-2-yl)benzoate (SM-10)

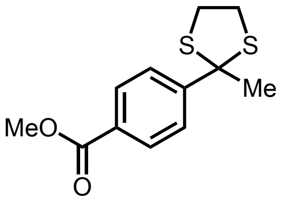

^{1}H-NMR
400 MHz
$CDCl_3$

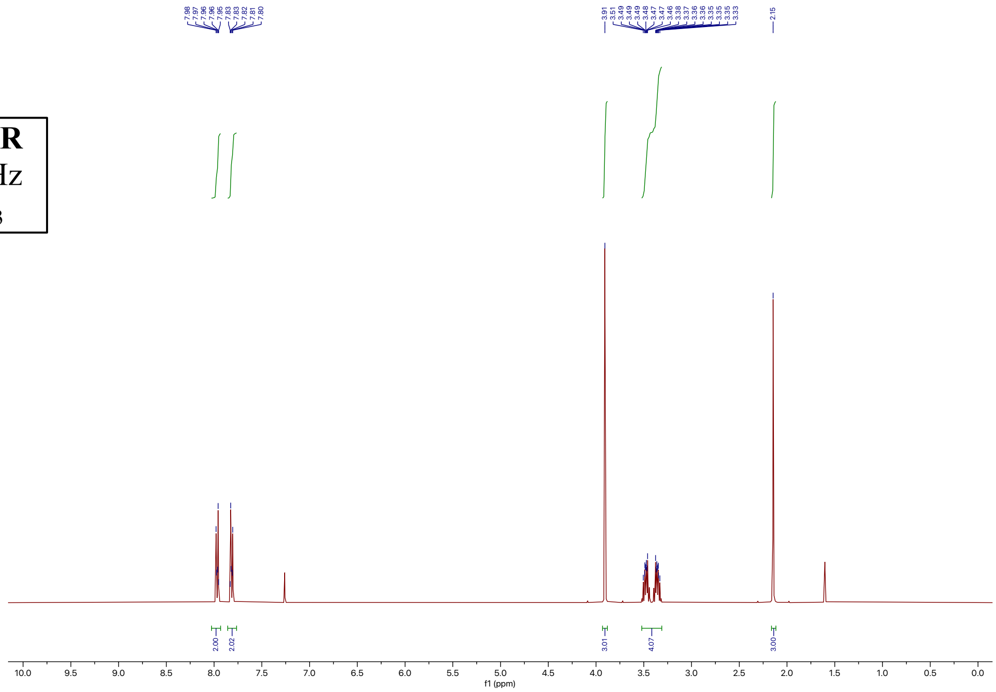

^{13}C-NMR
101 MHz
$CDCl_3$

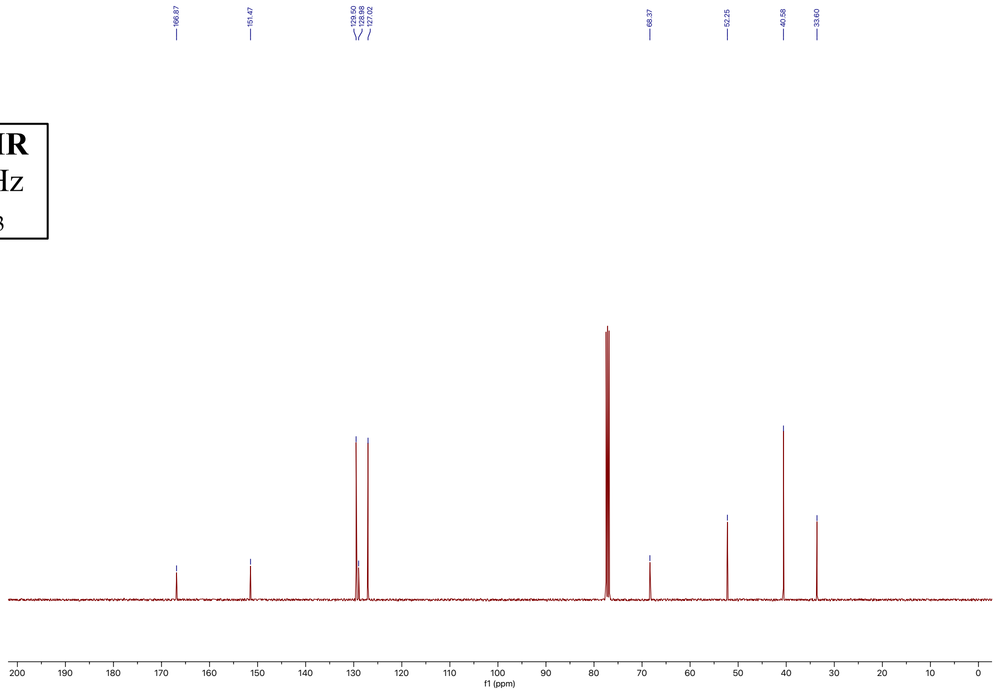

2-(3-Methoxyphenyl)-2-methyl-1,3-dithiolane (SM-11)

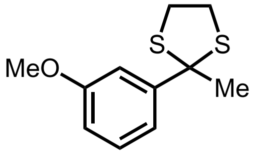

^{1}H-NMR
400 MHz
$CDCl_3$

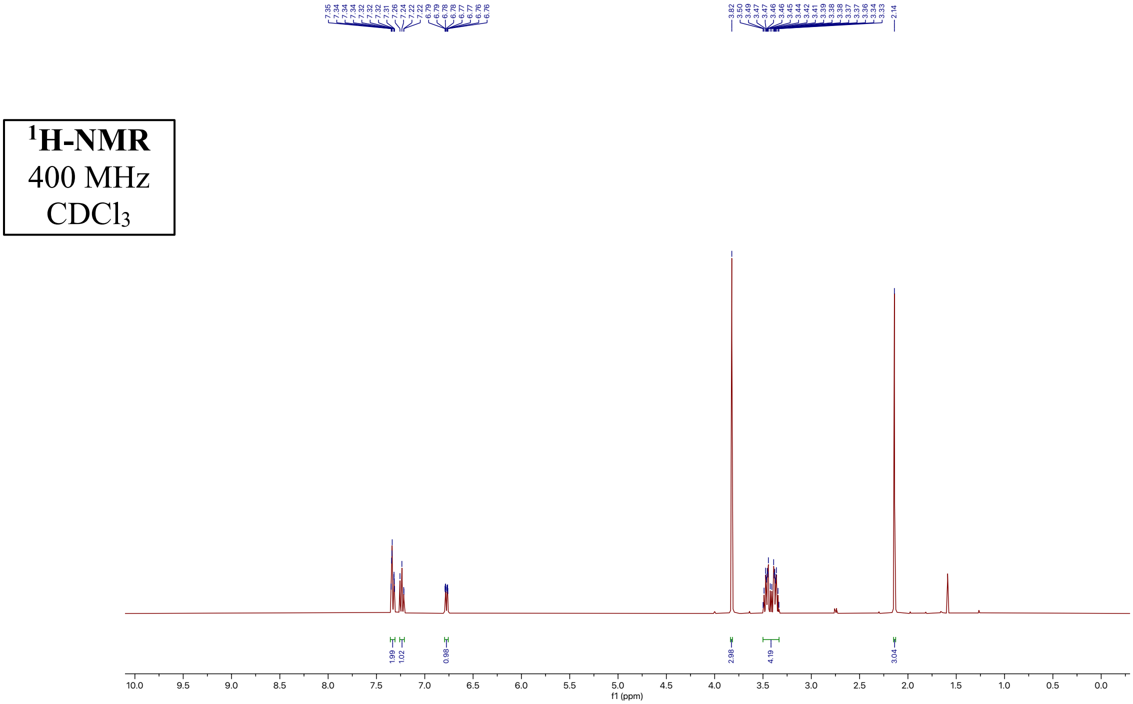

^{13}C-NMR
101 MHz
$CDCl_3$

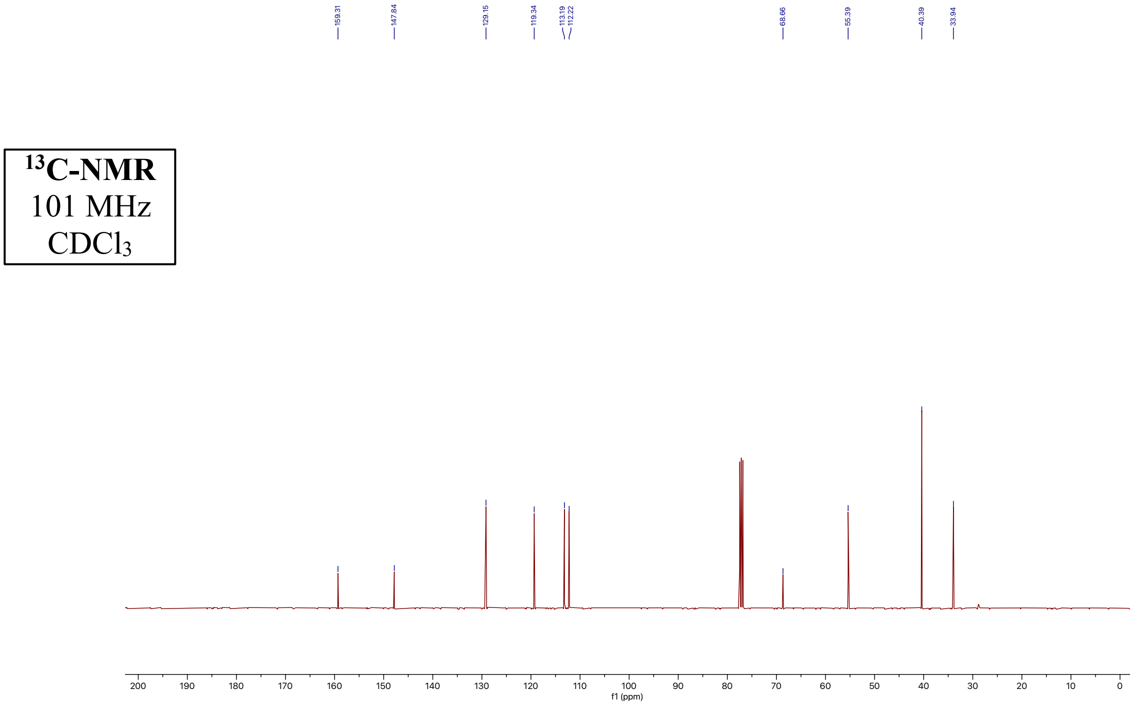

2-Methyl-2-(m-tolyl)-1,3-dithiolane (SM-12)

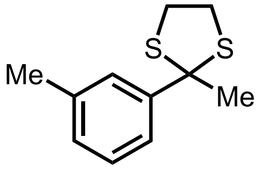

^{1}H-NMR
400 MHz
$CDCl_3$

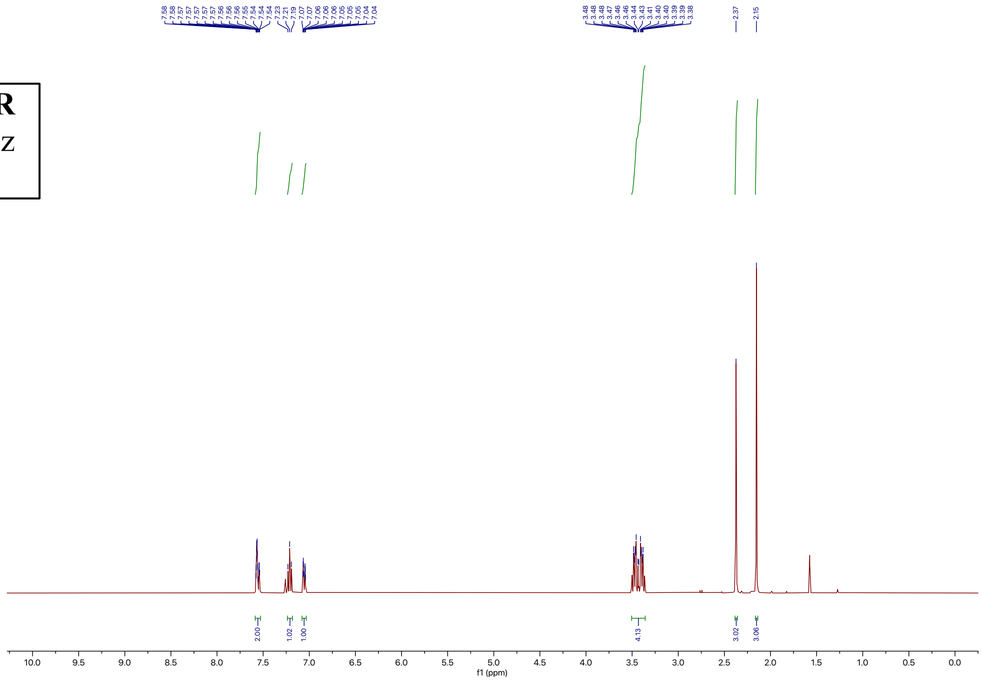

^{13}C-NMR
101 MHz
$CDCl_3$

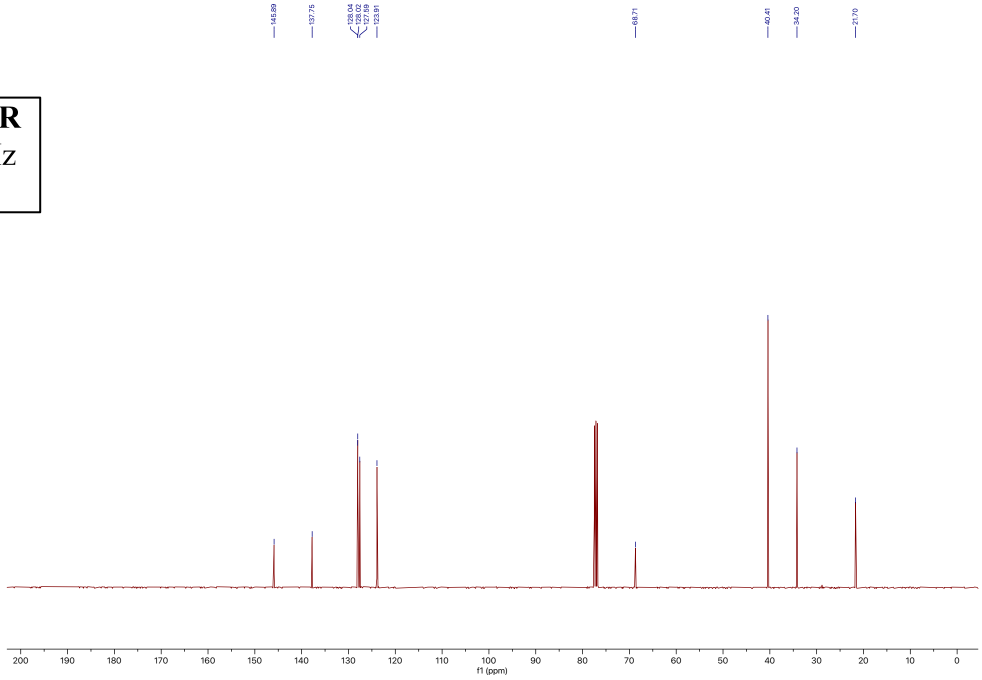

2-Methyl-2-(o-tolyl)-1,3-dithiolane (SM-13)

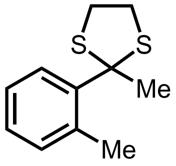

^{1}H-NMR
400 MHz
$CDCl_3$

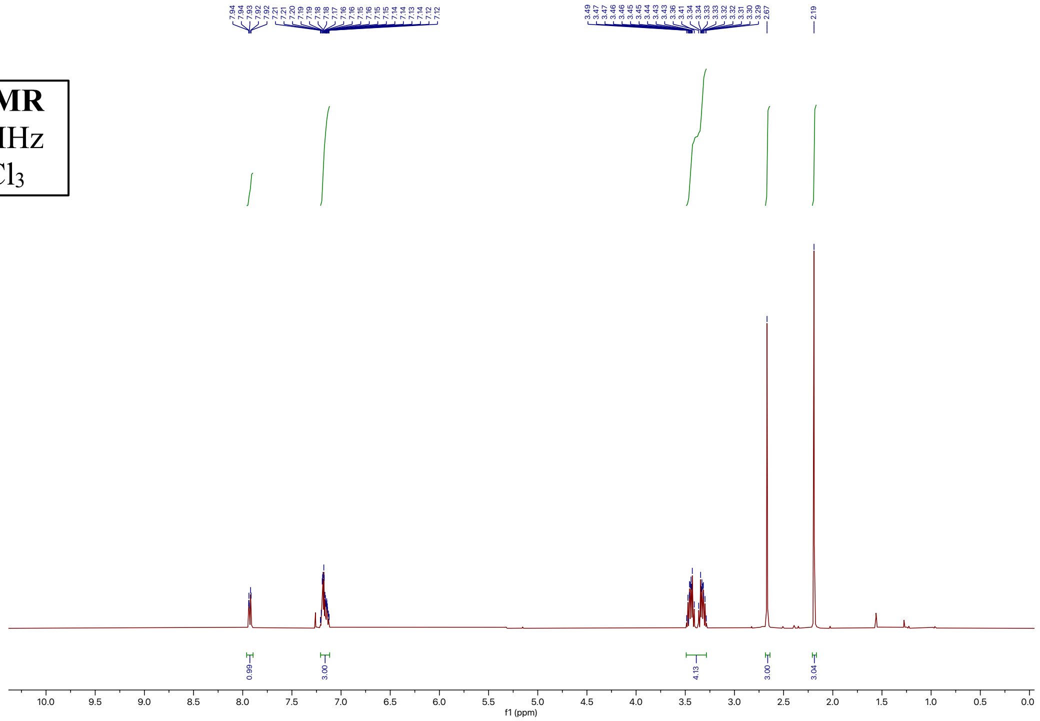

^{13}C-NMR
101 MHz
$CDCl_3$

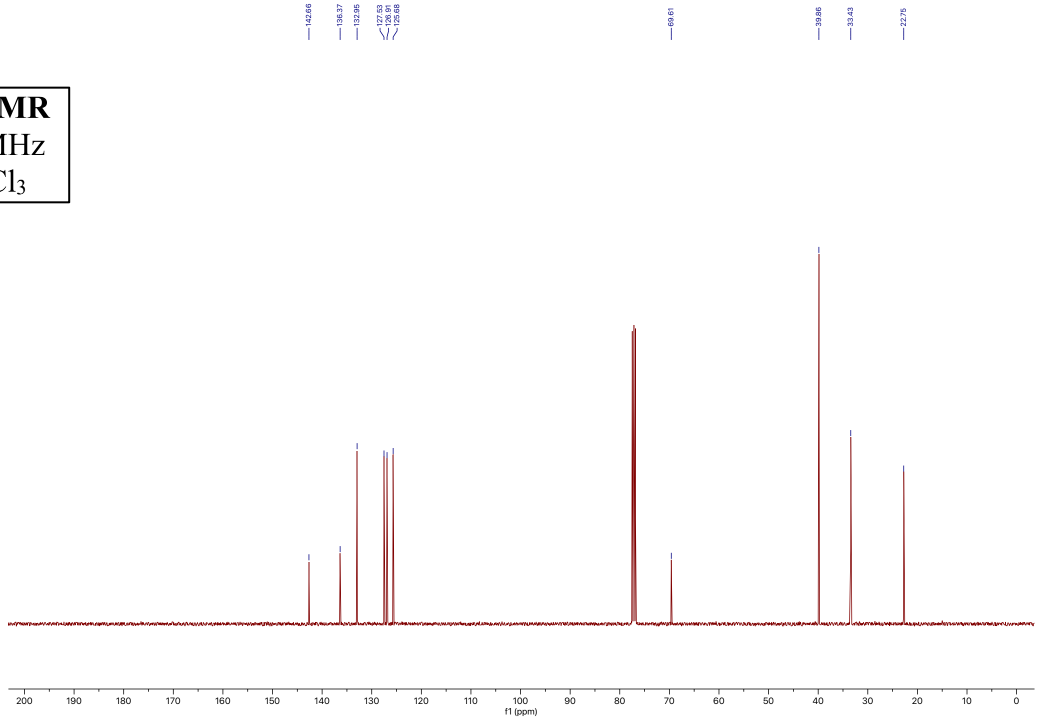

2-(2-Methoxyphenyl)-2-methyl-1,3-dithiolane (SM-14)

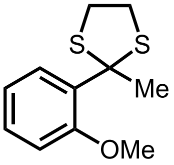

^{1}H-NMR
400 MHz
$CDCl_3$

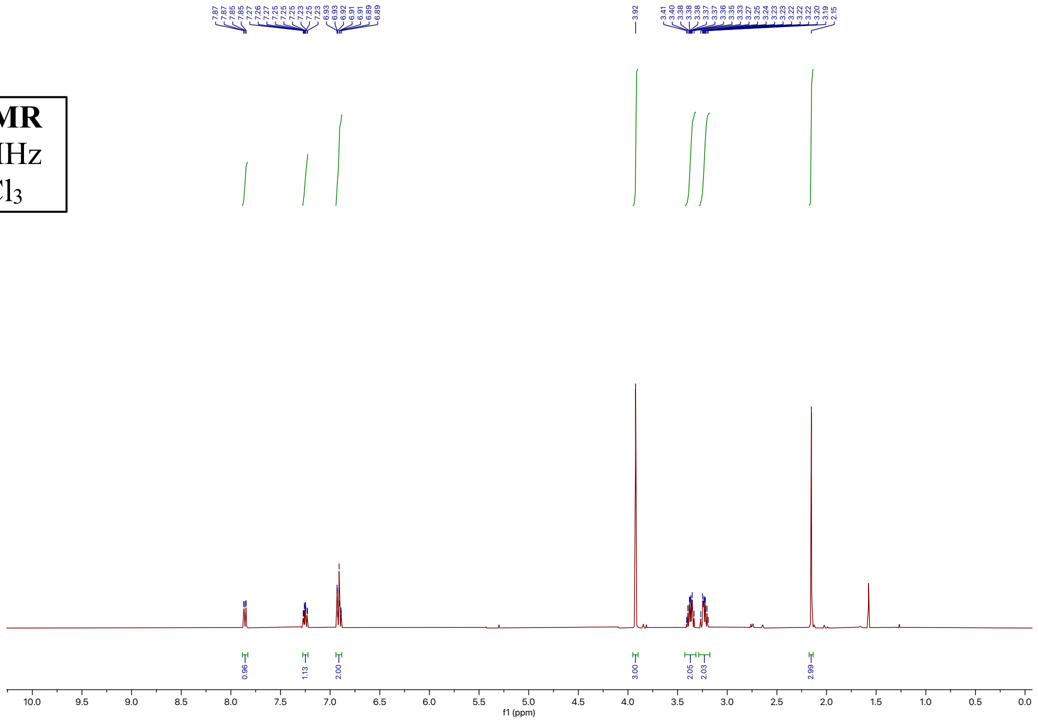

^{13}C-NMR
101 MHz
$CDCl_3$

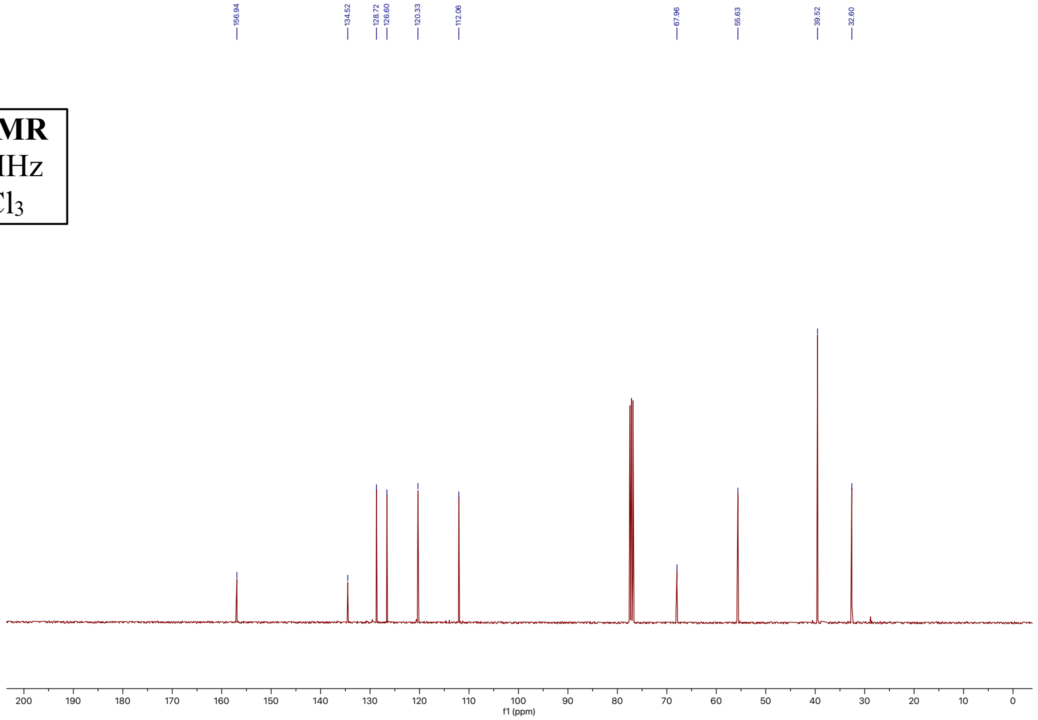

2-Methyl-2-(thiophen-2-yl)-1,3-dithiolane (SM-15)

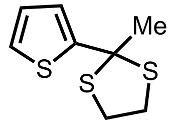

^{1}H-NMR
400 MHz
$CDCl_3$

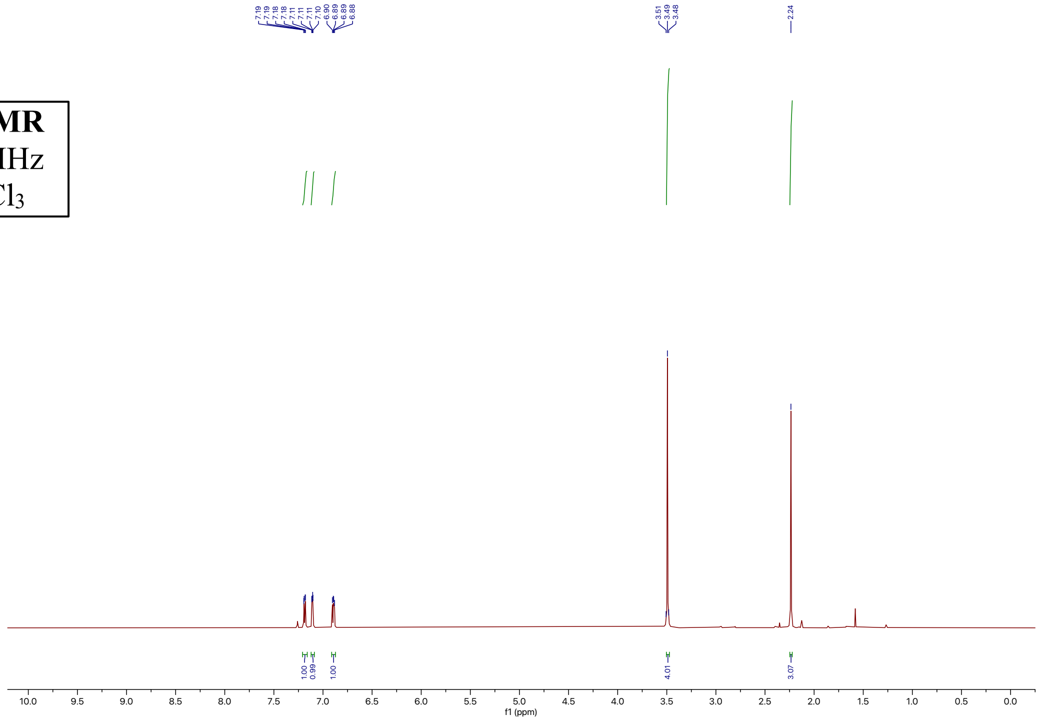

^{13}C-NMR
101 MHz
$CDCl_3$

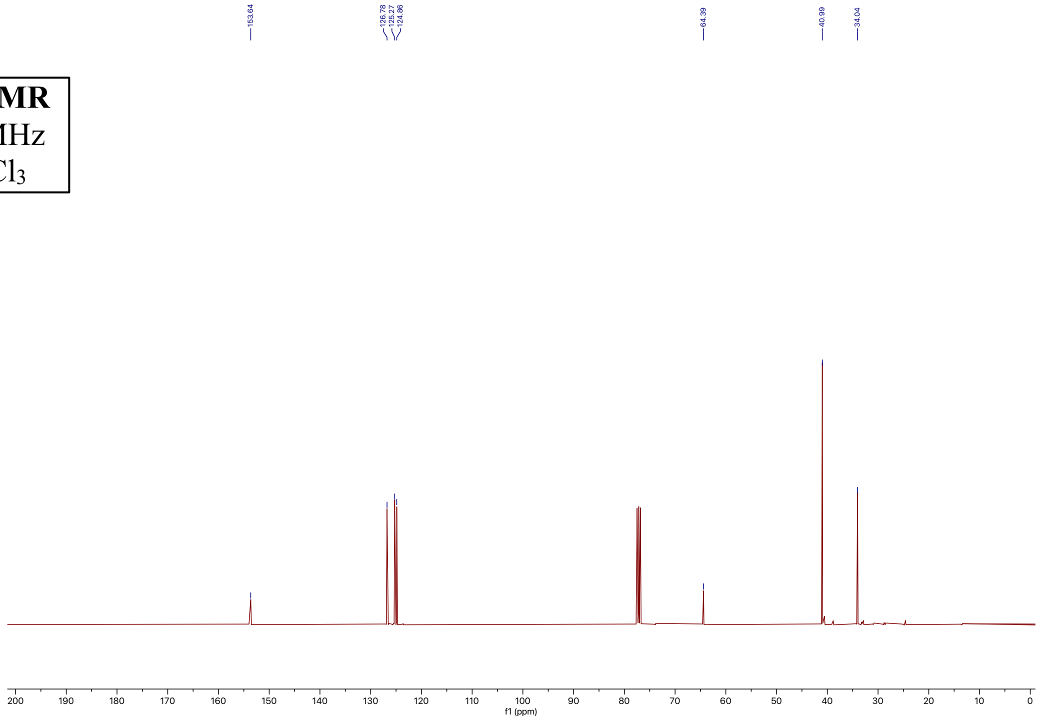

2-Phenyl-2-(thiophen-2-yl)-1,3-dithiolane (SM-16)

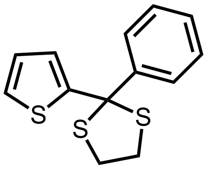

^{1}H-NMR
400 MHz
$CDCl_3$

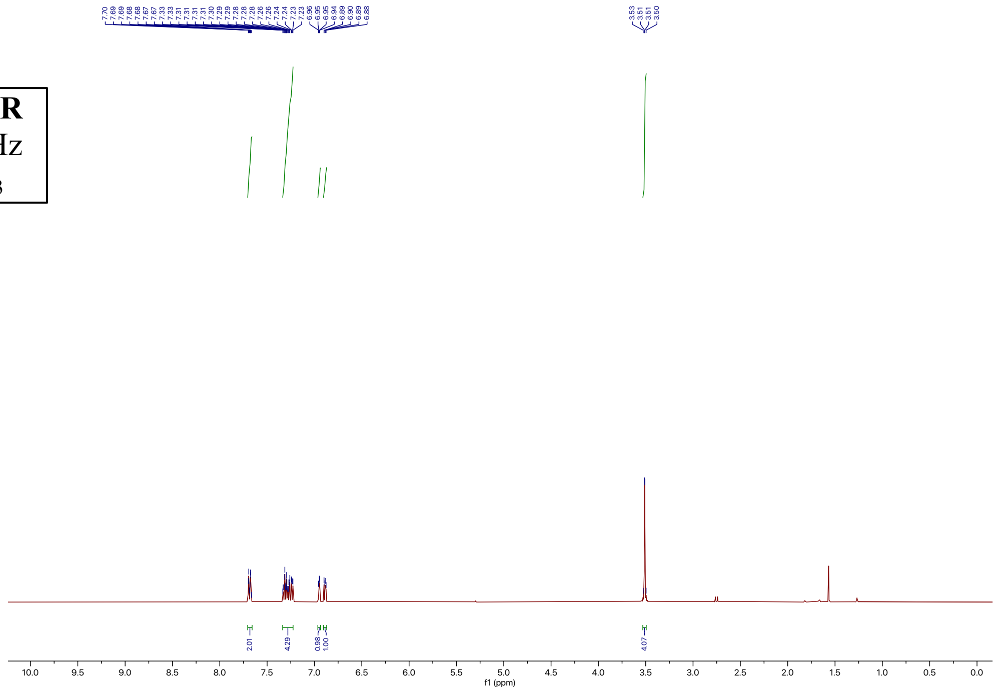

^{13}C-NMR
101 MHz
$CDCl_3$

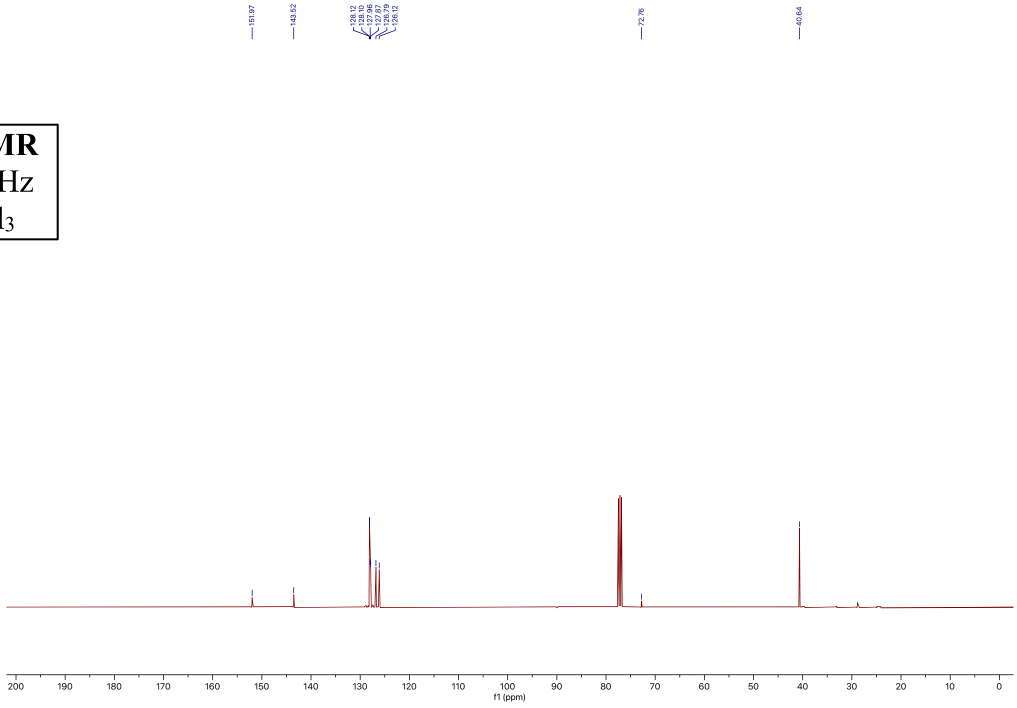

2-Methyl-2-(naphthalen-2-yl)-1,3-dithiolane (SM-17)

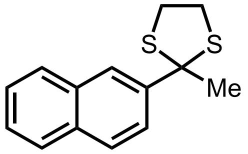

^{1}H-NMR
400 MHz
$CDCl_3$

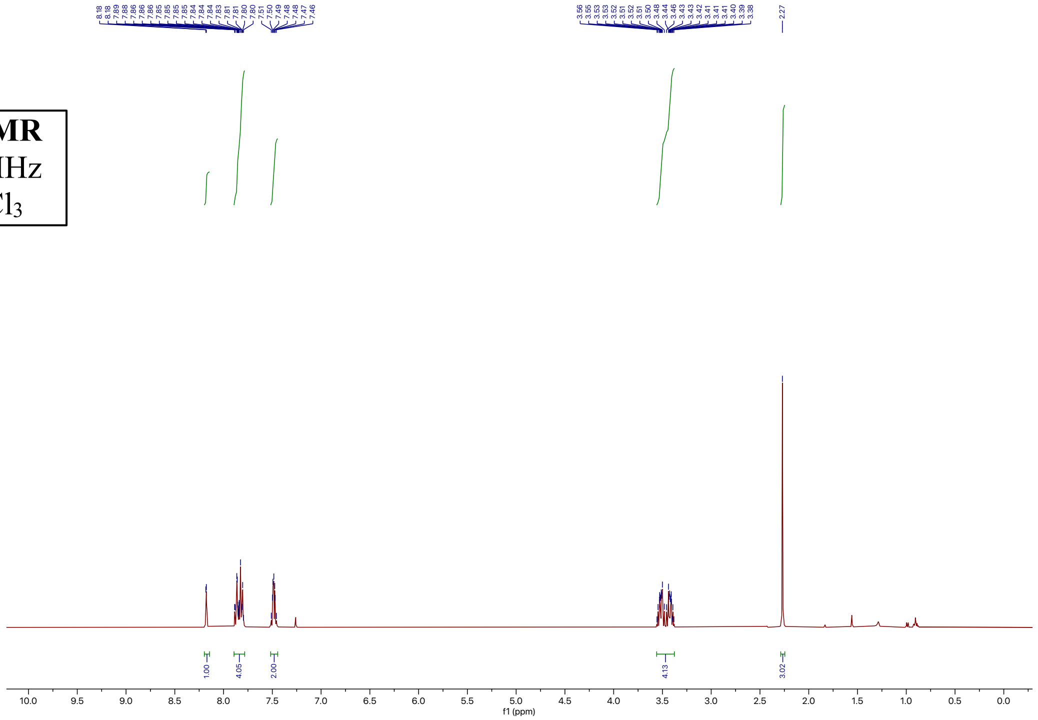

^{13}C-NMR
101 MHz
$CDCl_3$

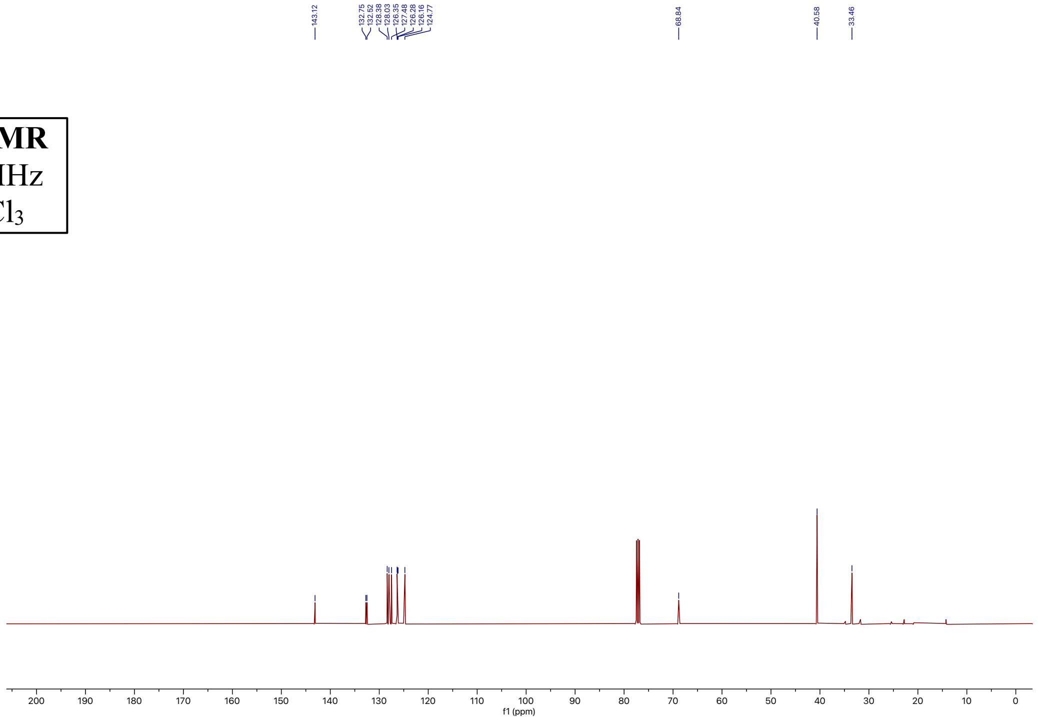

5-(2-Methyl-1,3-dithiolan-2-yl)-2,3-dihydrobenzofuran (SM-18)

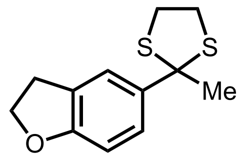

^{1}H-NMR
400 MHz
$CDCl_3$

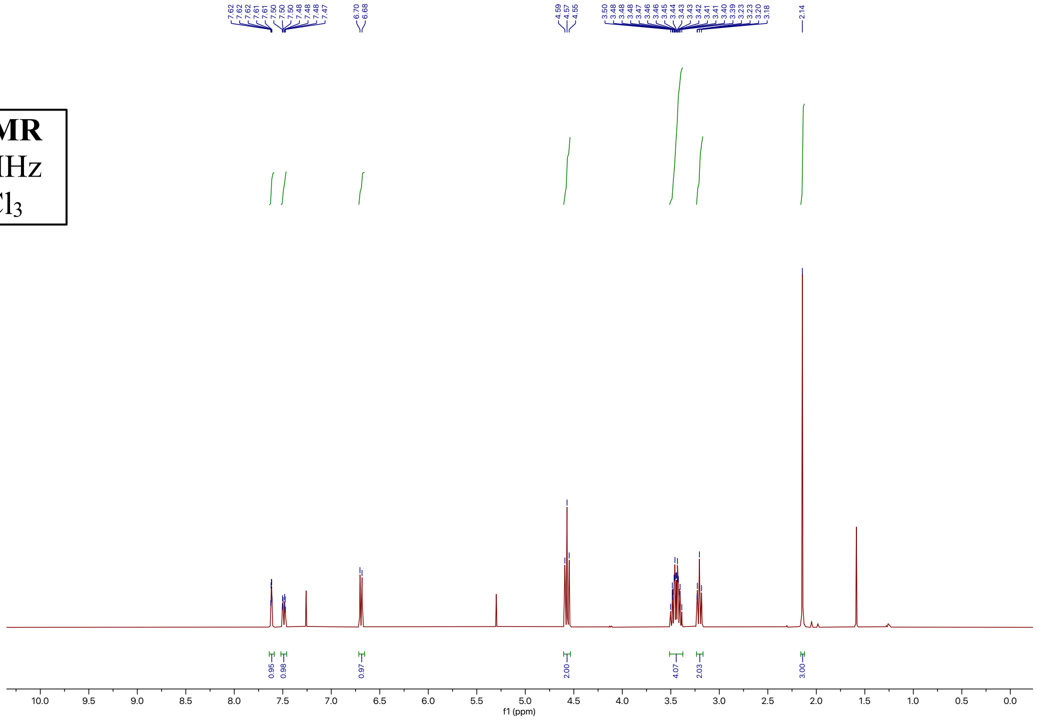

^{13}C-NMR
101 MHz
$CDCl_3$

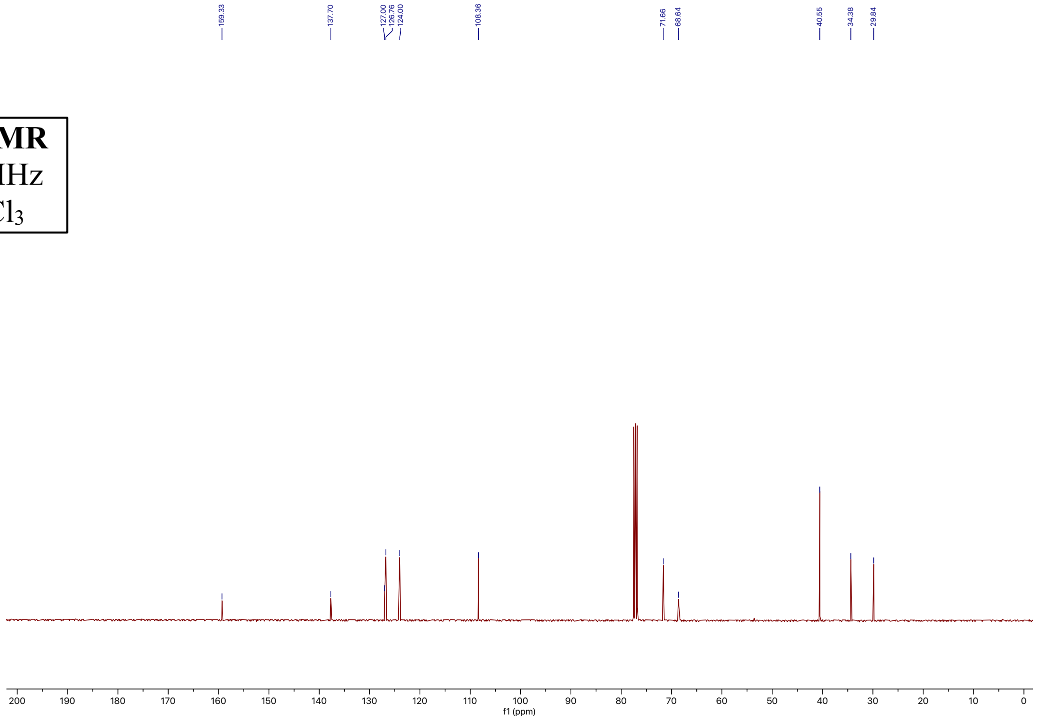

3,4-Dihydro-2H-spiro[naphthalene-1,2'-[1,3]dithiolane] (SM-19)

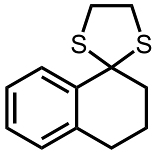

^{1}H-NMR
400 MHz
$CDCl_3$

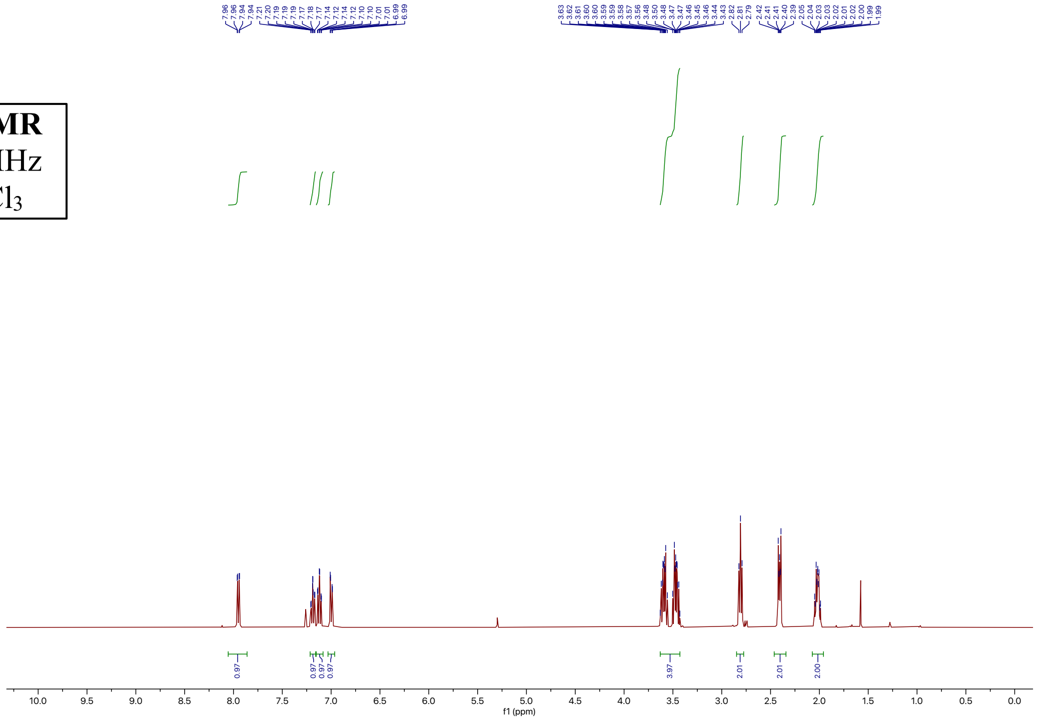

^{13}C-NMR
101 MHz
$CDCl_3$

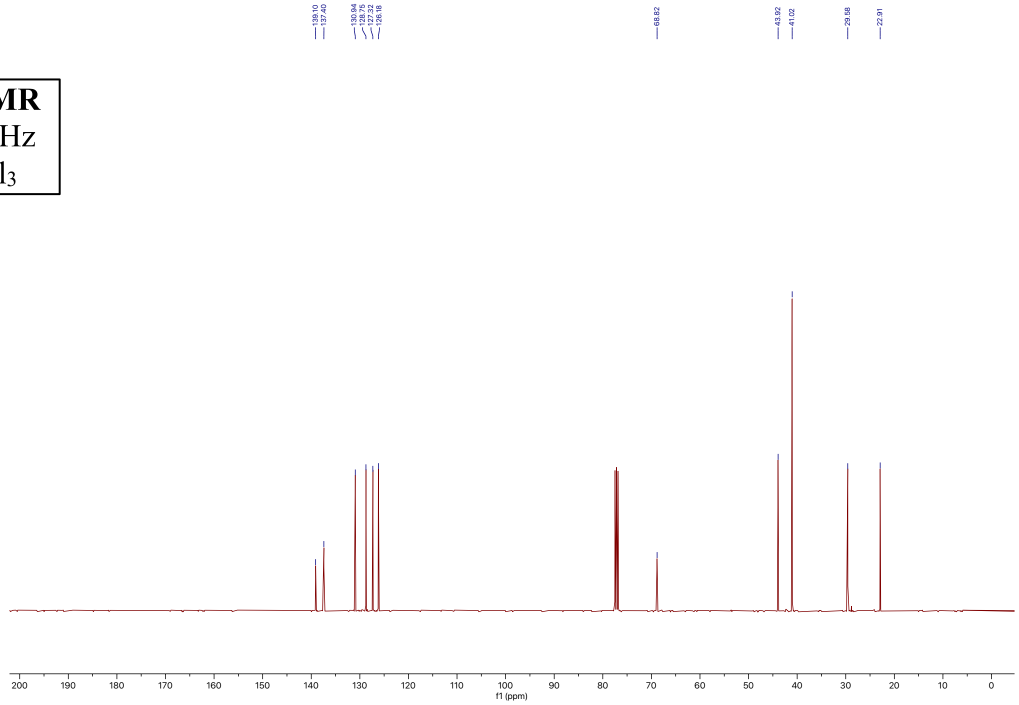

2-Ethyl-2-phenyl-1,3-dithiolane (SM-20)

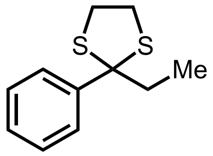

^{1}H-NMR
400 MHz
$CDCl_3$

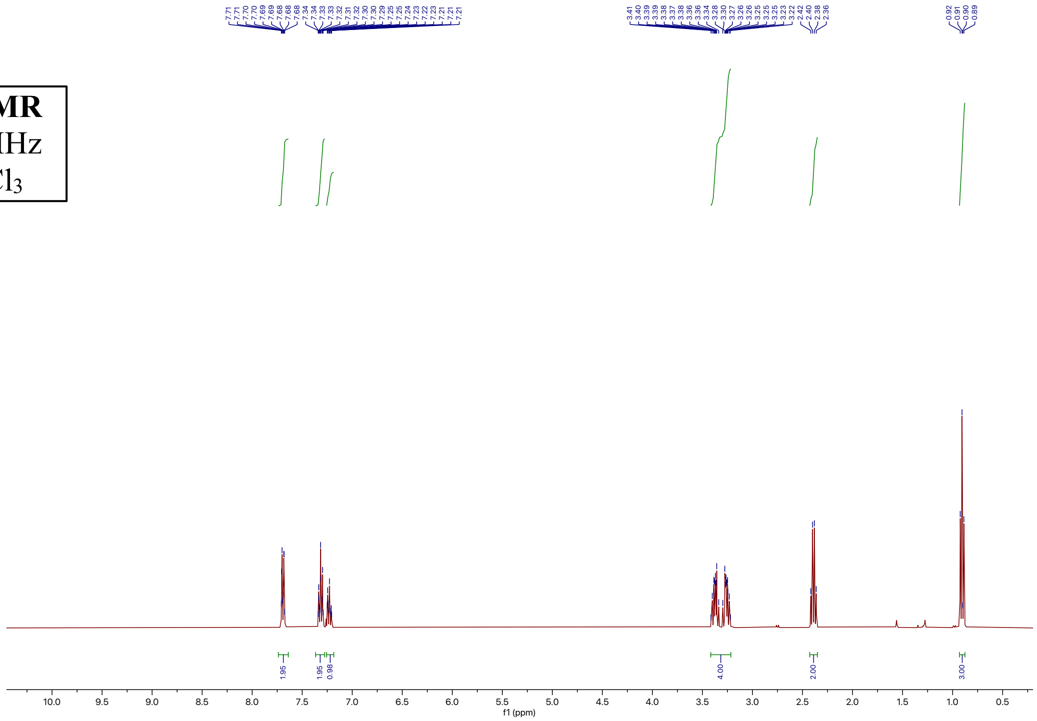

^{13}C-NMR
101 MHz
$CDCl_3$

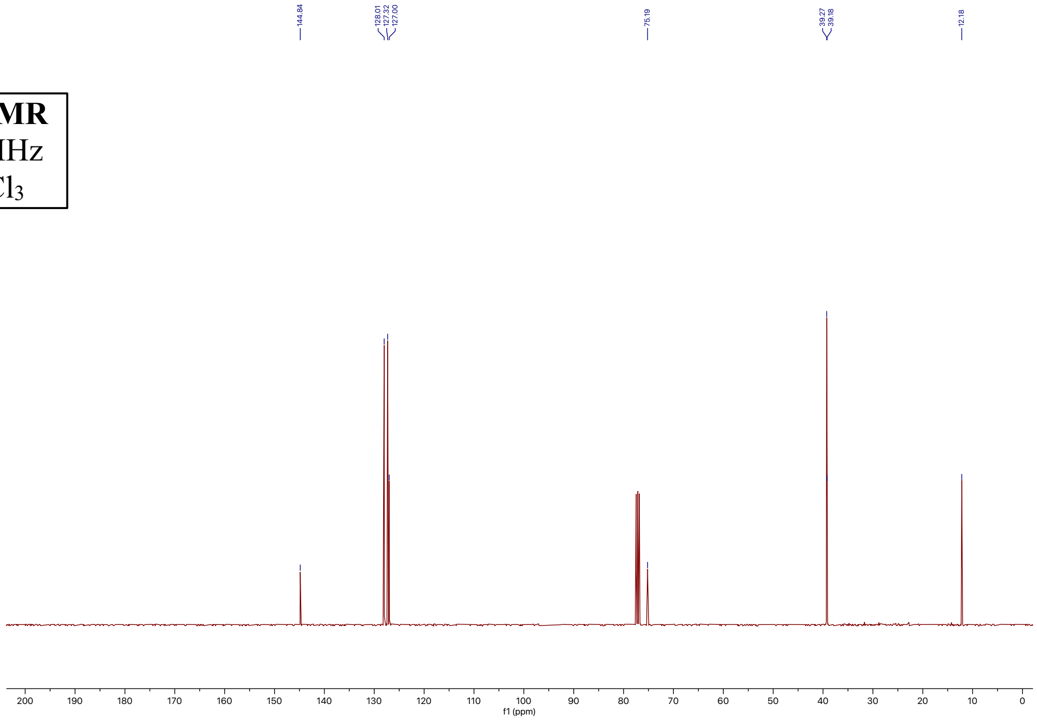

8-Phenyl-1,4-dithiaspiro[4.5]decane (SM-21)

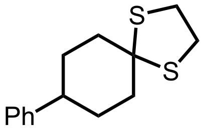

^{1}H-NMR
400 MHz
$CDCl_3$

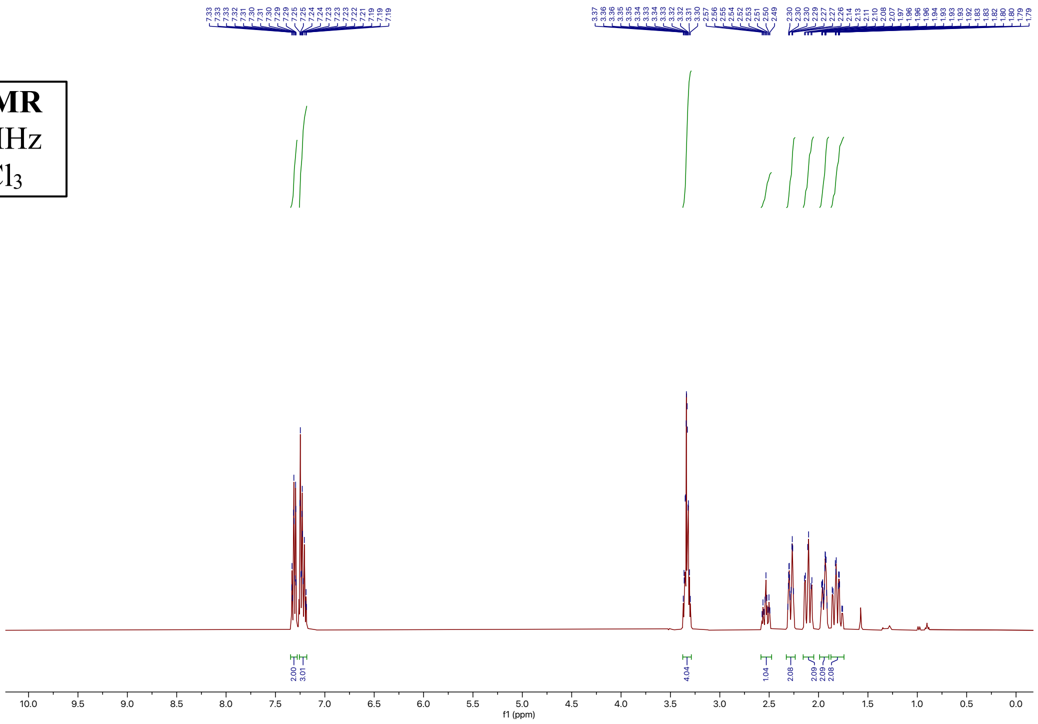

^{13}C-NMR
101 MHz
$CDCl_3$

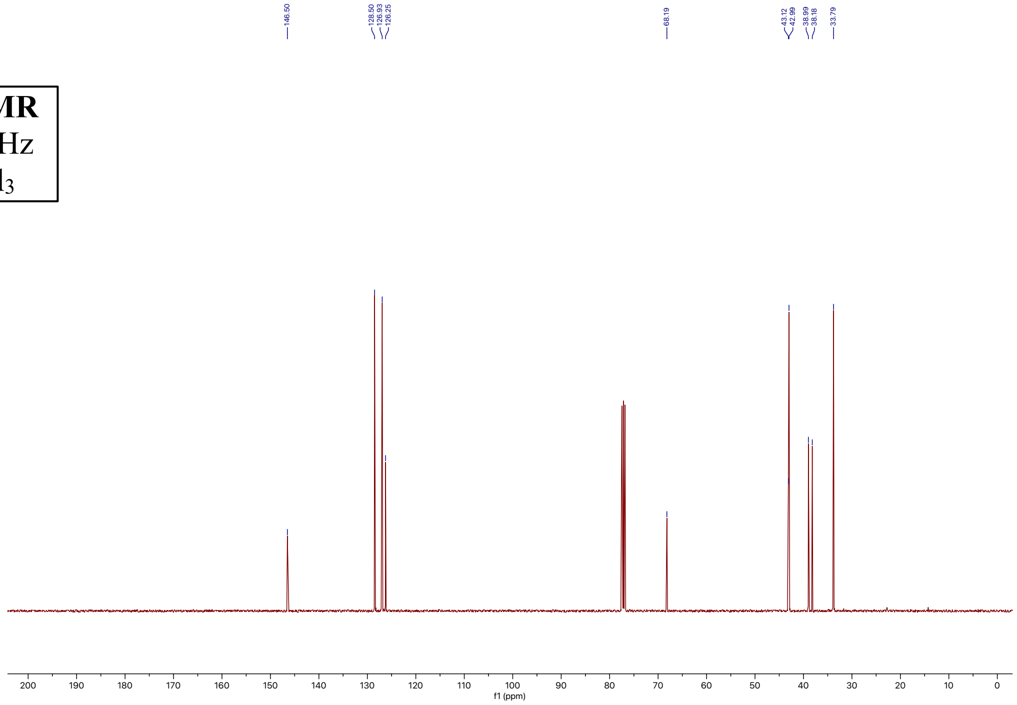

2-Phenyl-1,3-dithiolane (SM-22)

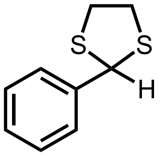

^{1}H-NMR
400 MHz
$CDCl_3$

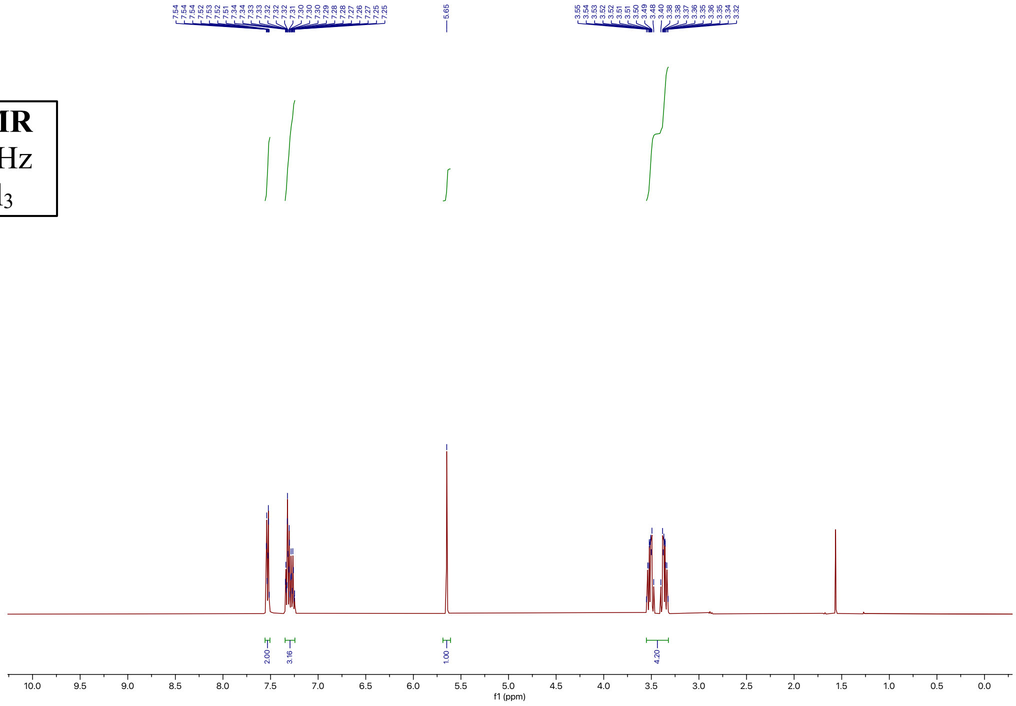

^{13}C-NMR
101 MHz
$CDCl_3$

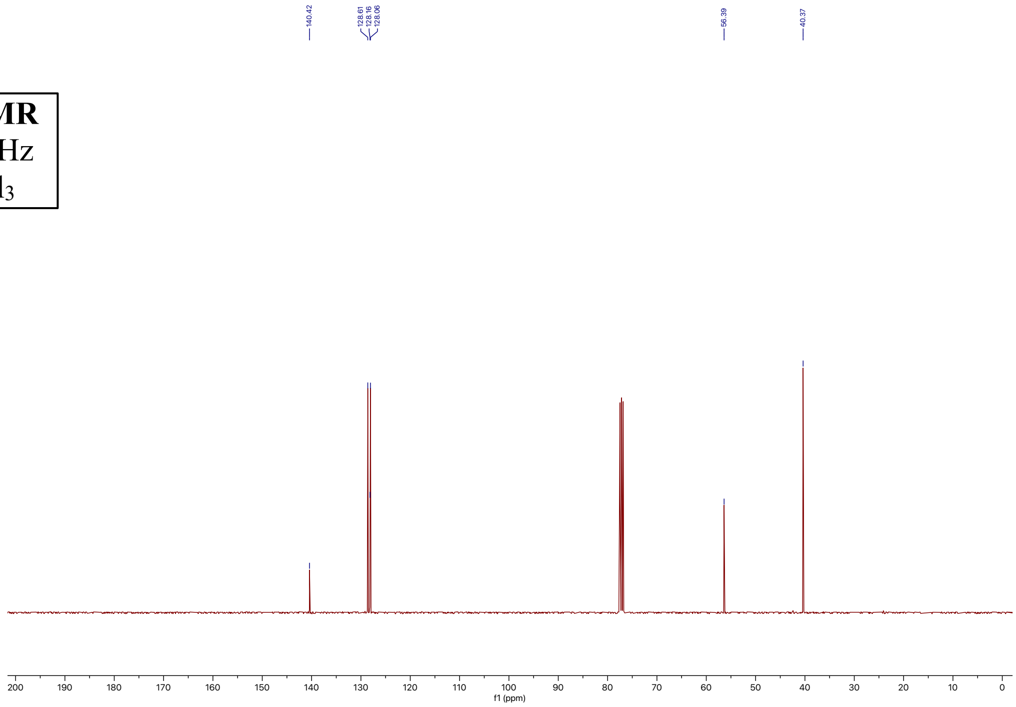

4-(1,3-Dithiolan-2-yl)-2-methoxyphenol (SM-23)

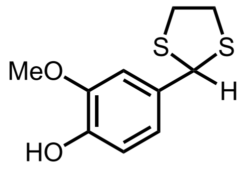

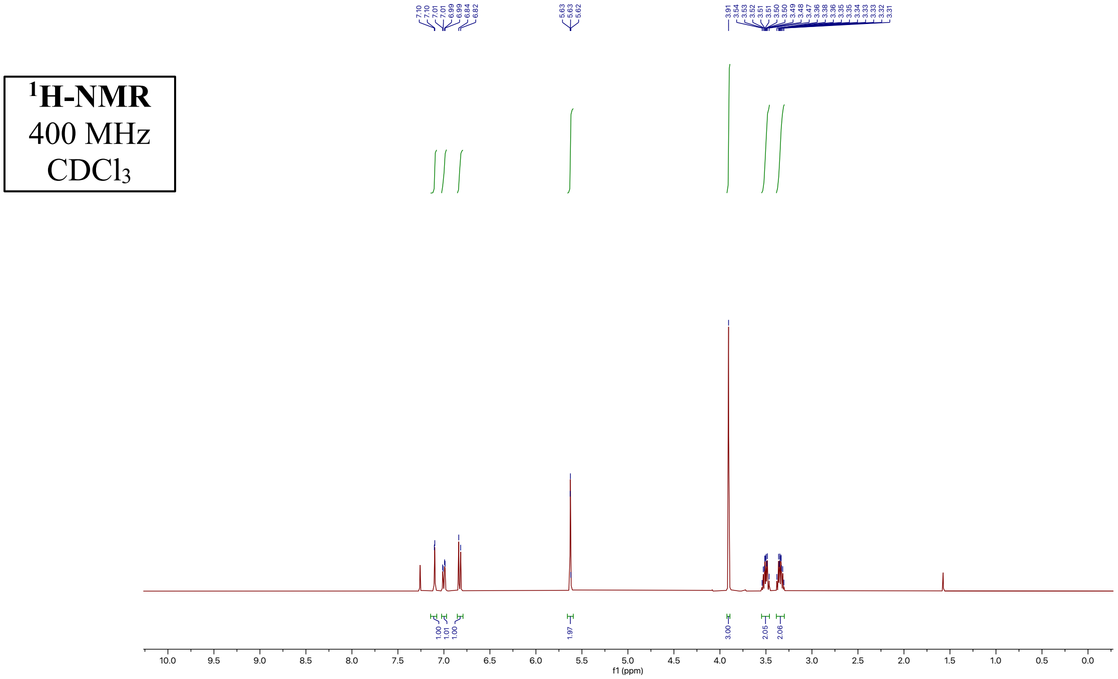

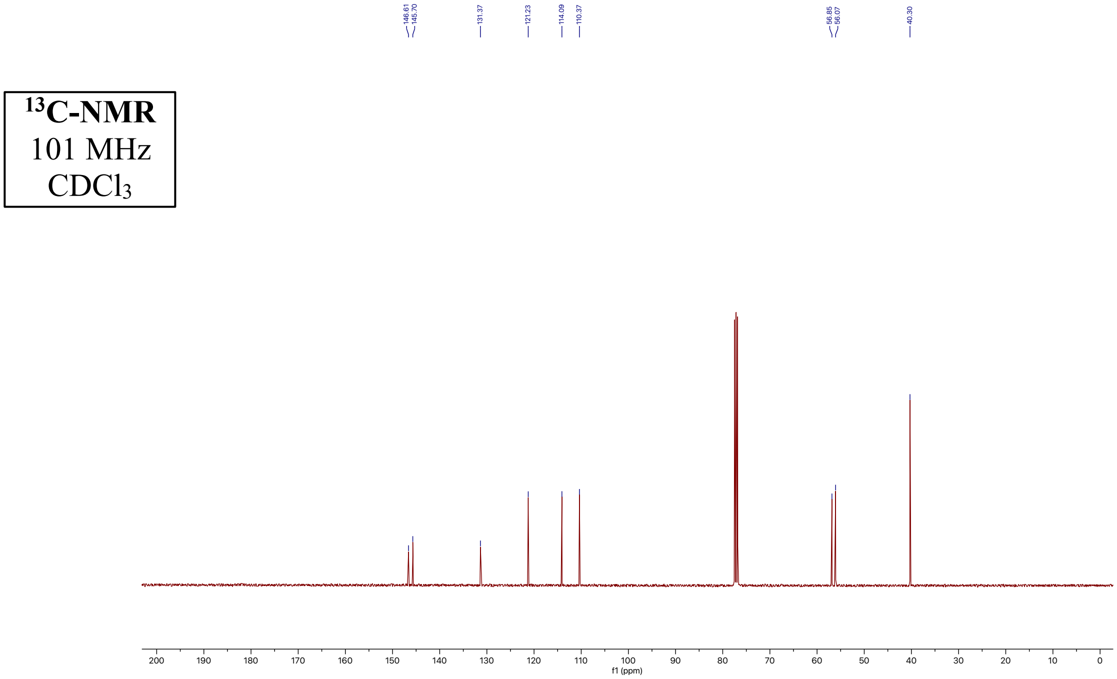

4-(1,3-Dithiolan-2-yl)-N,N-dimethylaniline (SM-24)

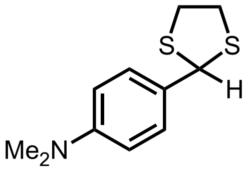

^{1}H-NMR
400 MHz
$CDCl_3$

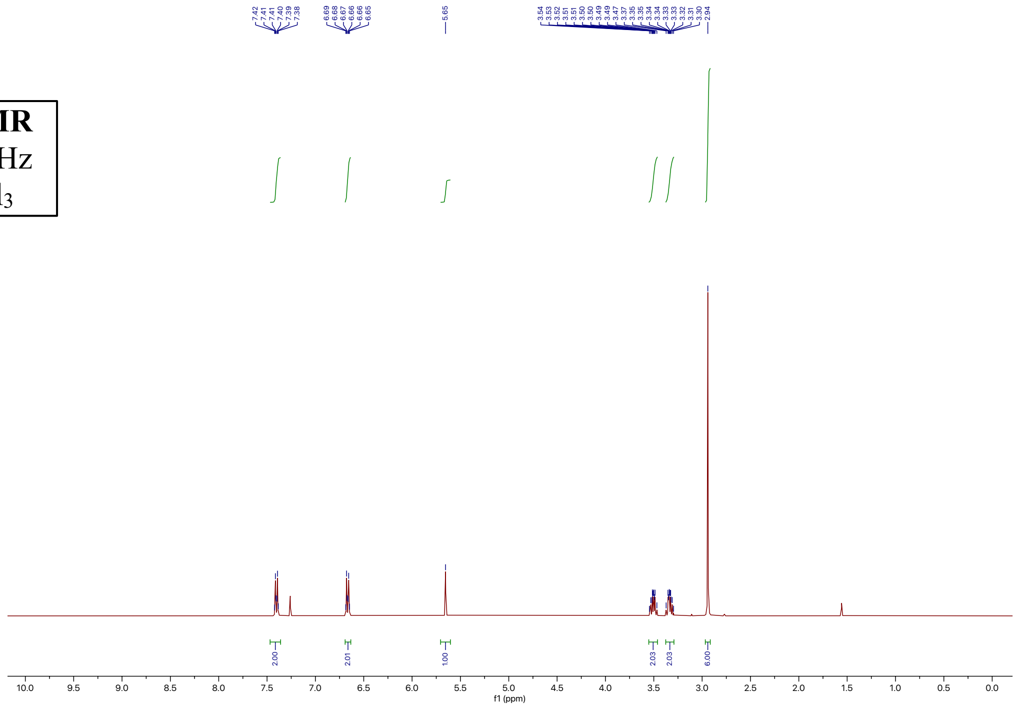

^{13}C-NMR
101 MHz
$CDCl_3$

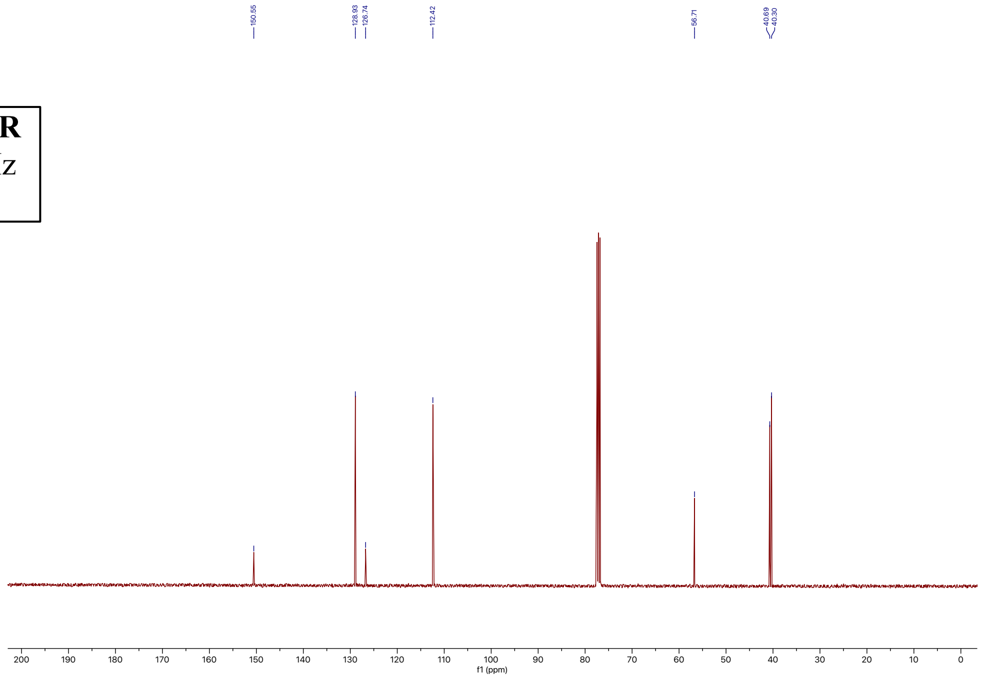

3-(1,3-Dithiolan-2-yl)pyridine (SM-25)

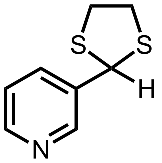

^{1}H-NMR
400 MHz
$CDCl_3$

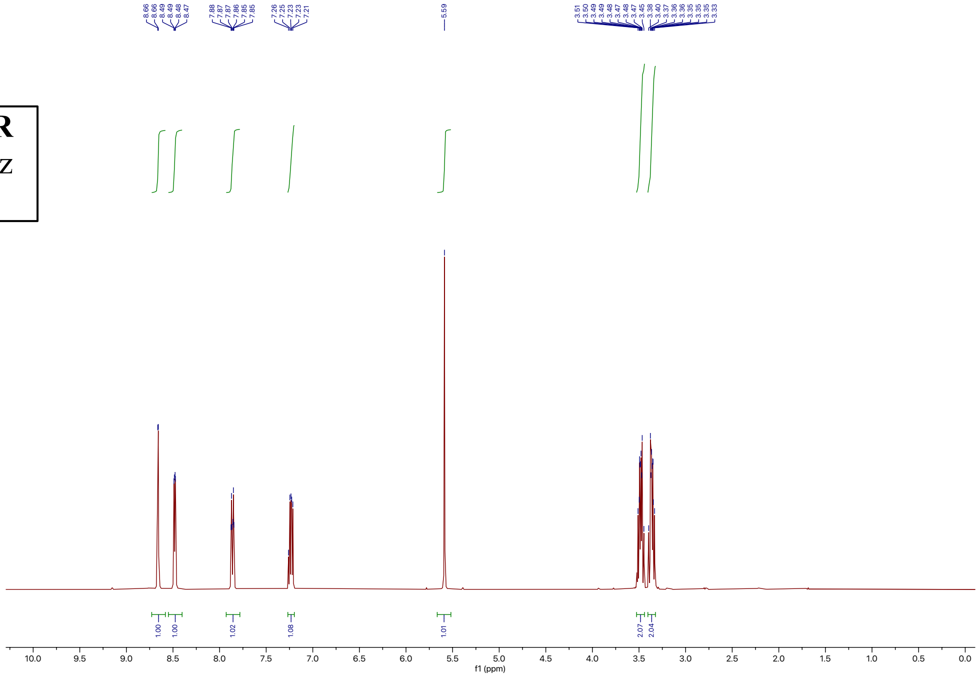

^{13}C-NMR
101 MHz
$CDCl_3$

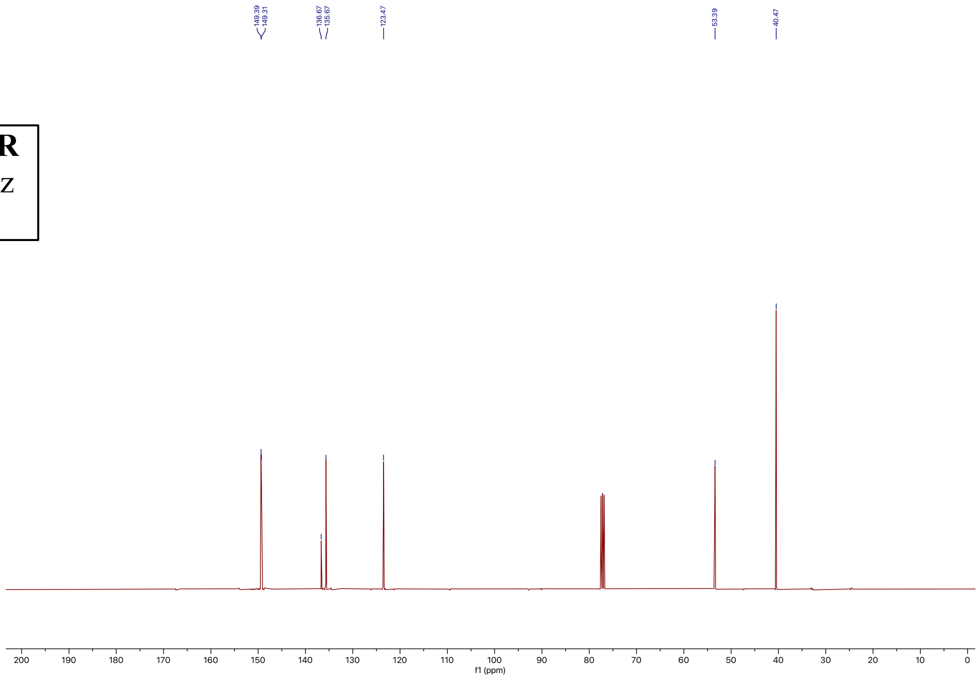

3-(1,3-Dithiolan-2-yl)-1H-indole (SM-26)

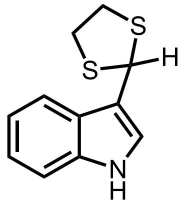

^{1}H-NMR
400 MHz
$CDCl_3$

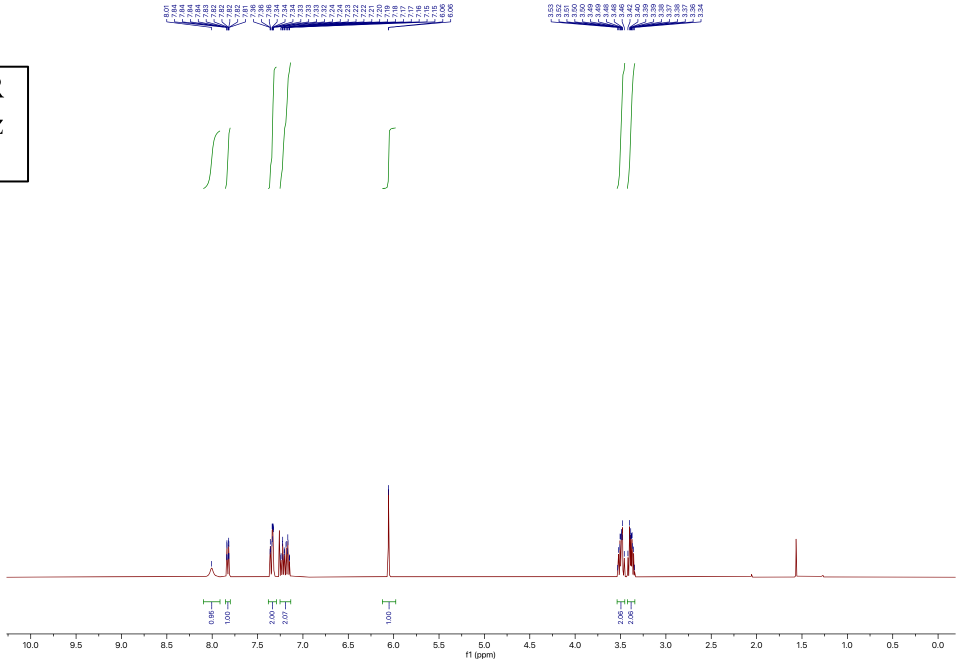

^{13}C-NMR
101 MHz
$CDCl_3$

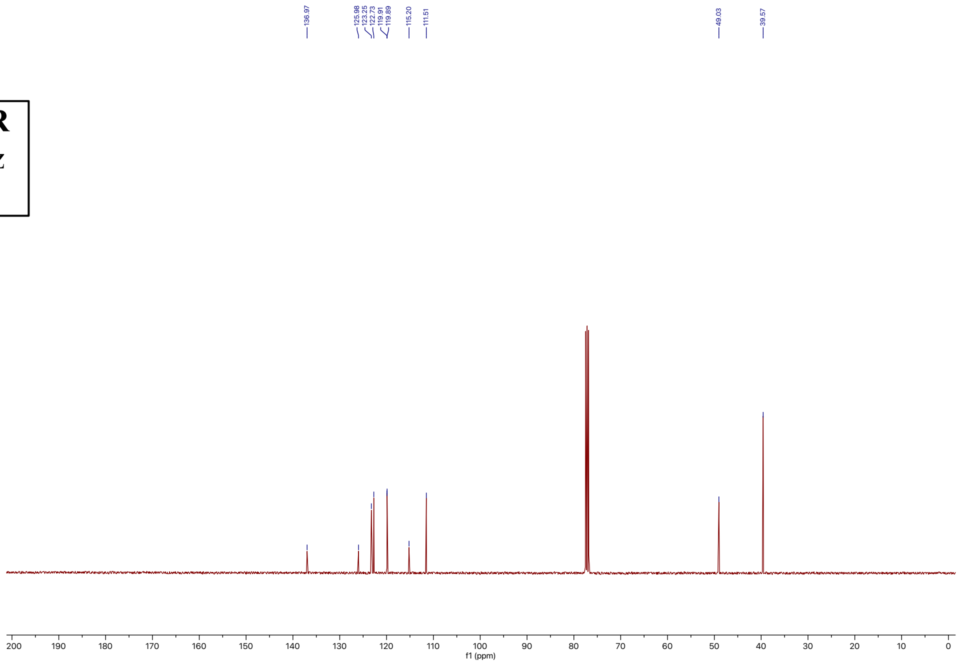

Acetophenone (2)

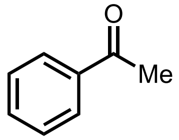

^{1}H-NMR
400 MHz
$CDCl_3$

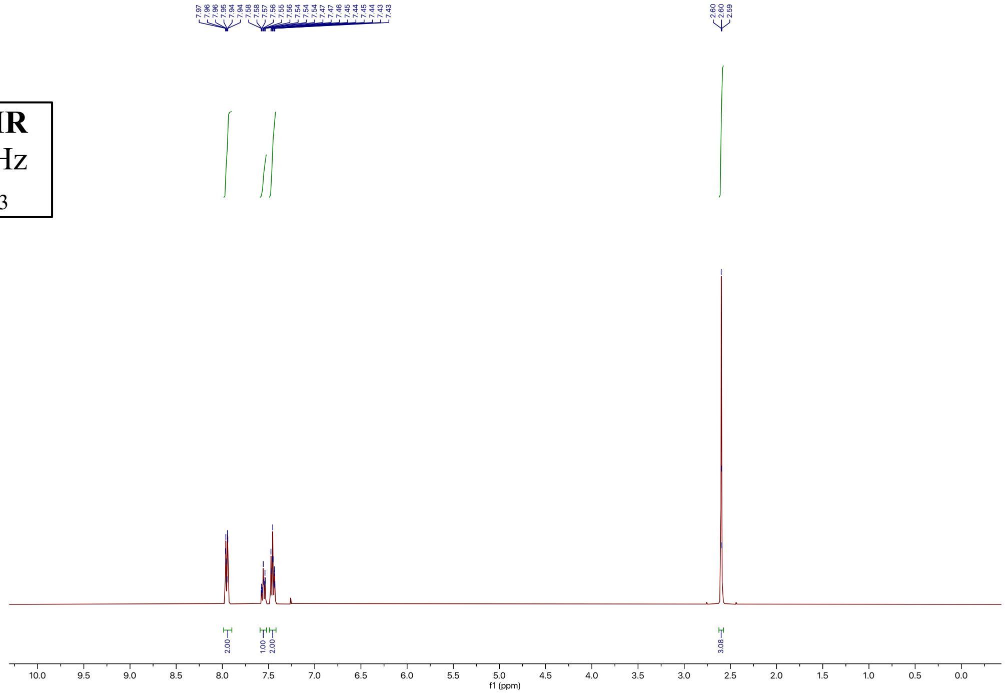

^{13}C-NMR
101 MHz
$CDCl_3$

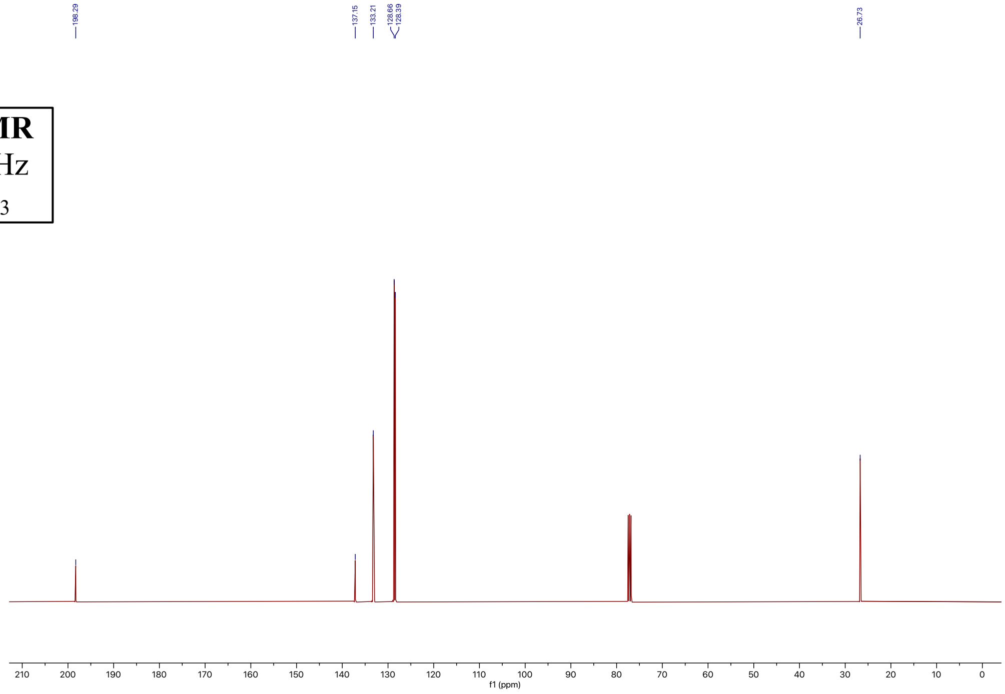

1-(4-Bromophenyl)ethan-1-one (3)

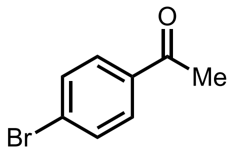

^{1}H-NMR
400 MHz
$CDCl_3$

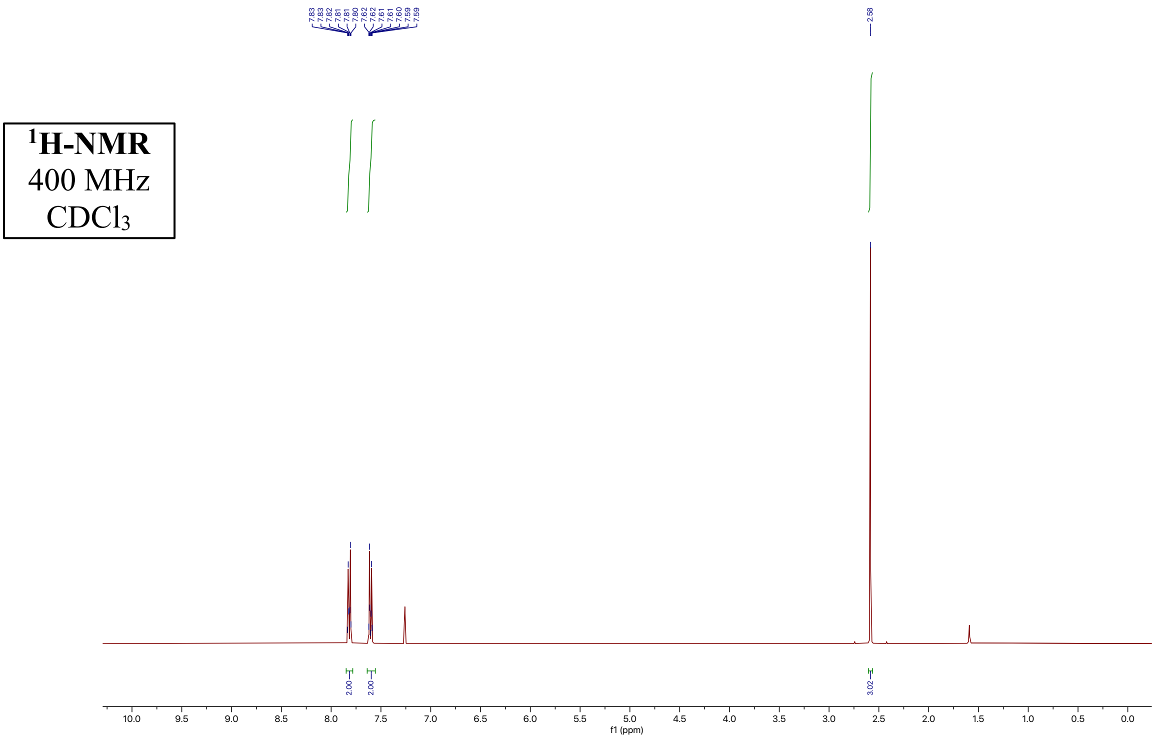

^{13}C-NMR
101 MHz
$CDCl_3$

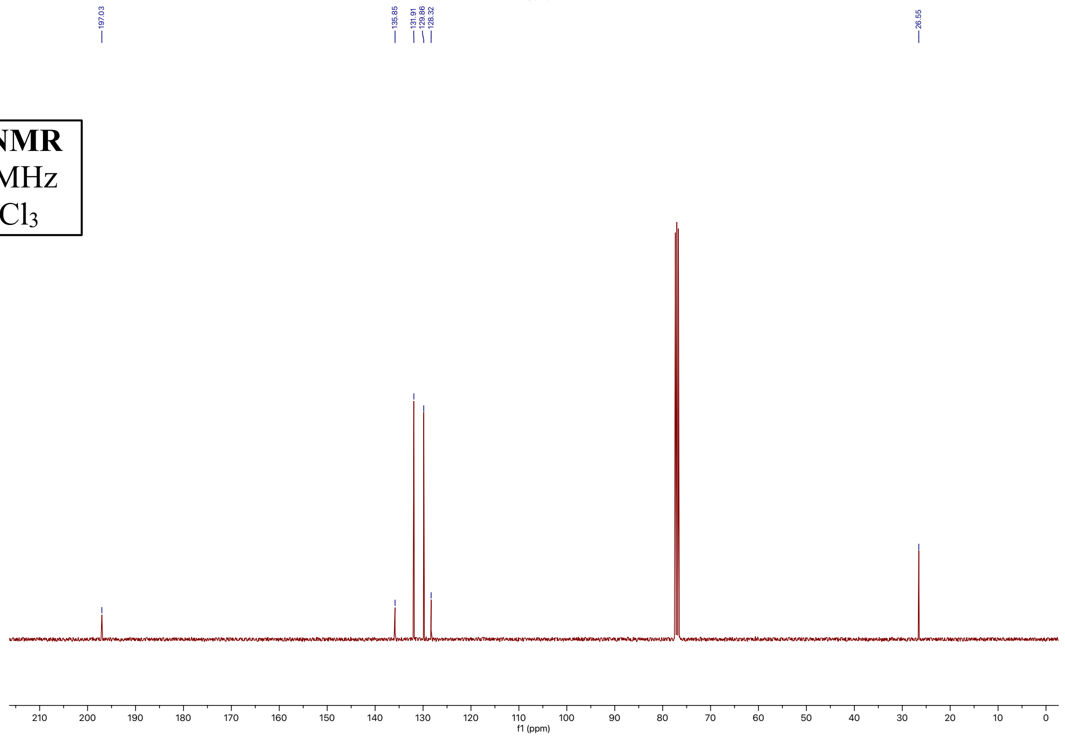

1-(4-Chlorophenyl)ethan-1-one (4)

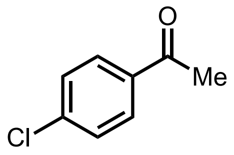

^{1}H-NMR
400 MHz
$CDCl_3$

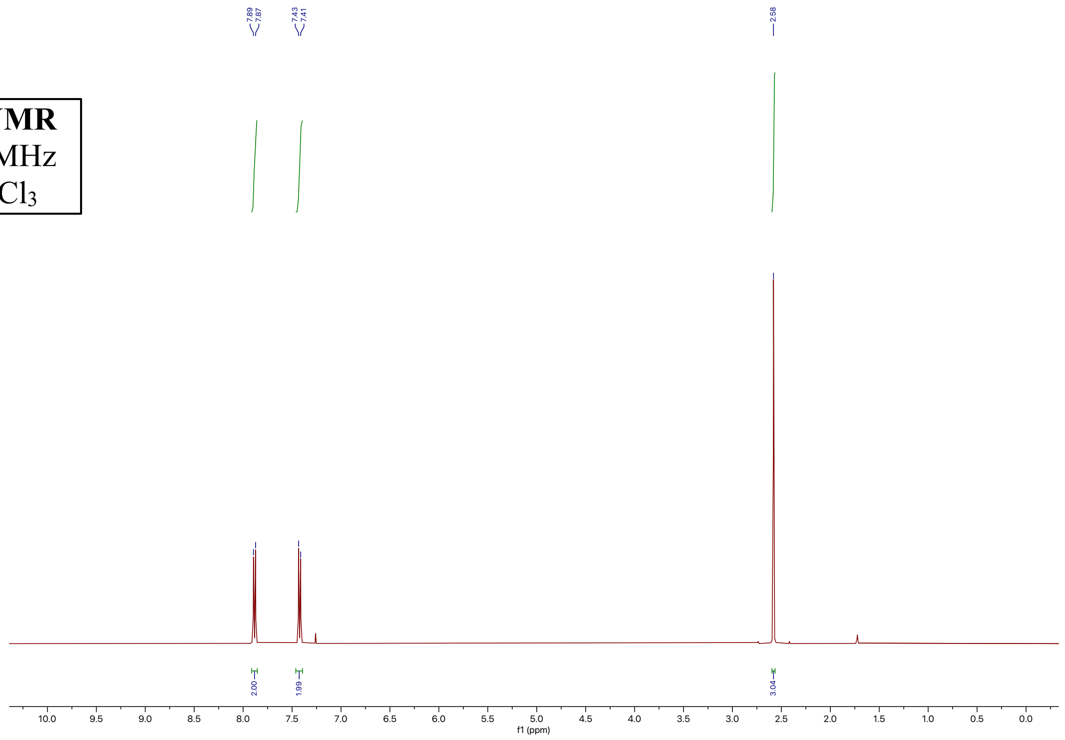

^{13}C-NMR
101 MHz
$CDCl_3$

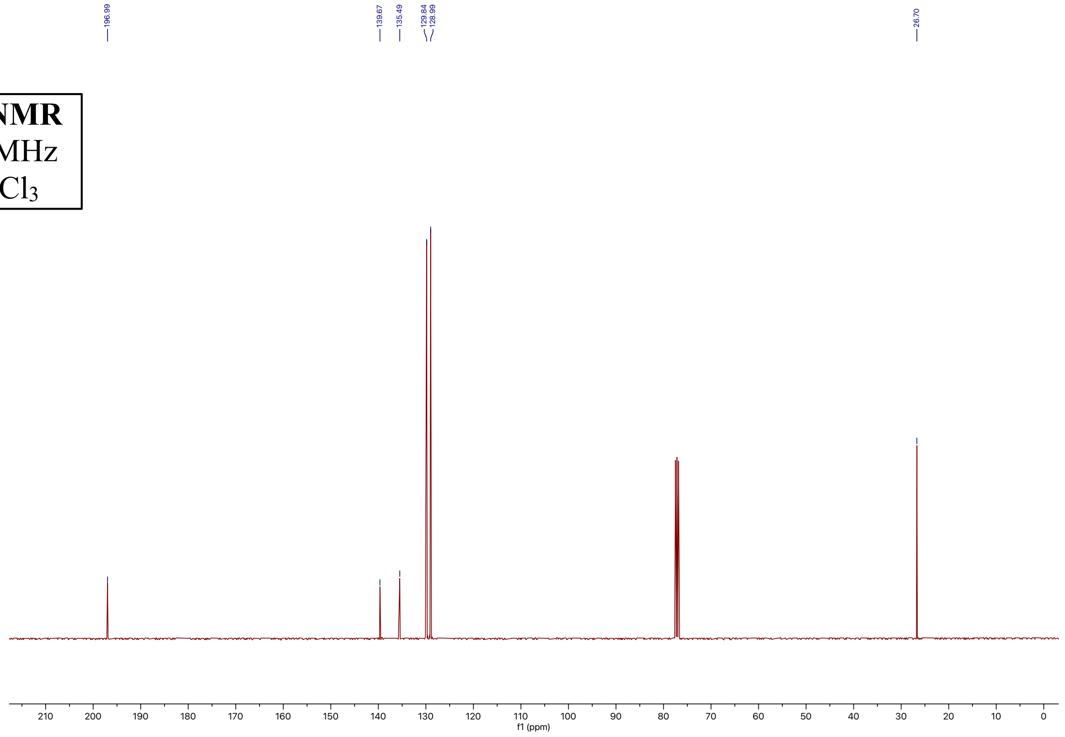

1-(4-Fluorophenyl)ethan-1-one (5)

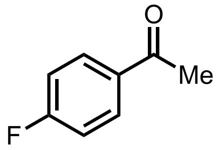

^{1}H-NMR
400 MHz
$CDCl_3$

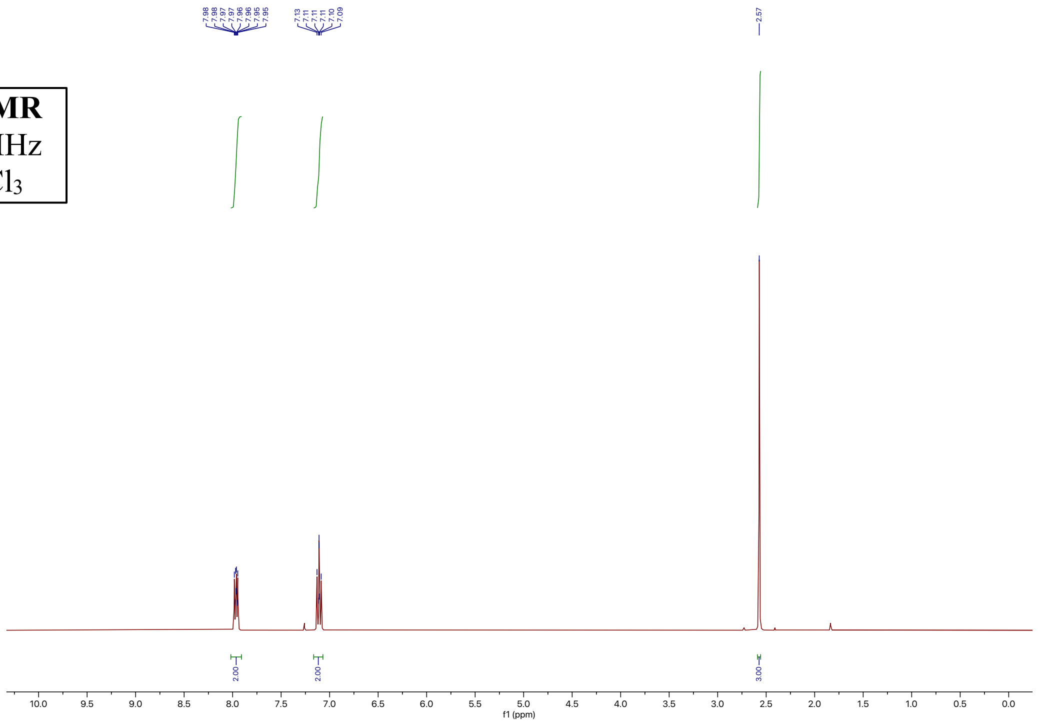

^{13}C-NMR
101 MHz
$CDCl_3$

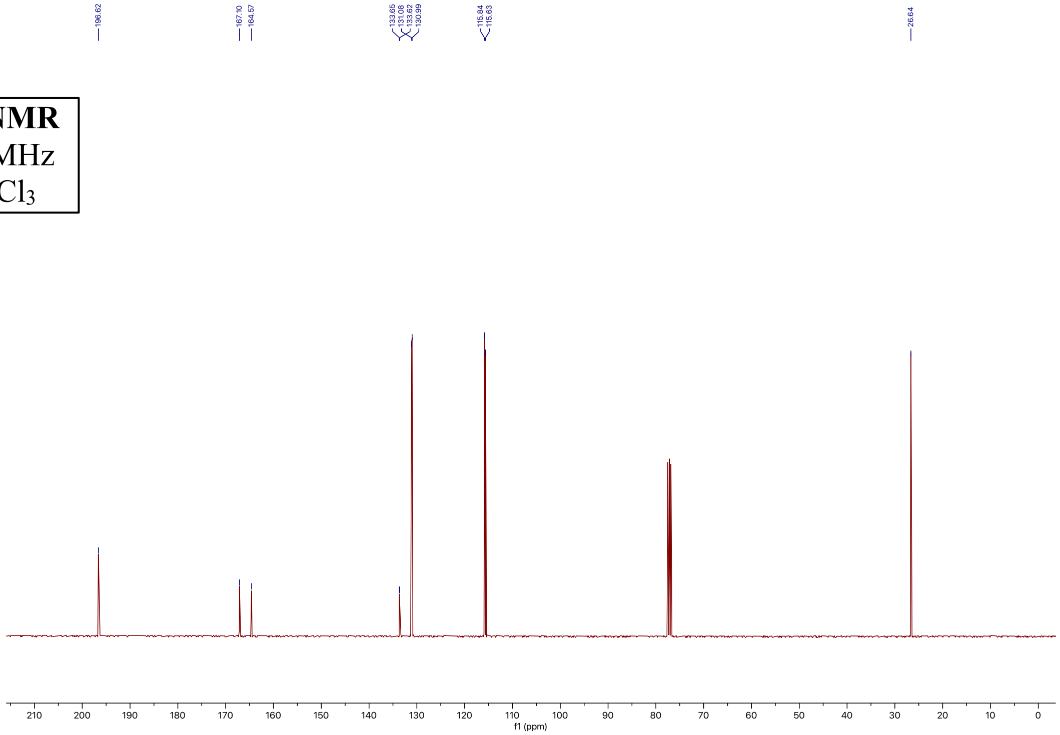

1-(4-Methoxyphenyl)ethan-1-one (6)

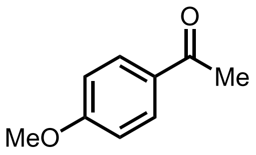

^{1}H-NMR
400 MHz
$CDCl_3$

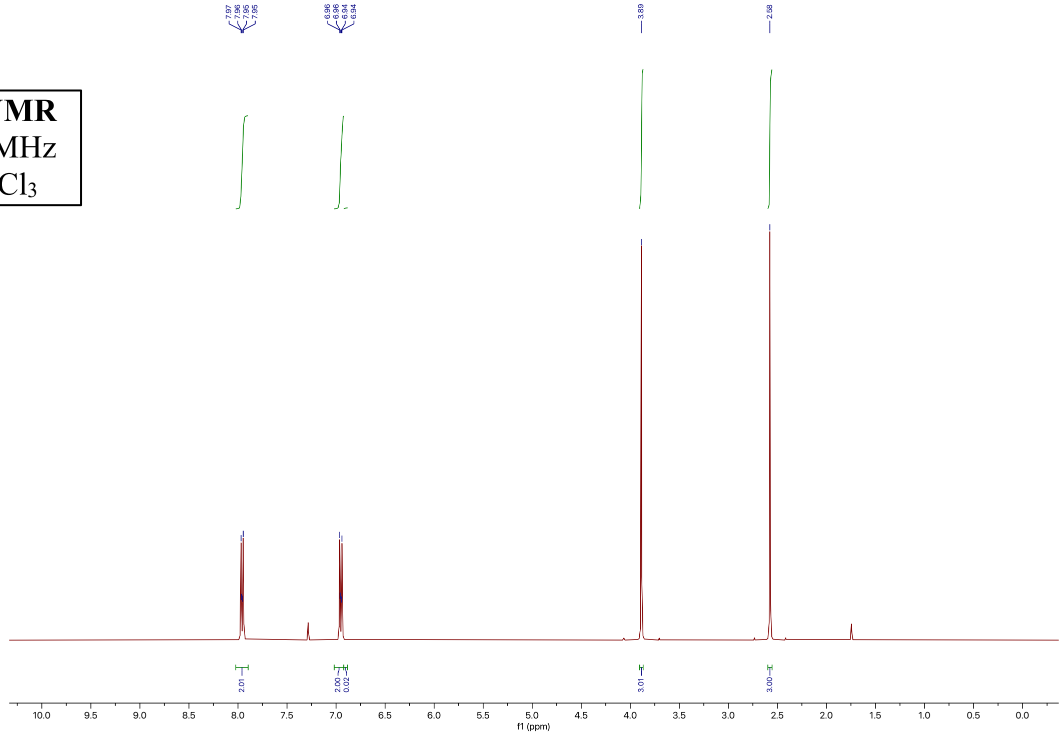

^{13}C-NMR
101 MHz
$CDCl_3$

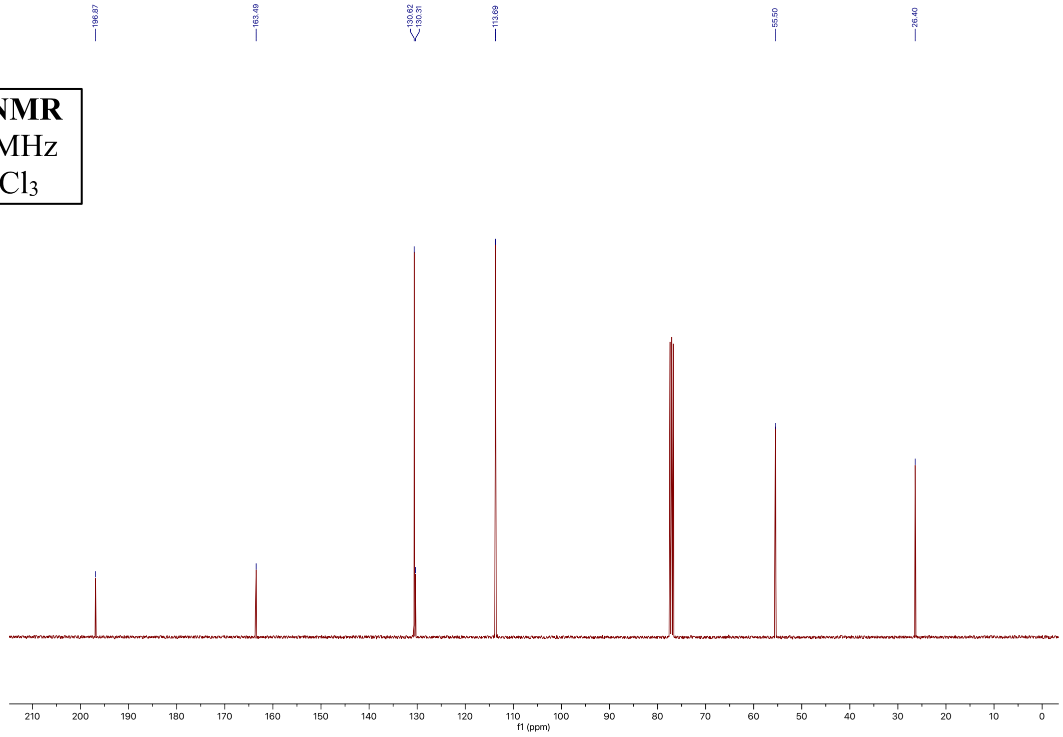

1-(*p*-Tolyl)ethan-1-one (7)

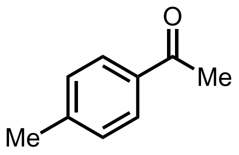

^{1}H-NMR
400 MHz
$CDCl_3$

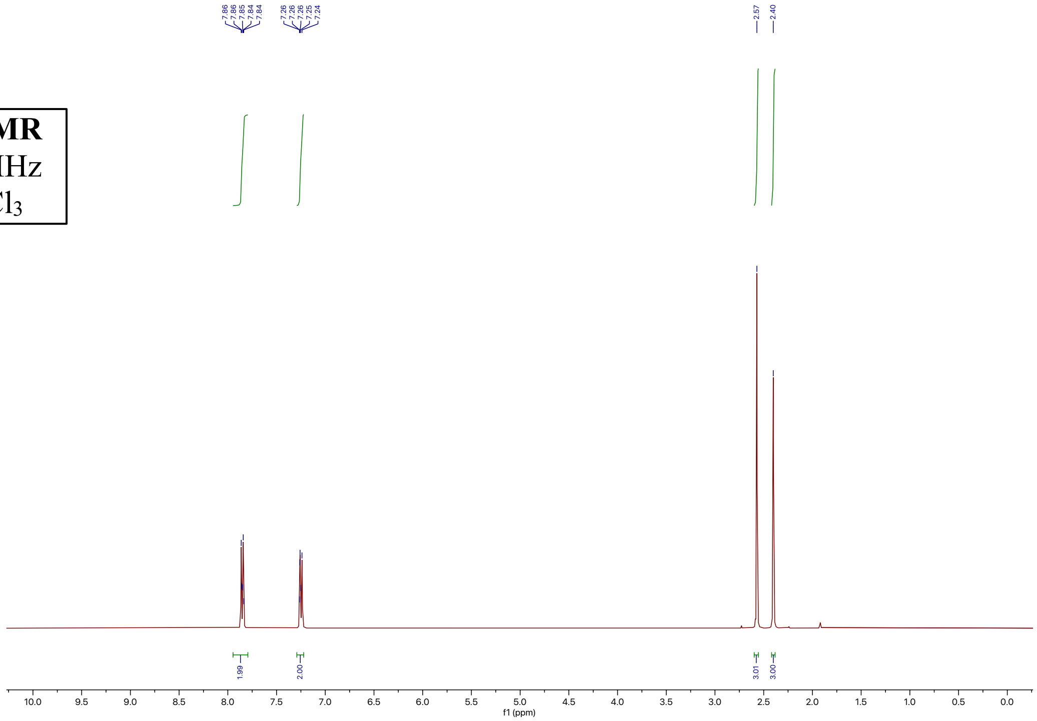

^{13}C-NMR
101 MHz
$CDCl_3$

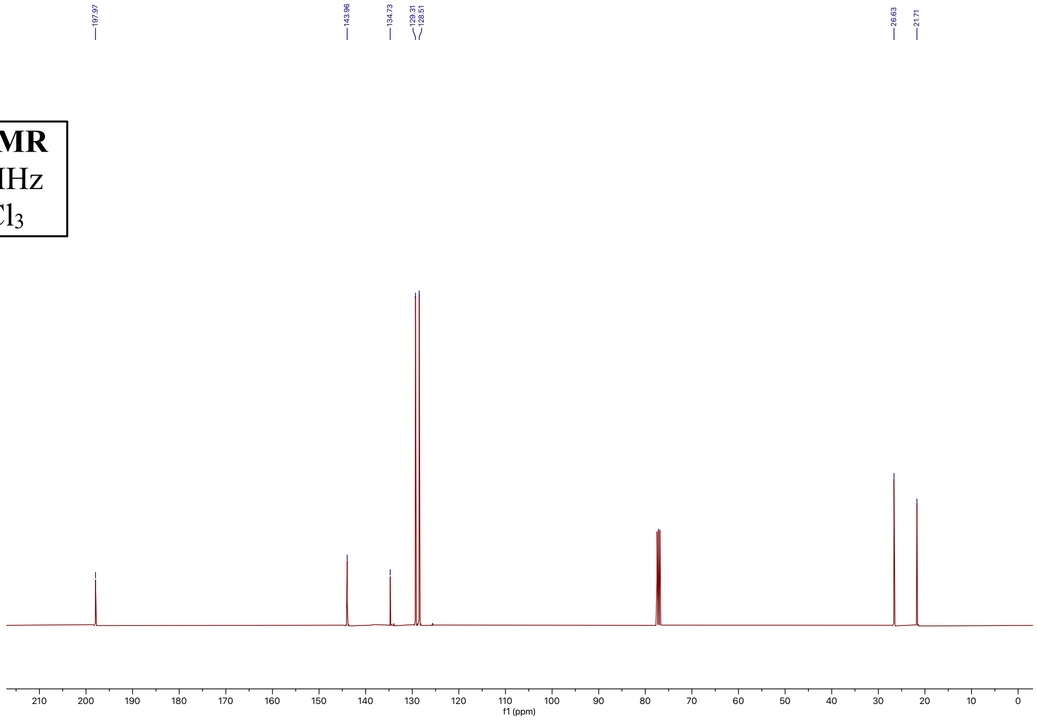

1-(4-(*tert*-Butyl)phenyl)ethan-1-one (8)

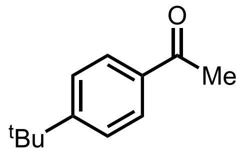

^{1}H-NMR
400 MHz
$CDCl_3$

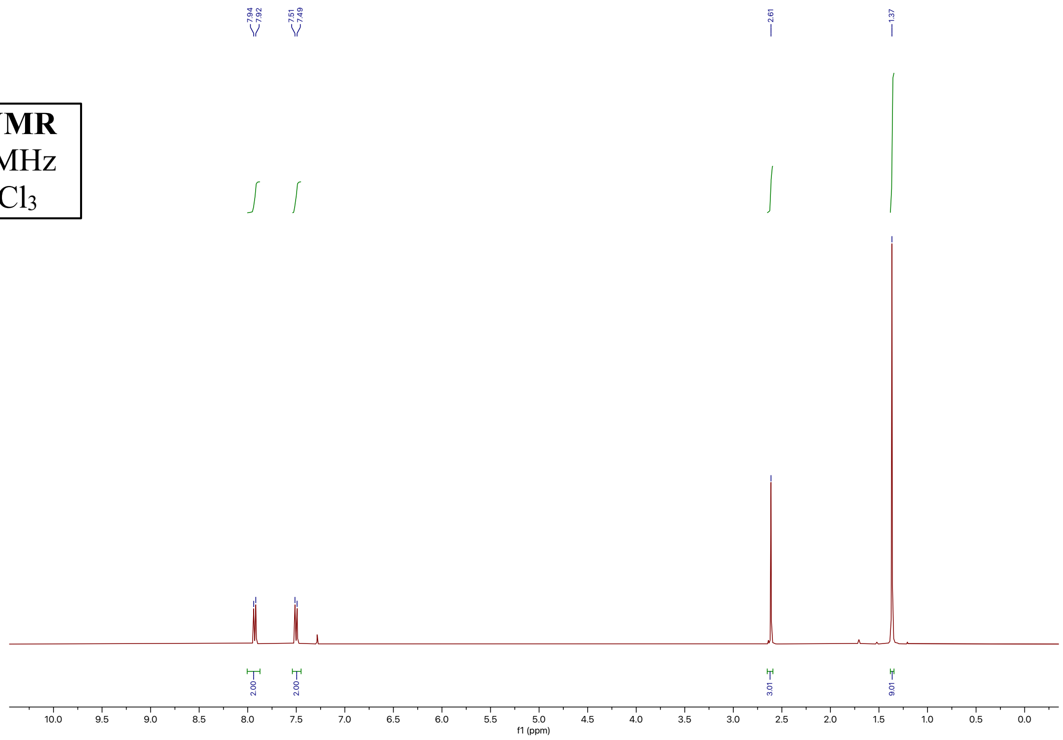

^{13}C-NMR
101 MHz
$CDCl_3$

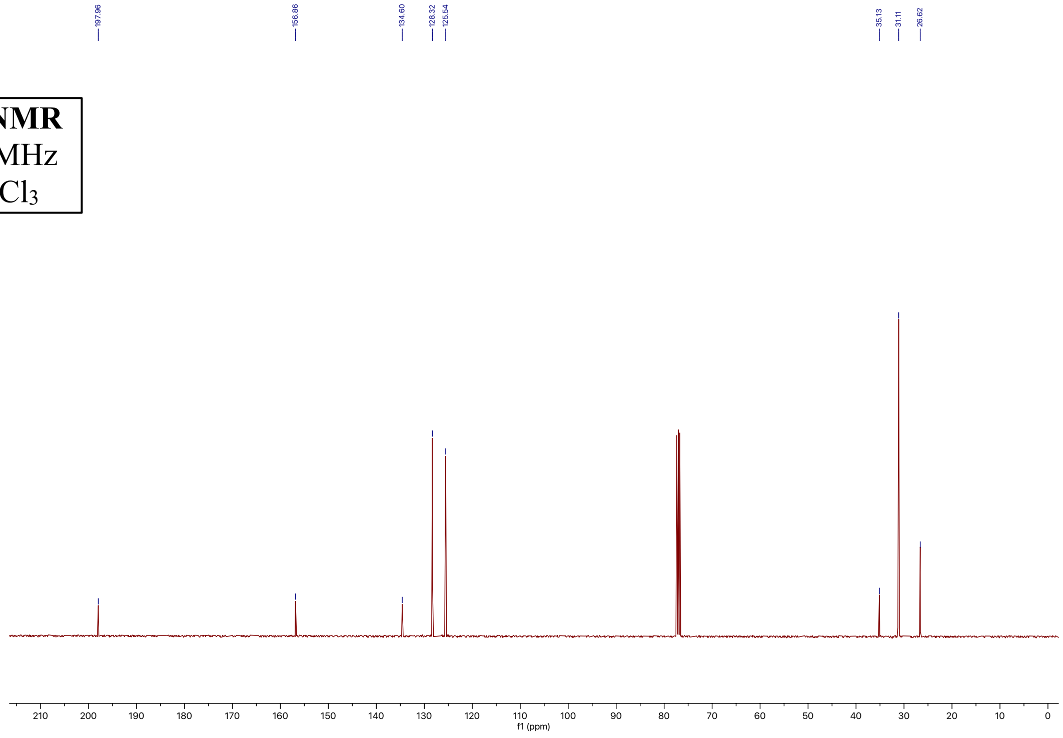

1-(4-Nitrophenyl)ethan-1-one (9)

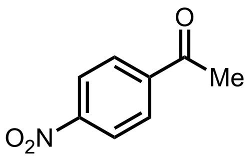

1H-NMR
400 MHz
$CDCl_3$

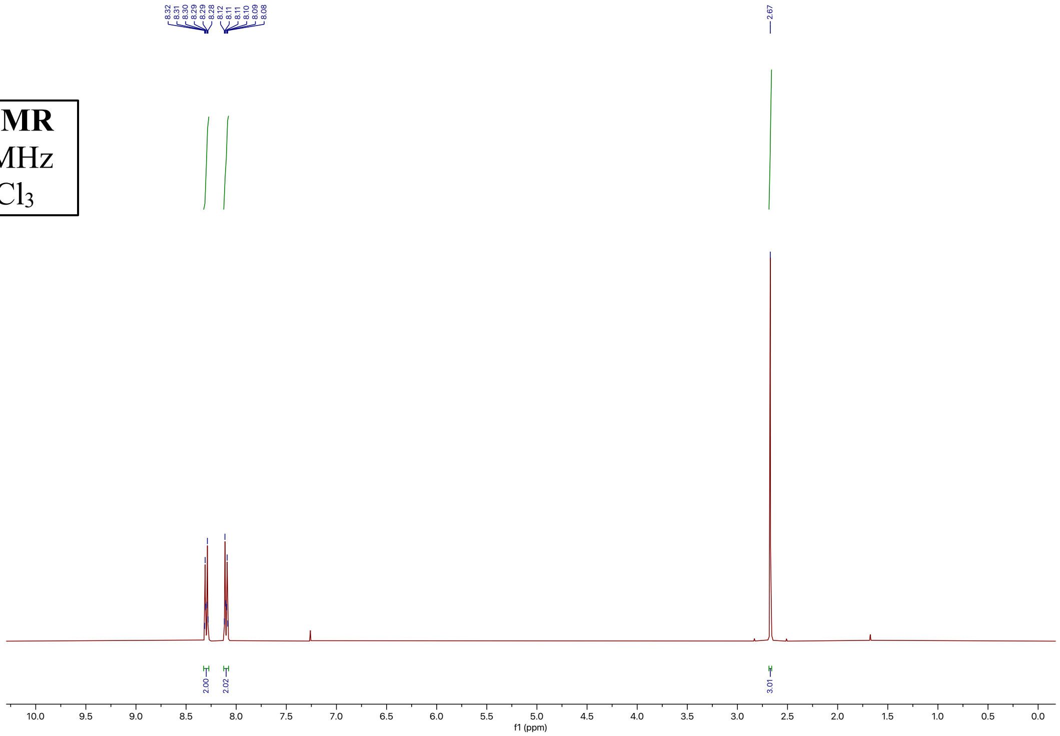

13C-NMR
101 MHz
$CDCl_3$

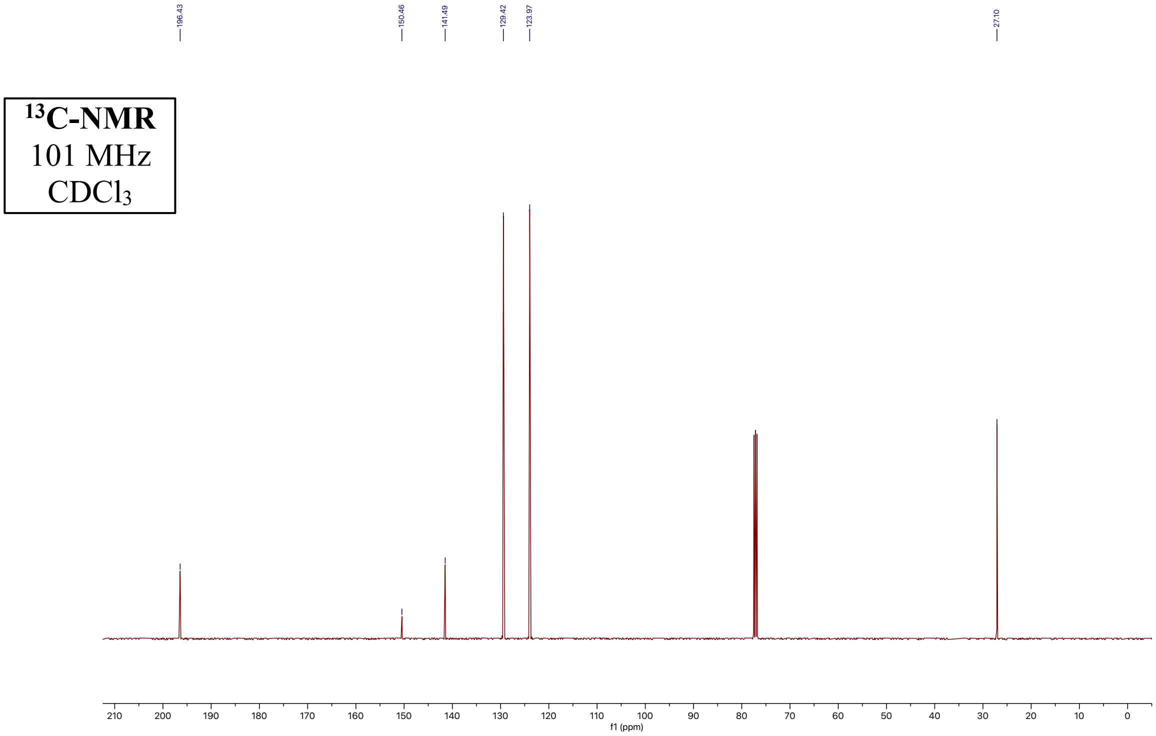

1-([1,1'-Biphenyl]-4-yl)ethan-1-one (10)

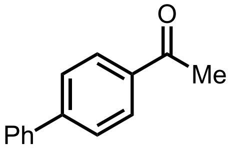

^{1}H-NMR
400 MHz
$CDCl_3$

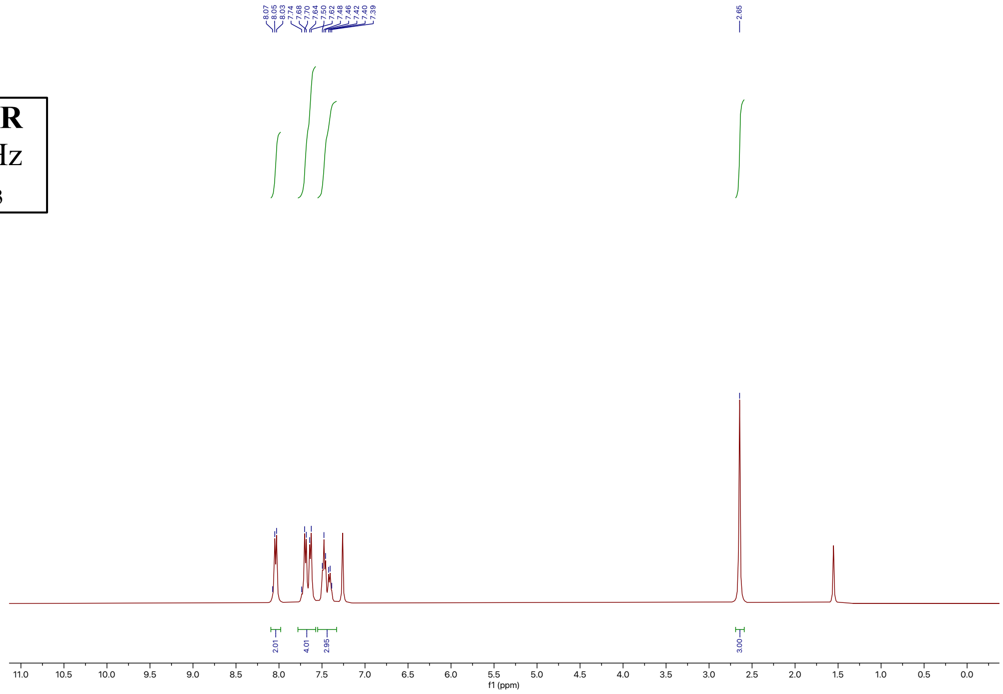

^{13}C-NMR
101 MHz
$CDCl_3$

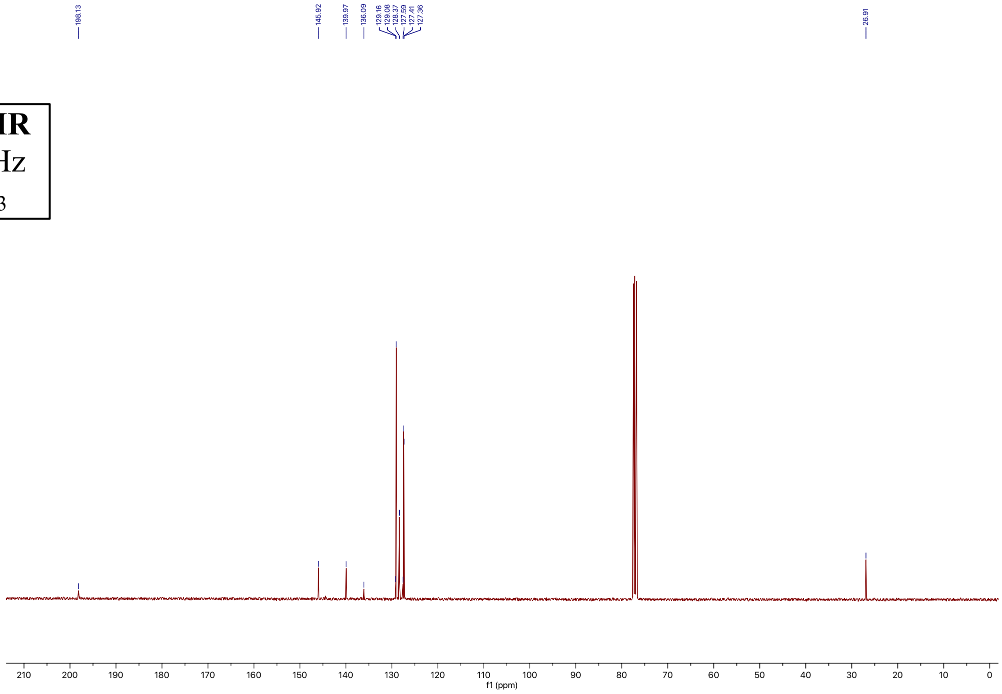

Methyl 4-acetylbenzoate (11)

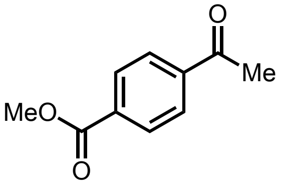

^{1}H-NMR
400 MHz
$CDCl_3$

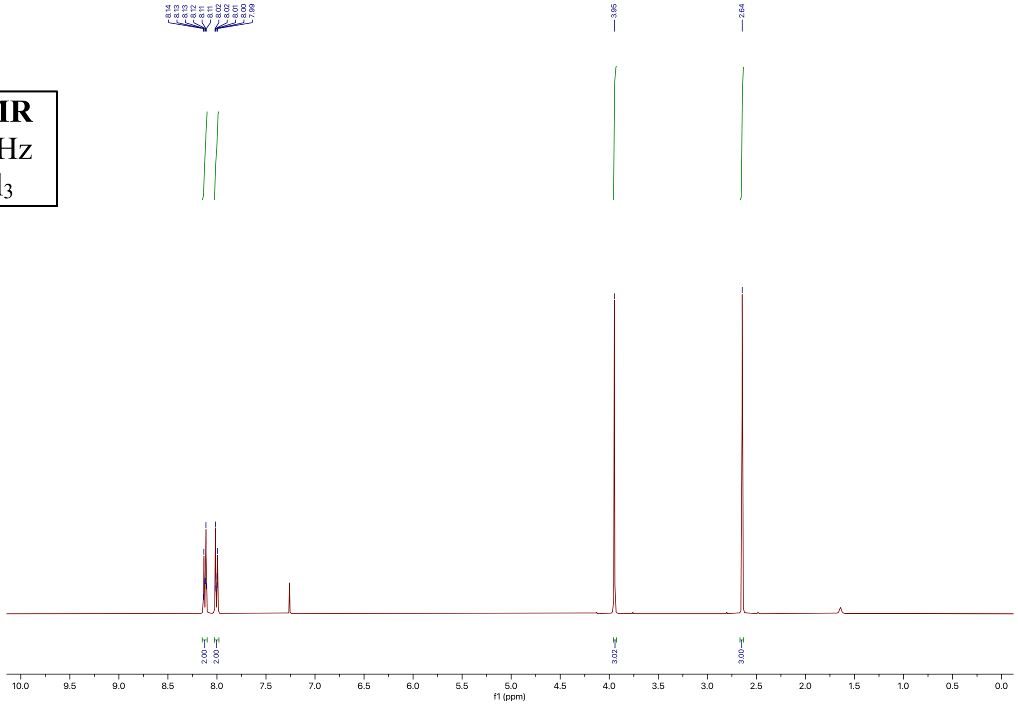

^{13}C-NMR
101 MHz
$CDCl_3$

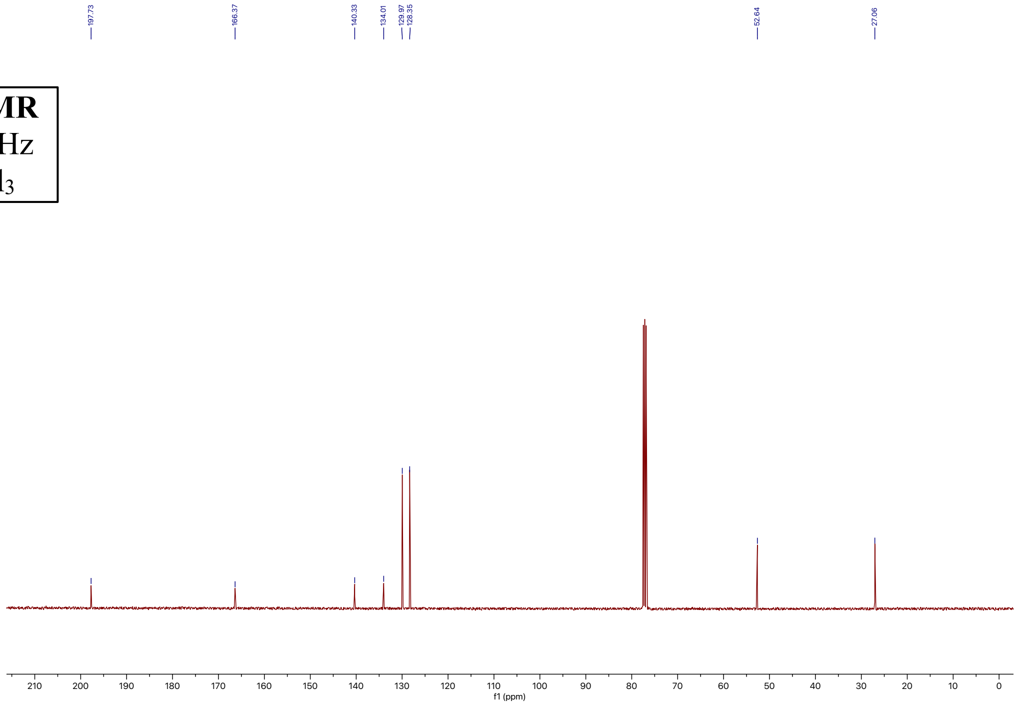

1-(3-Methoxyphenyl)ethan-1-one (12)

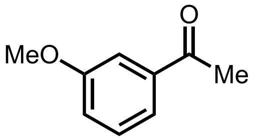

^{1}H-NMR
400 MHz
$CDCl_3$

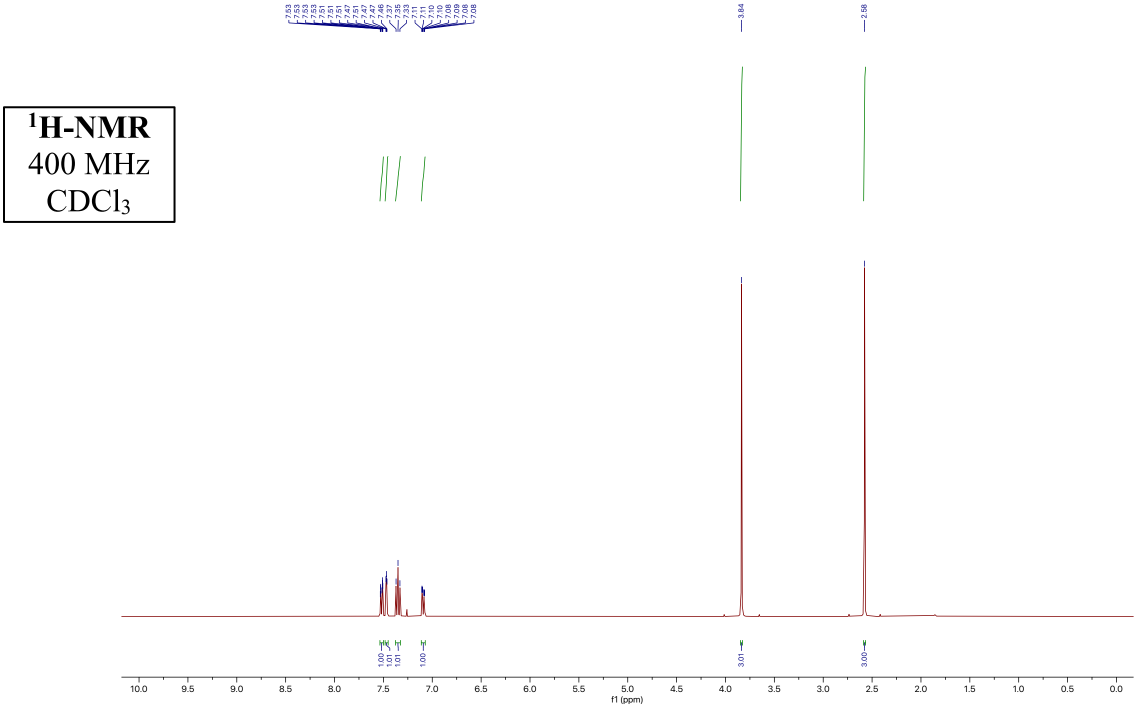

^{13}C-NMR
101 MHz
$CDCl_3$

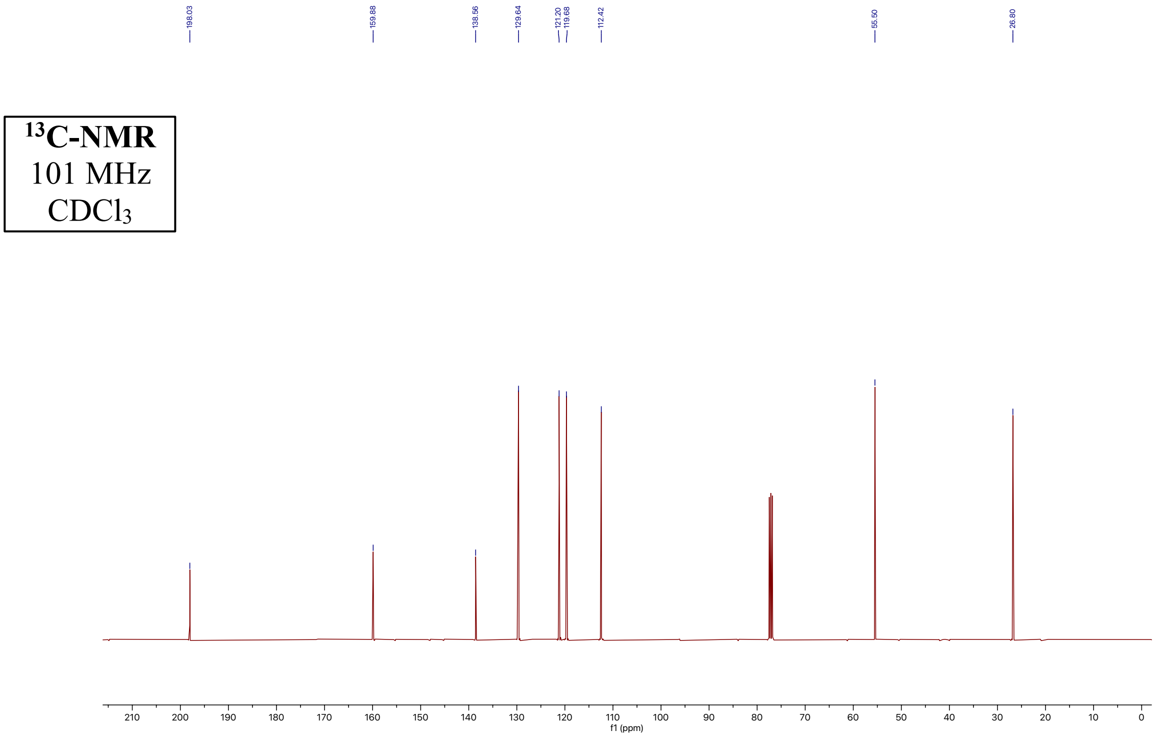

1-(*m*-Tolyl)ethan-1-one (13)

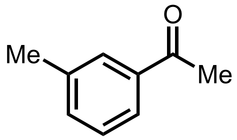

^{1}H-NMR
400 MHz
$CDCl_3$

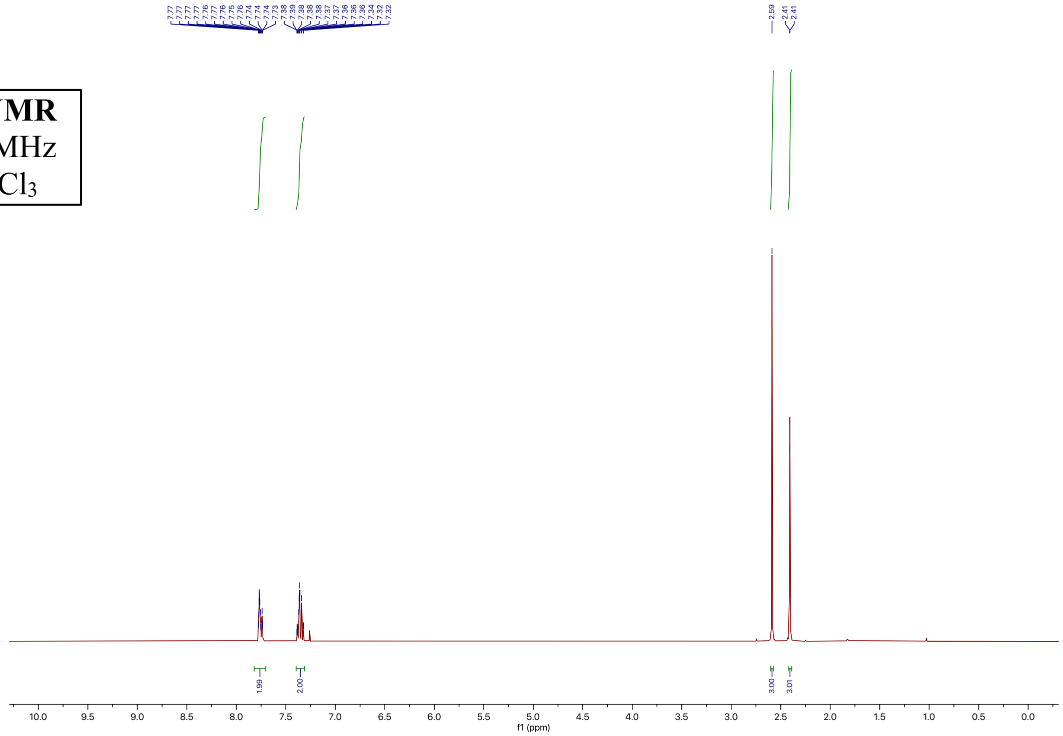

^{13}C-NMR
101 MHz
$CDCl_3$

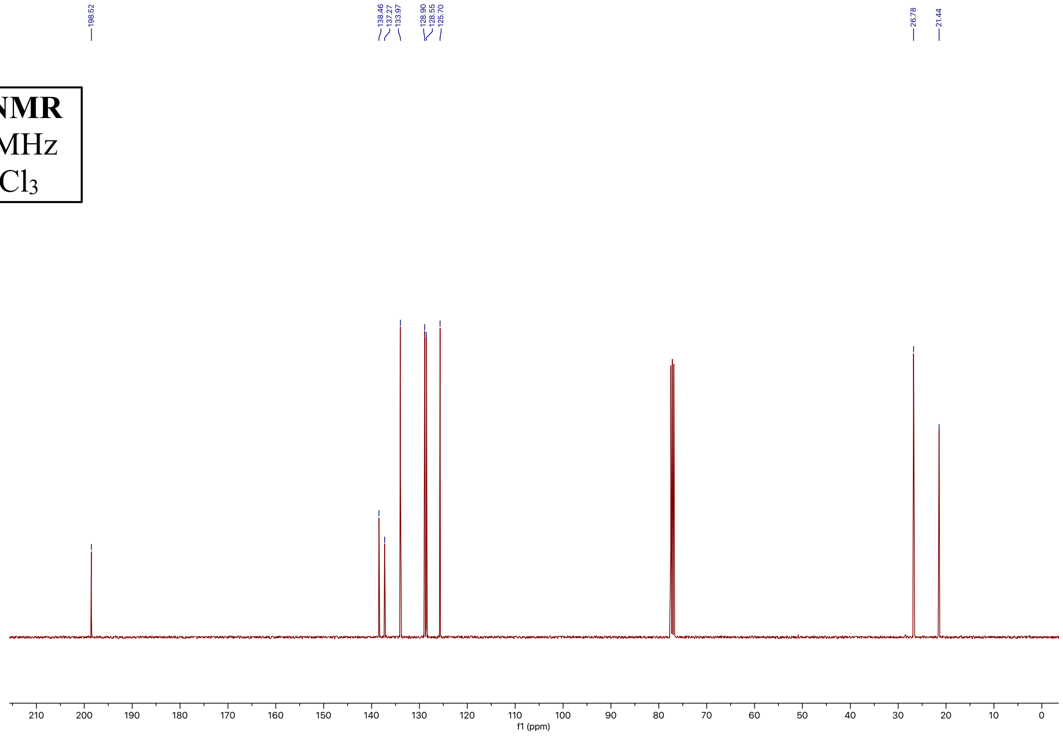

1-(*o*-Tolyl)ethan-1-one (14)

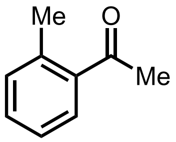

^{1}H-NMR
400 MHz
$CDCl_3$

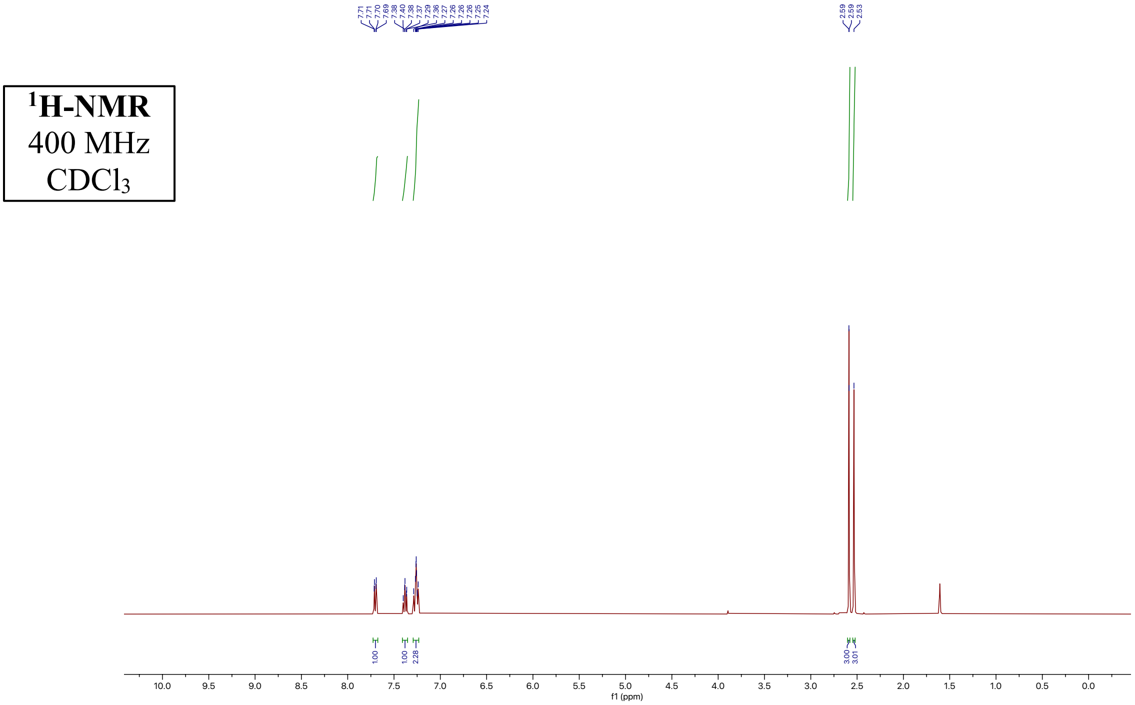

^{13}C-NMR
101 MHz
$CDCl_3$

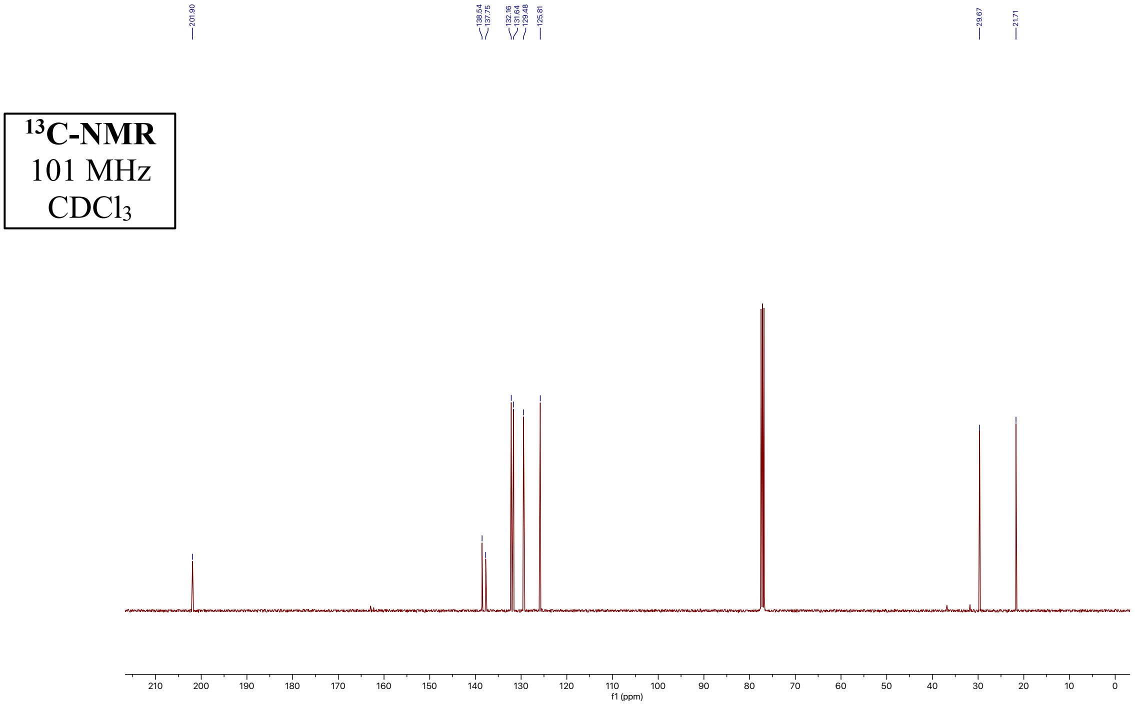

1-(2-Methoxyphenyl)ethan-1-one (15)

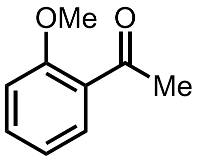

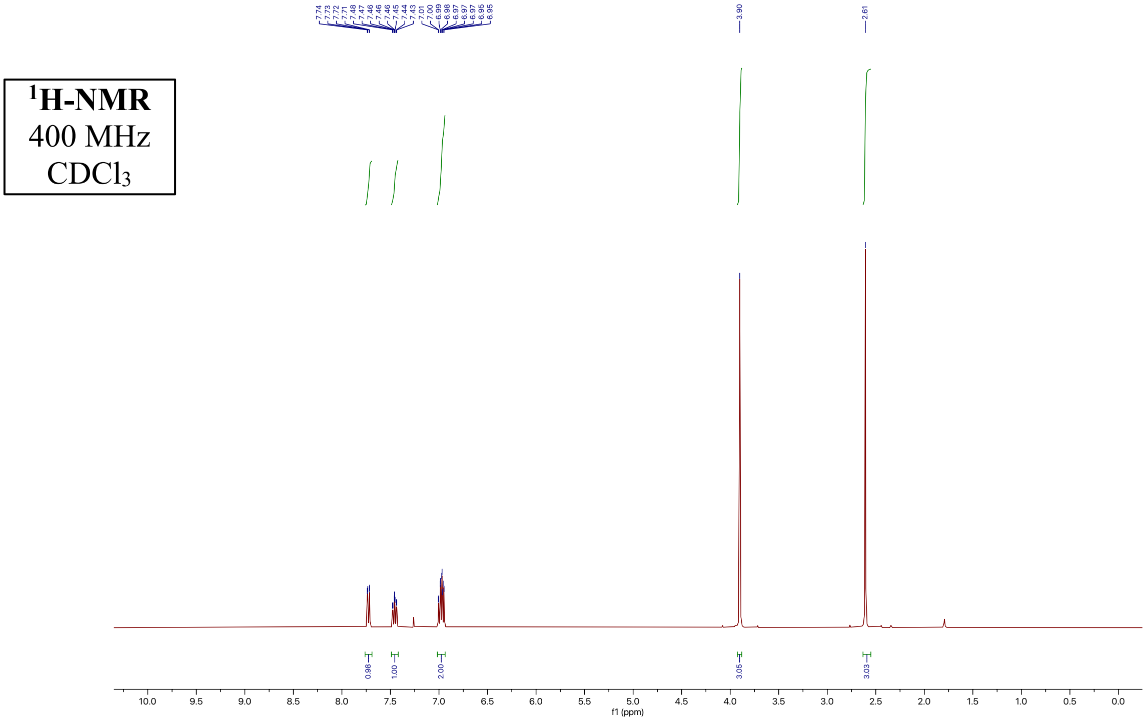

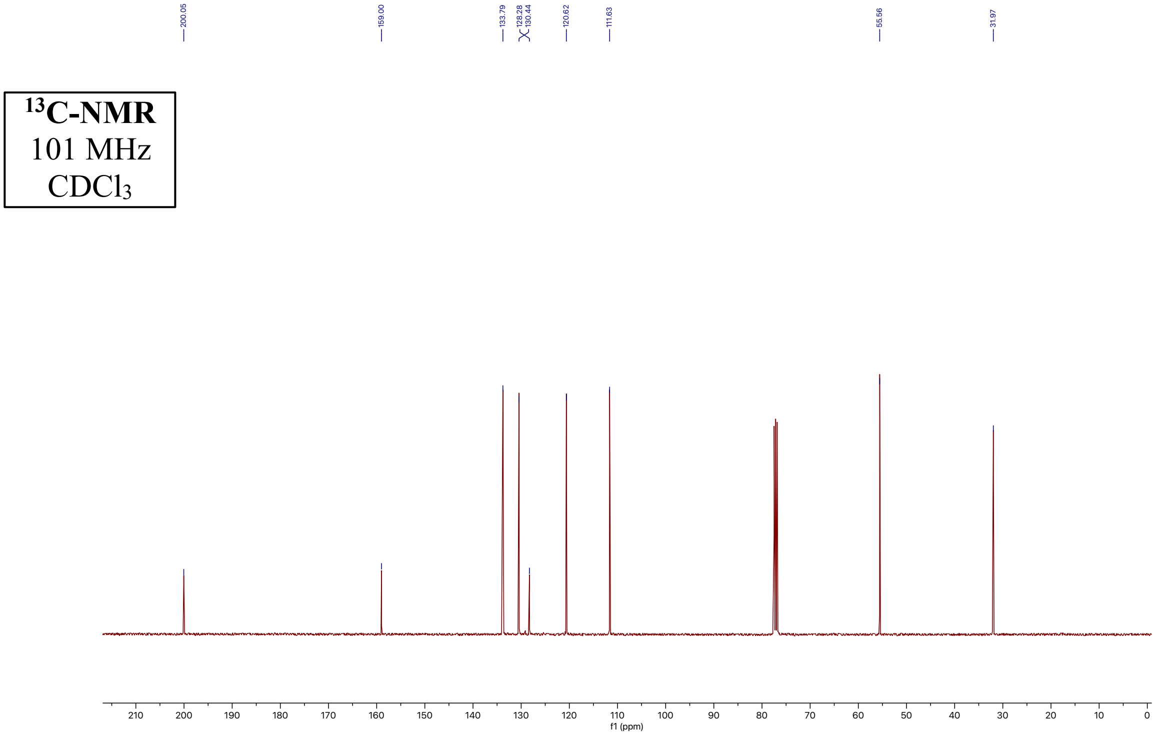

1-(Thiophen-2-yl)ethan-1-one (16)

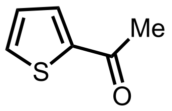

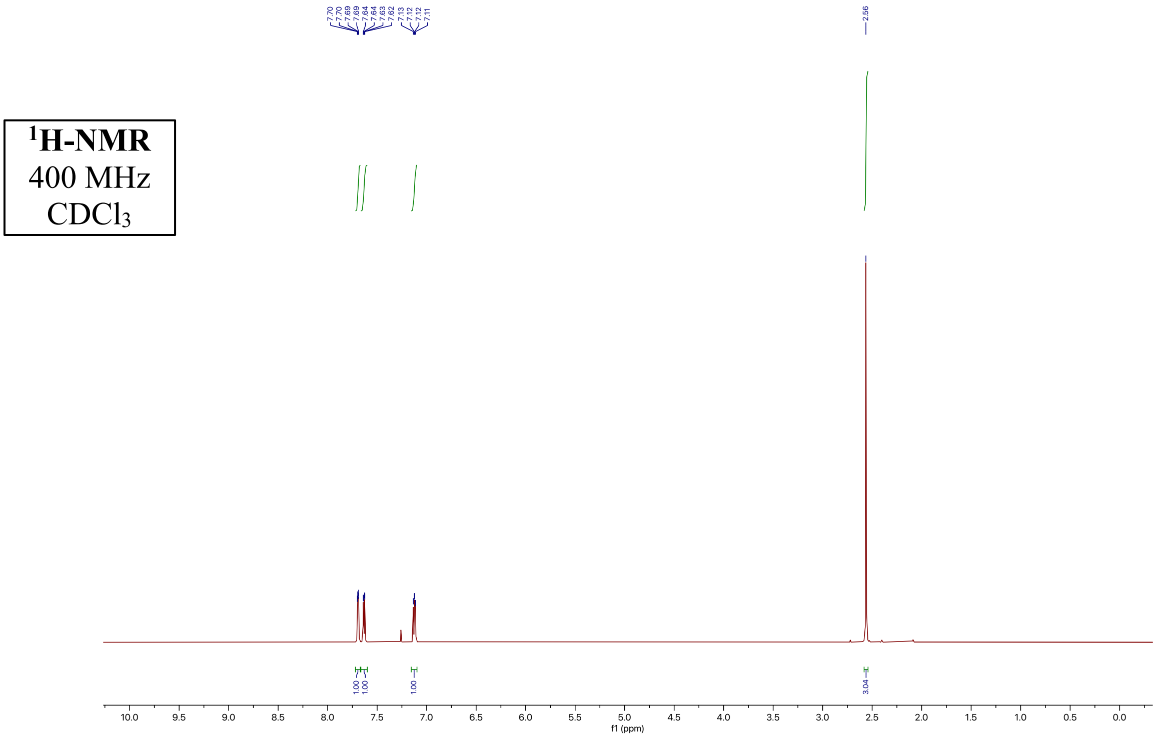

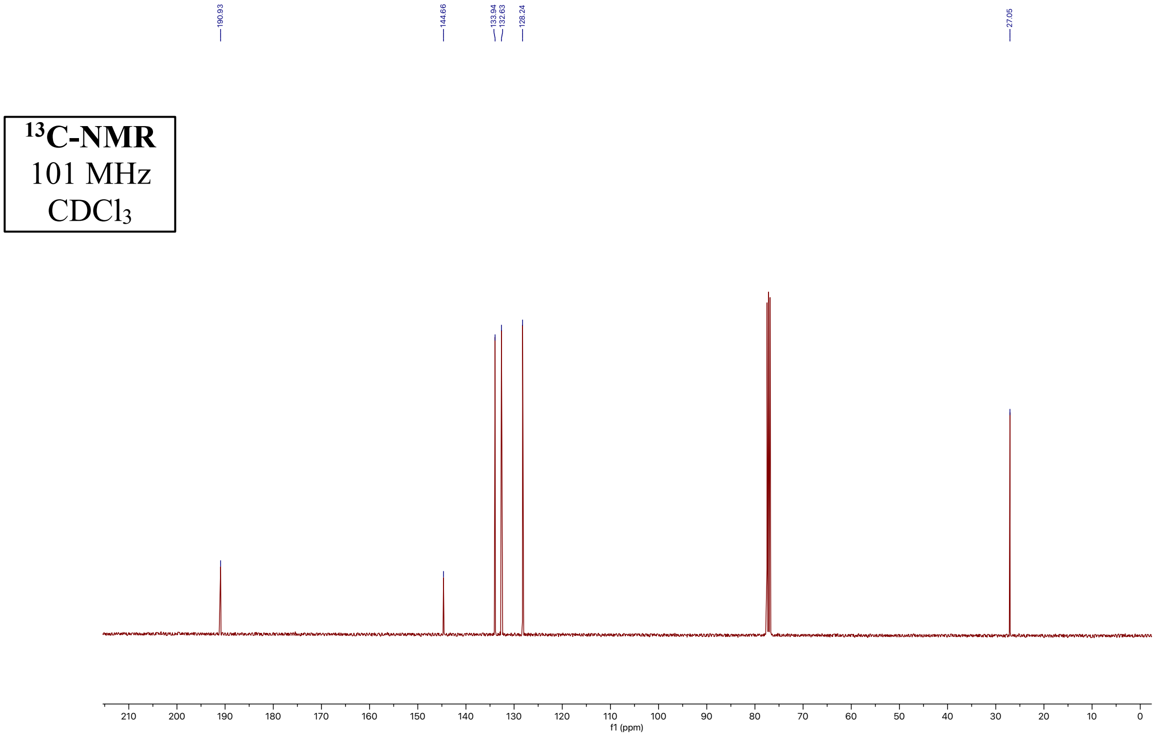

Phenyl(thiophen-2-yl)methanone (17)

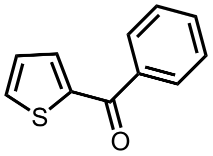

^{1}H-NMR
400 MHz
$CDCl_3$

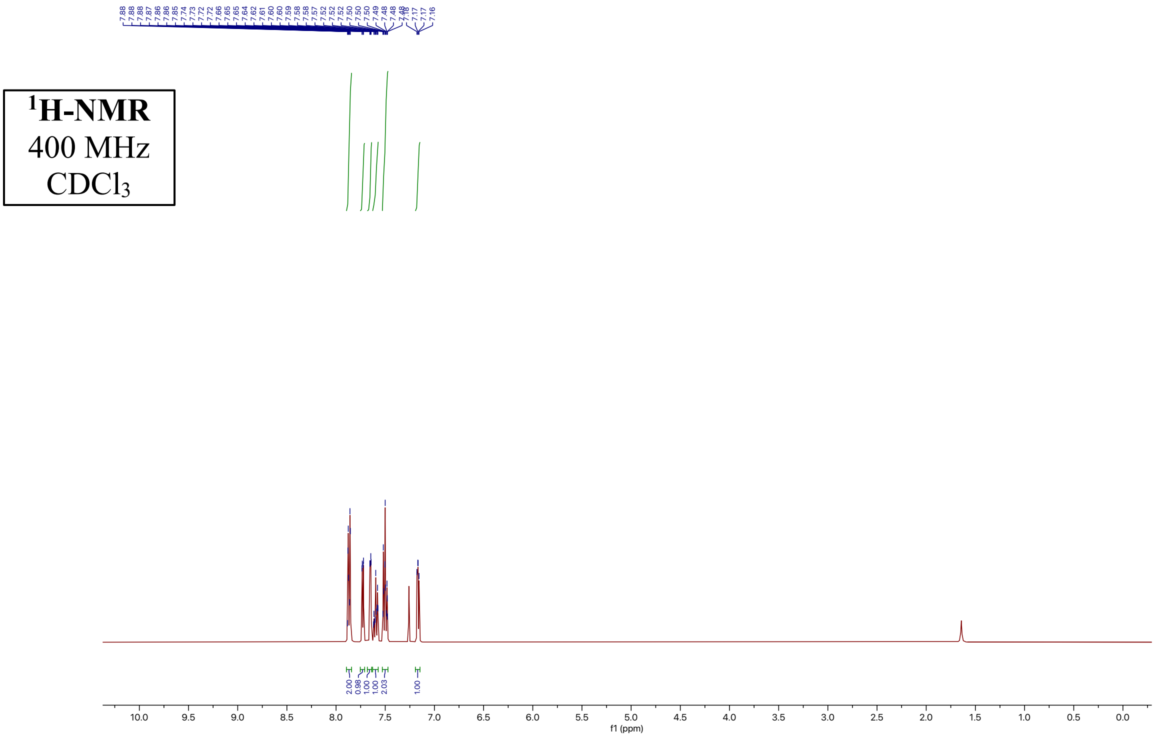

^{13}C-NMR
101 MHz
$CDCl_3$

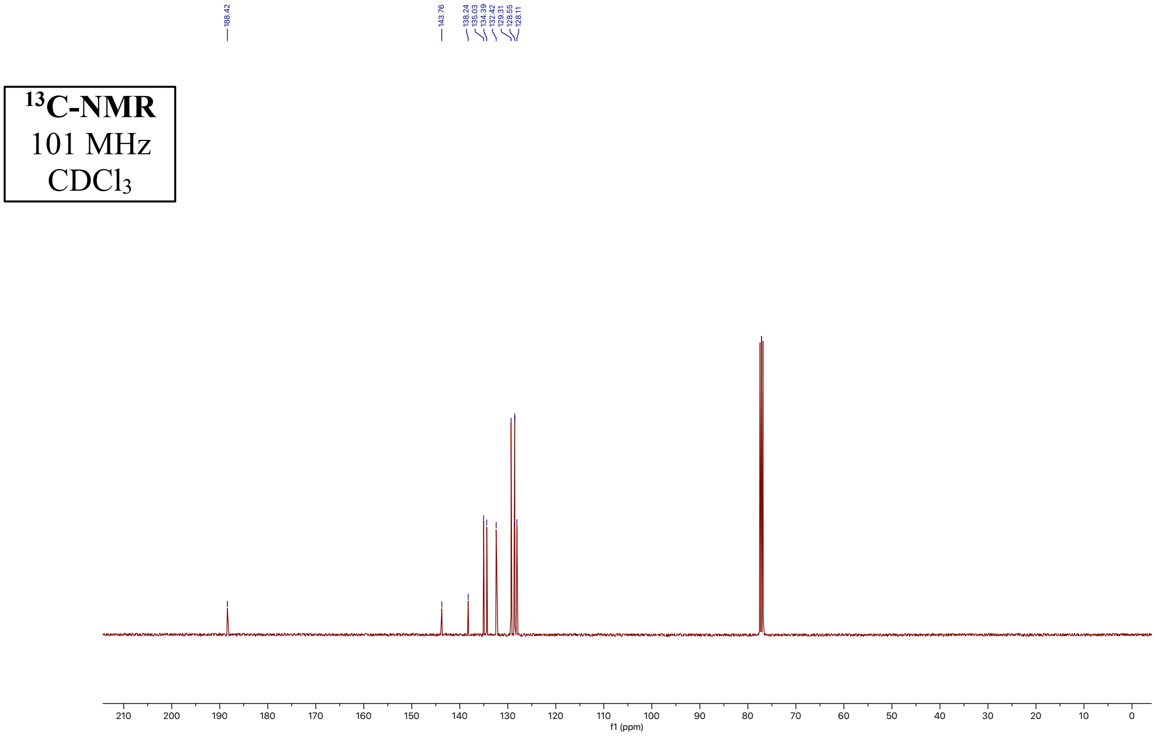

1-(Naphthalen-2-yl)ethan-1-one (18)

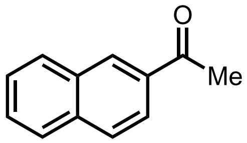

^{1}H-NMR
400 MHz
$CDCl_3$

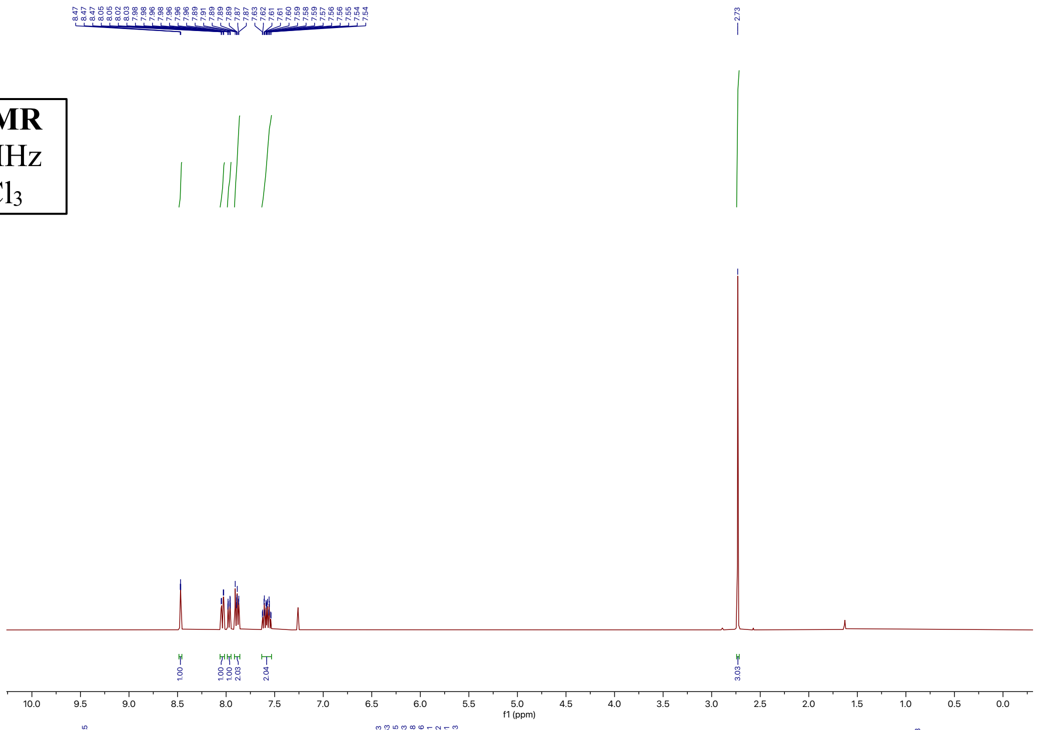

^{13}C-NMR
101 MHz
$CDCl_3$

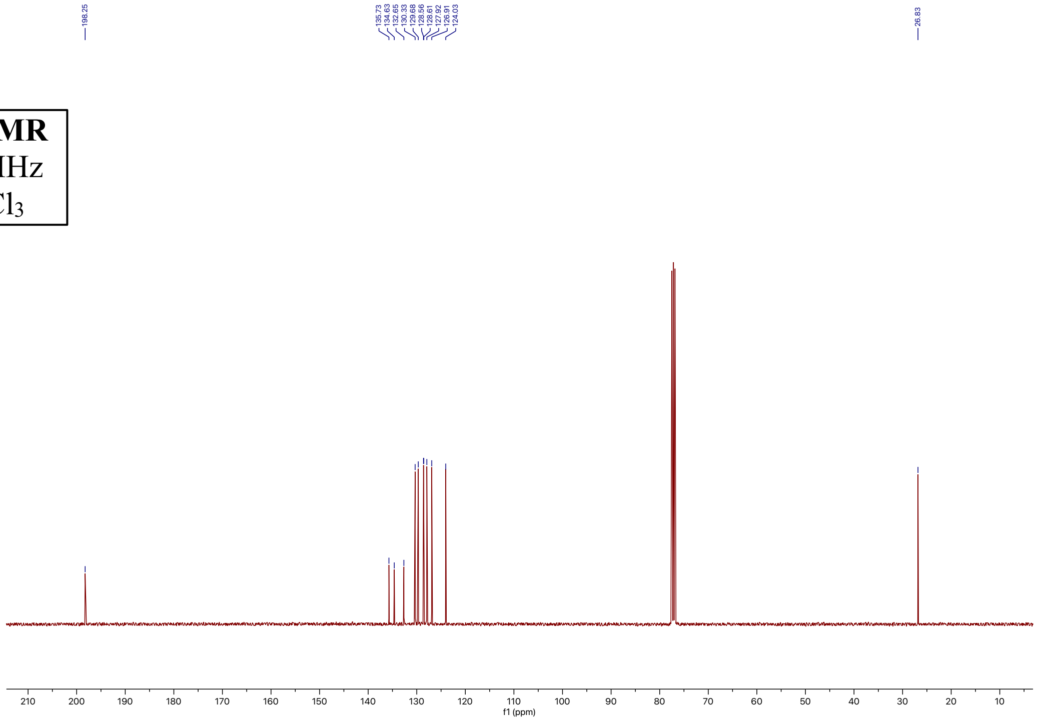

1-(2,3-Dihydrobenzofuran-5-yl)ethan-1-one (19)

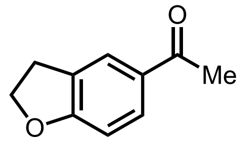

^{1}H-NMR
400 MHz
$CDCl_3$

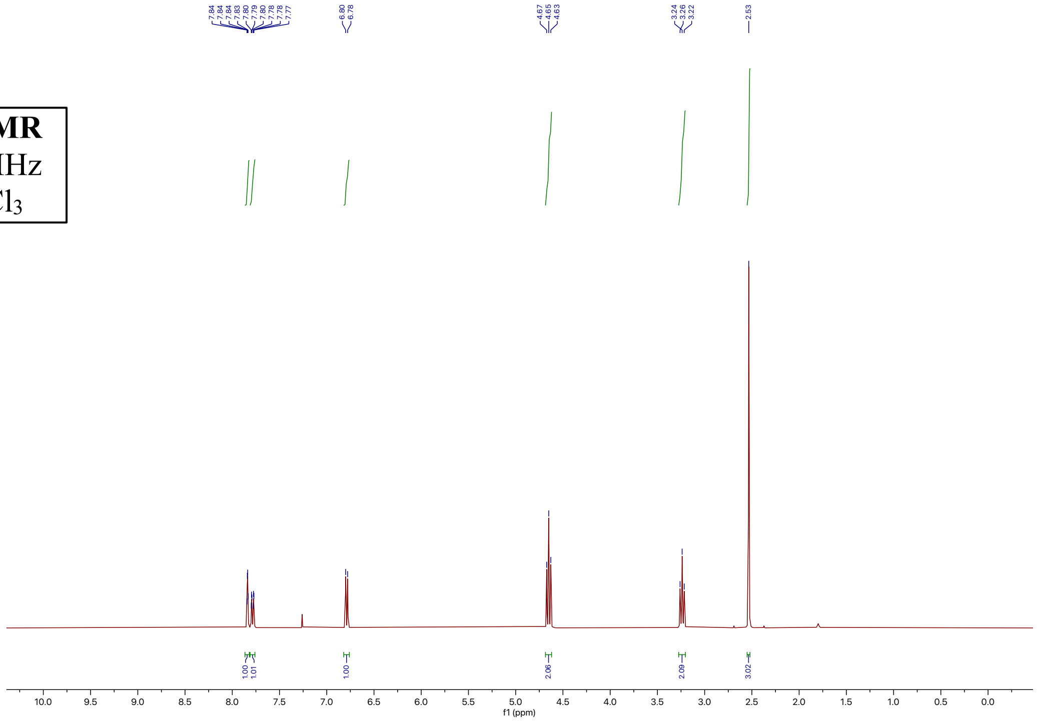

^{13}C-NMR
101 MHz
$CDCl_3$

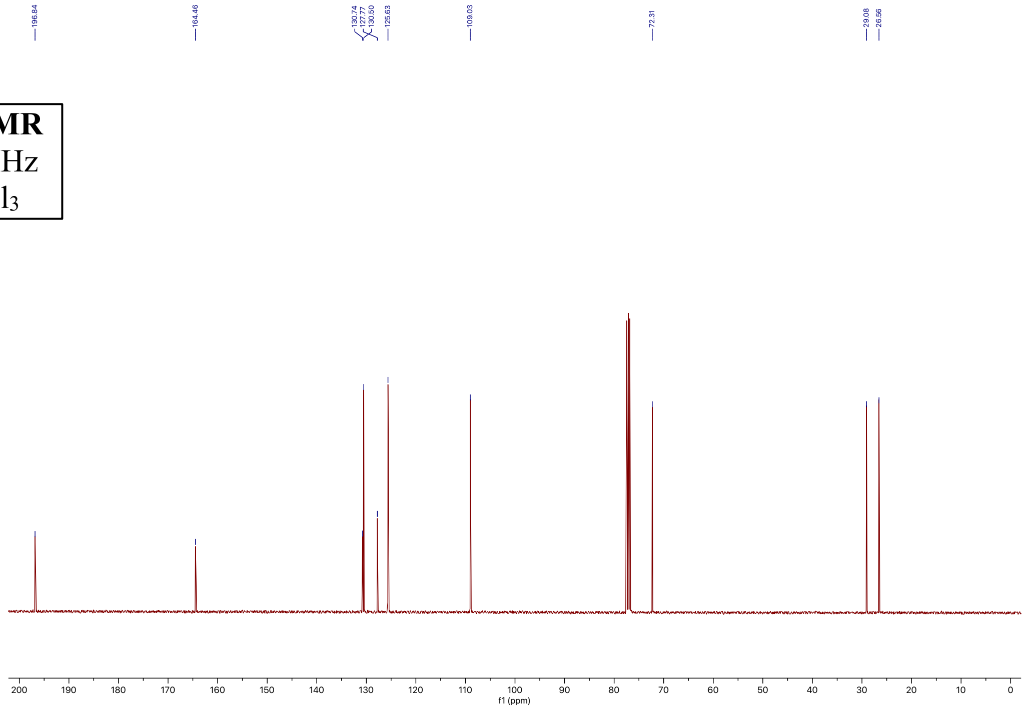

3,4-Dihydronaphthalen-1(2H)-one (20)

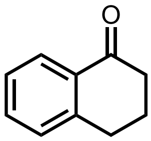

^{1}H-NMR
400 MHz
$CDCl_3$

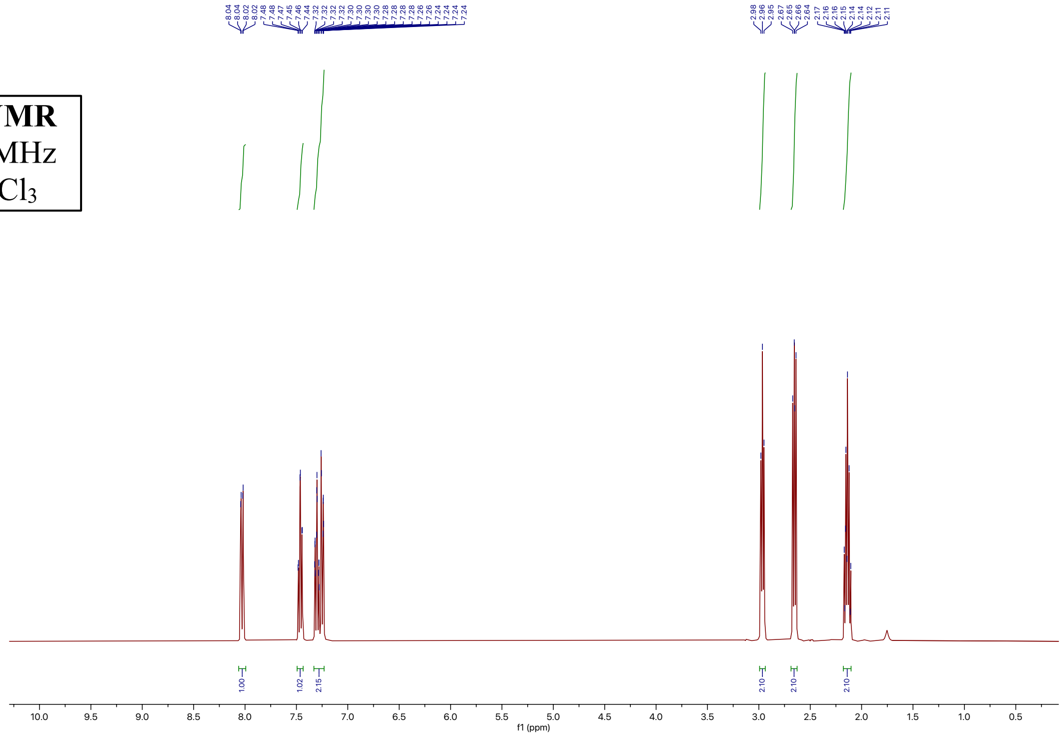

^{13}C-NMR
101 MHz
$CDCl_3$

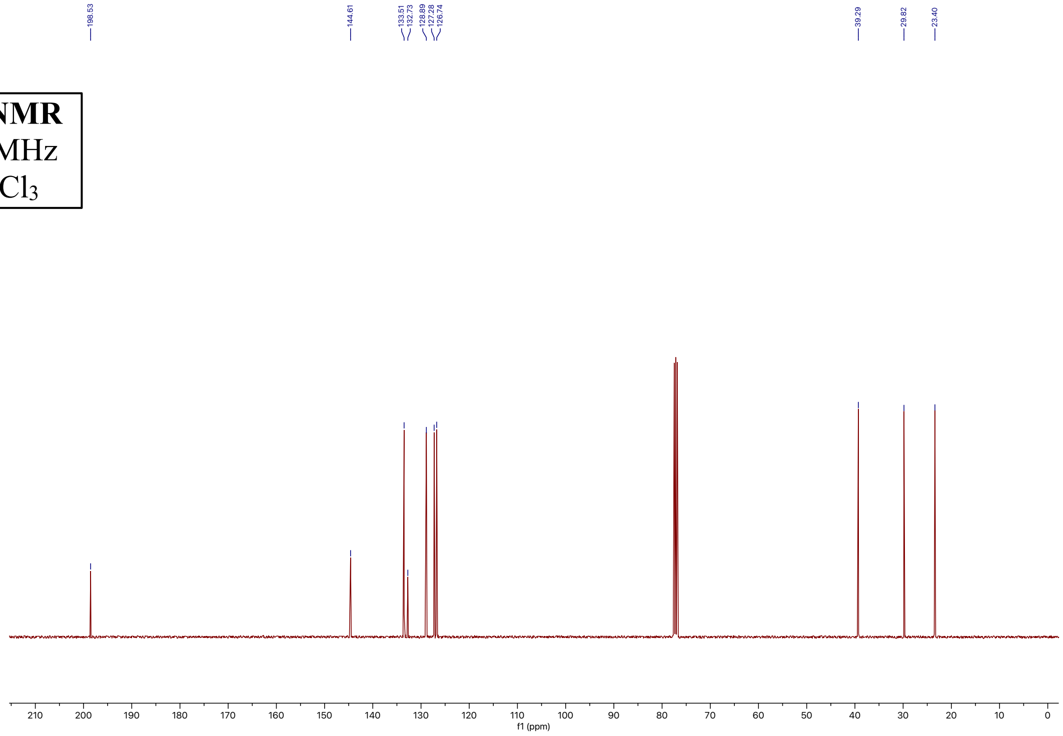

Propiophenone (21)

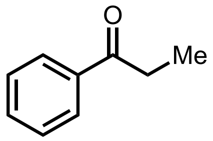

^{1}H-NMR
400 MHz
$CDCl_3$

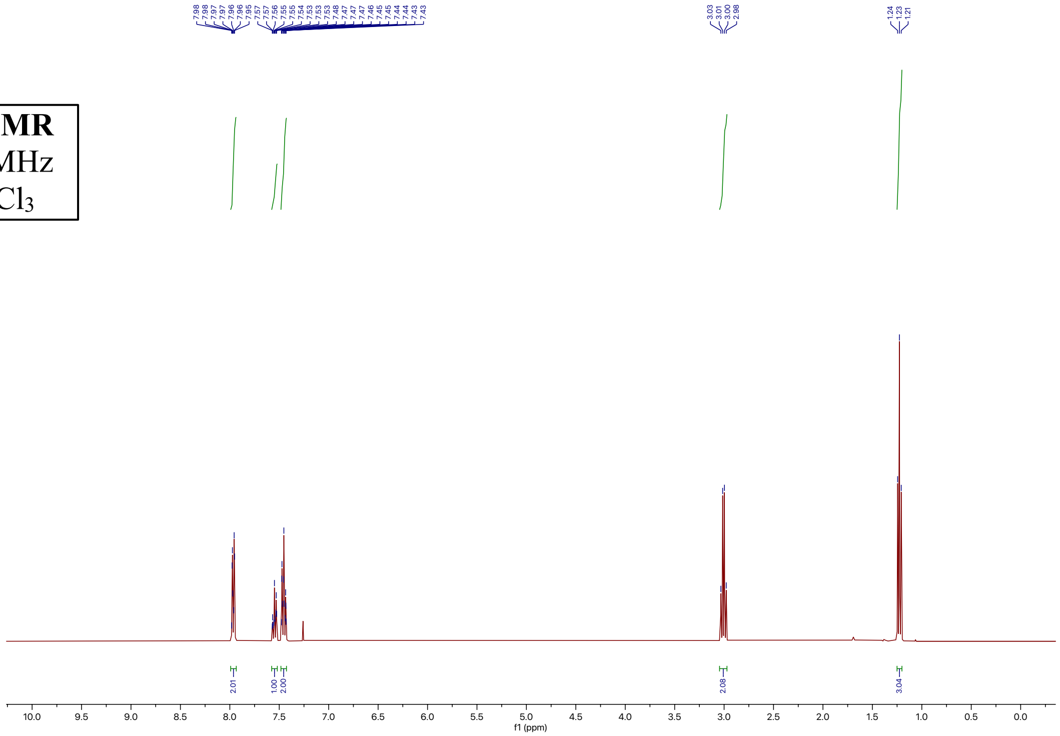

^{13}C-NMR
101 MHz
$CDCl_3$

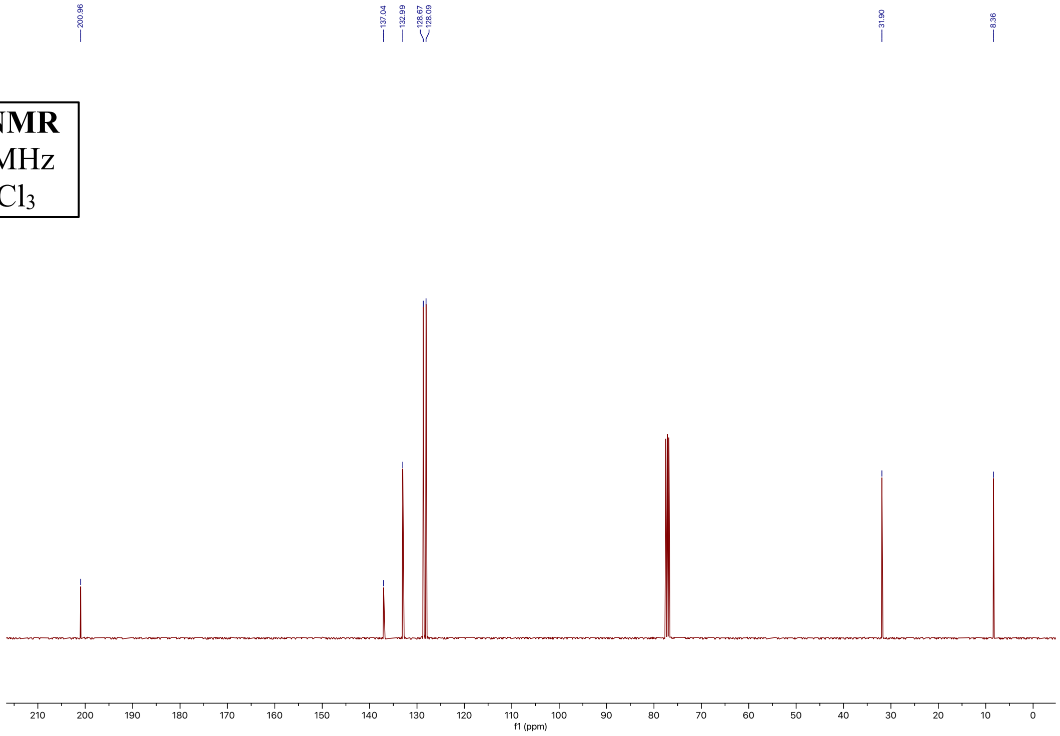

4-Phenylcyclohexan-1-one (22)

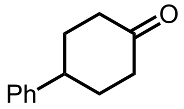

^{1}H-NMR
400 MHz
$CDCl_3$

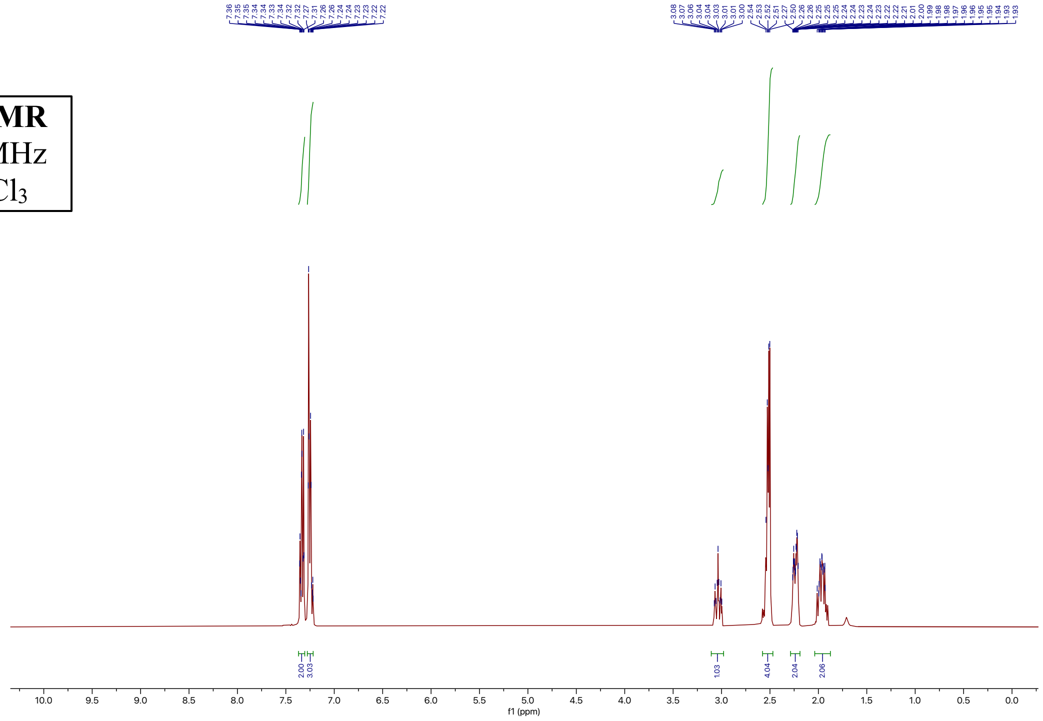

^{13}C-NMR
101 MHz
$CDCl_3$

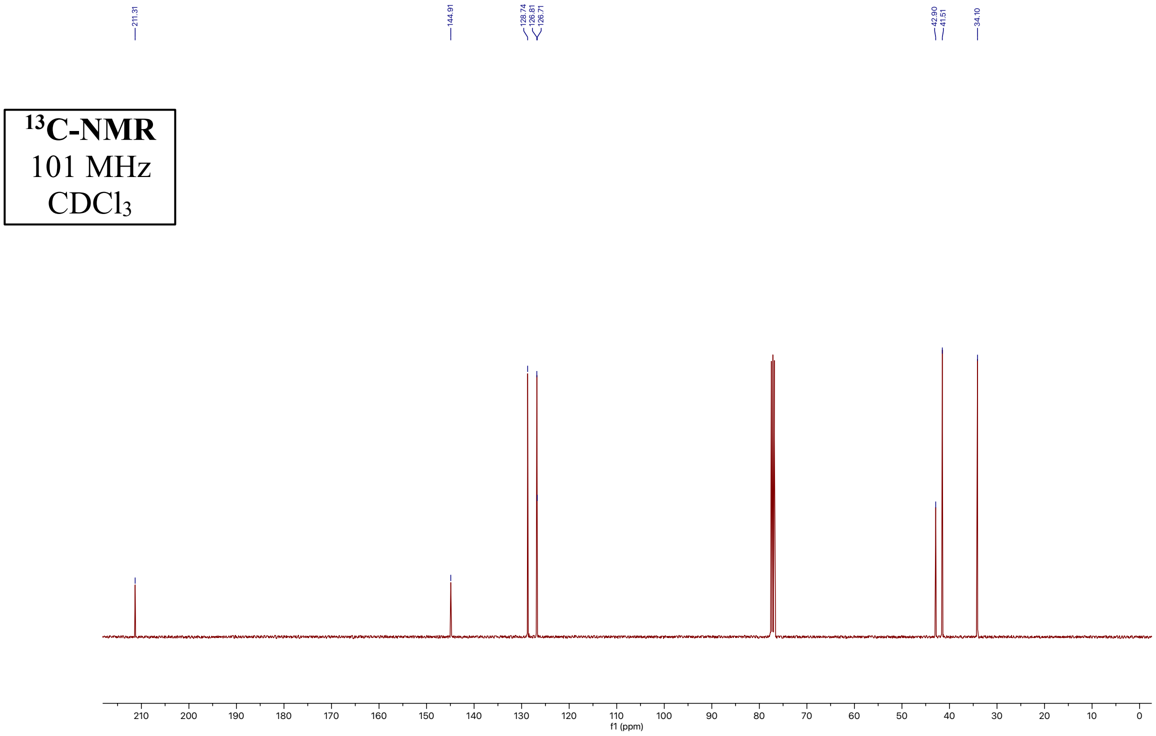

Benzaldehyde (23)

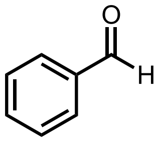

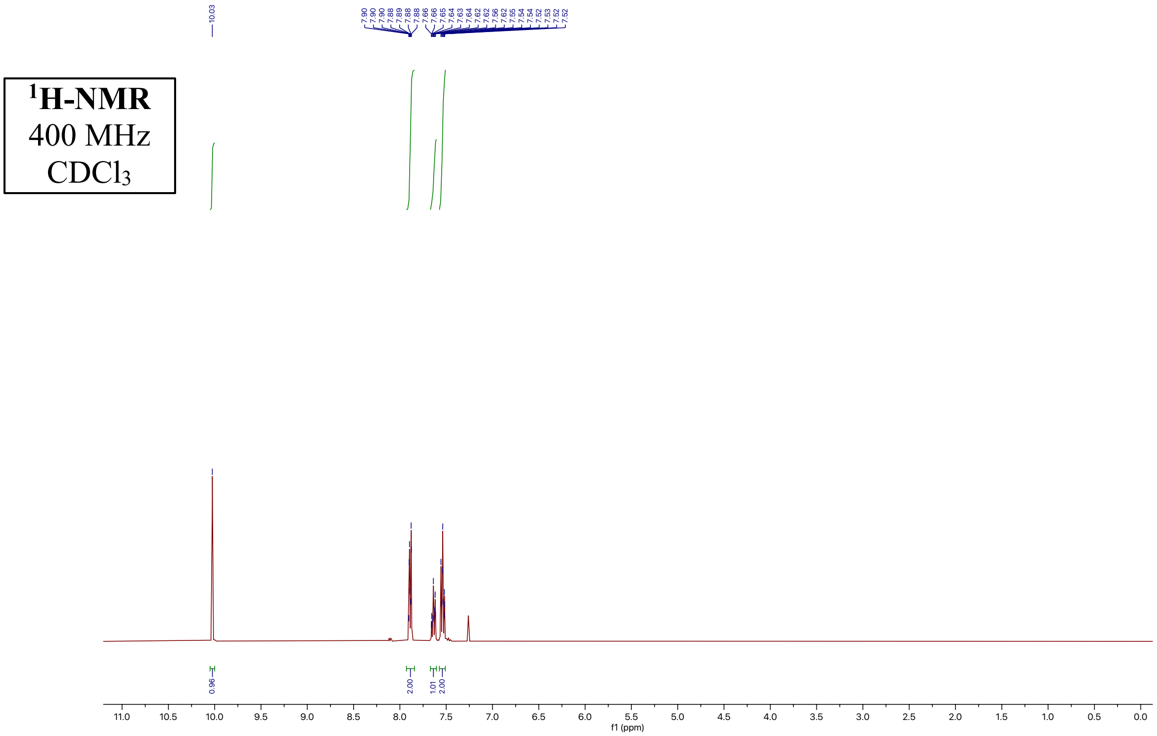

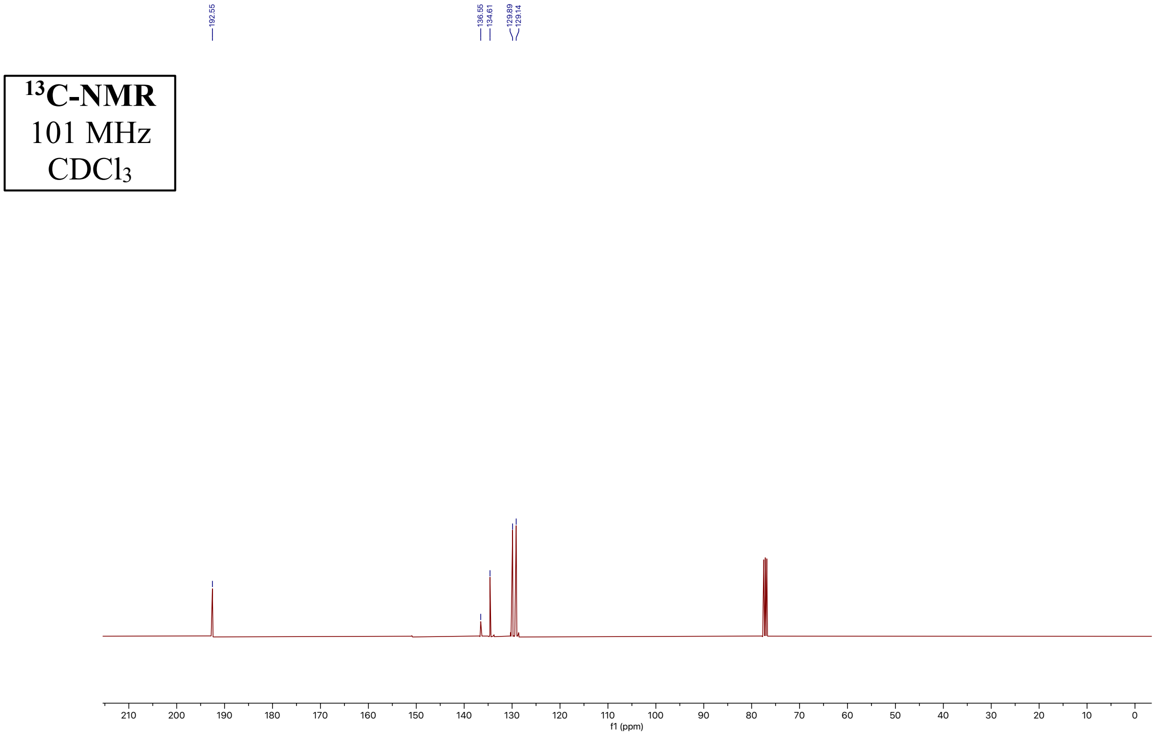

4-Hydroxy-3-methoxybenzaldehyde (24)

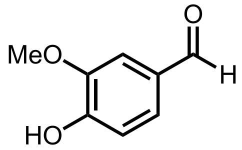

^{1}H-NMR
400 MHz
$CDCl_3$

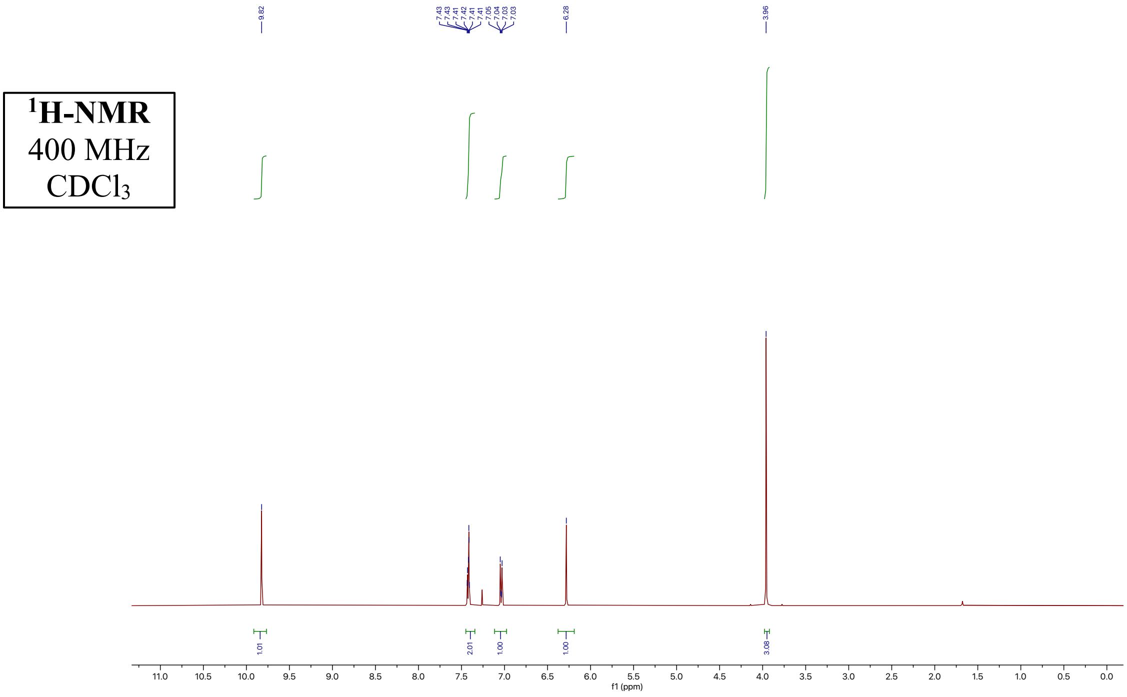

^{13}C-NMR
101 MHz
$CDCl_3$

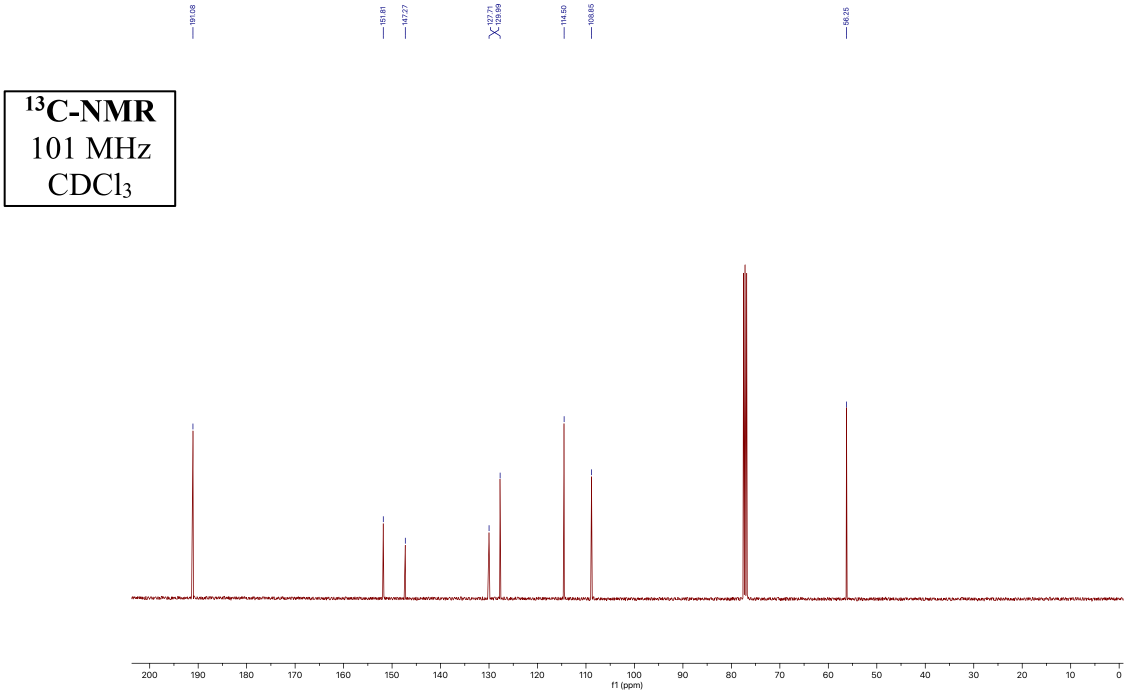

4-(Dimethylamino)benzaldehyde (25)

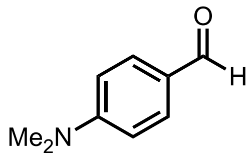

^{1}H-NMR
400 MHz
$CDCl_3$

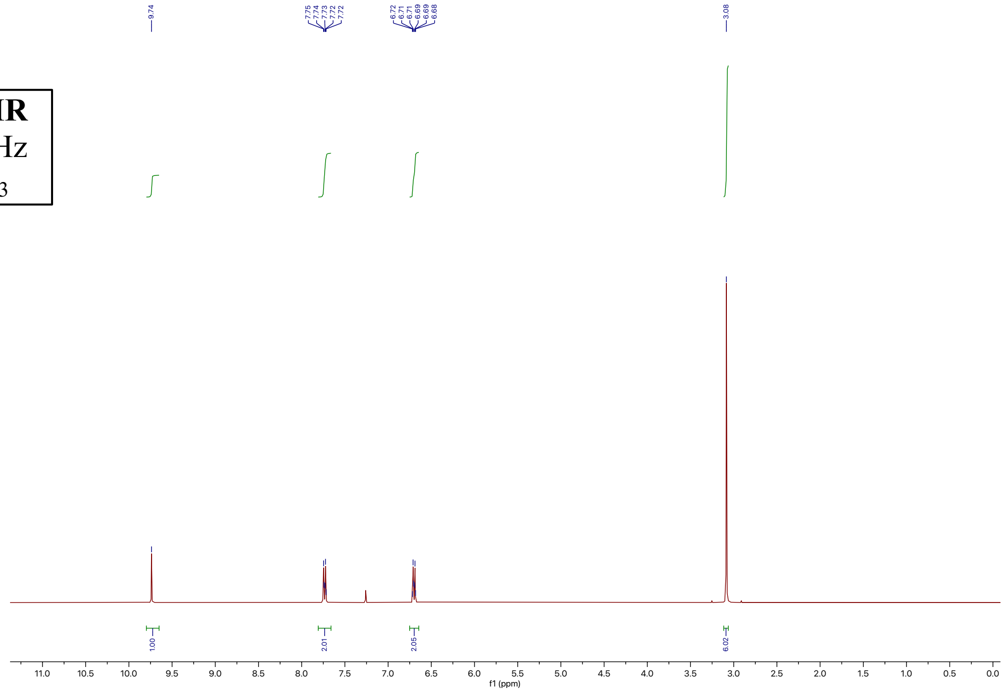

^{13}C-NMR
101 MHz
$CDCl_3$

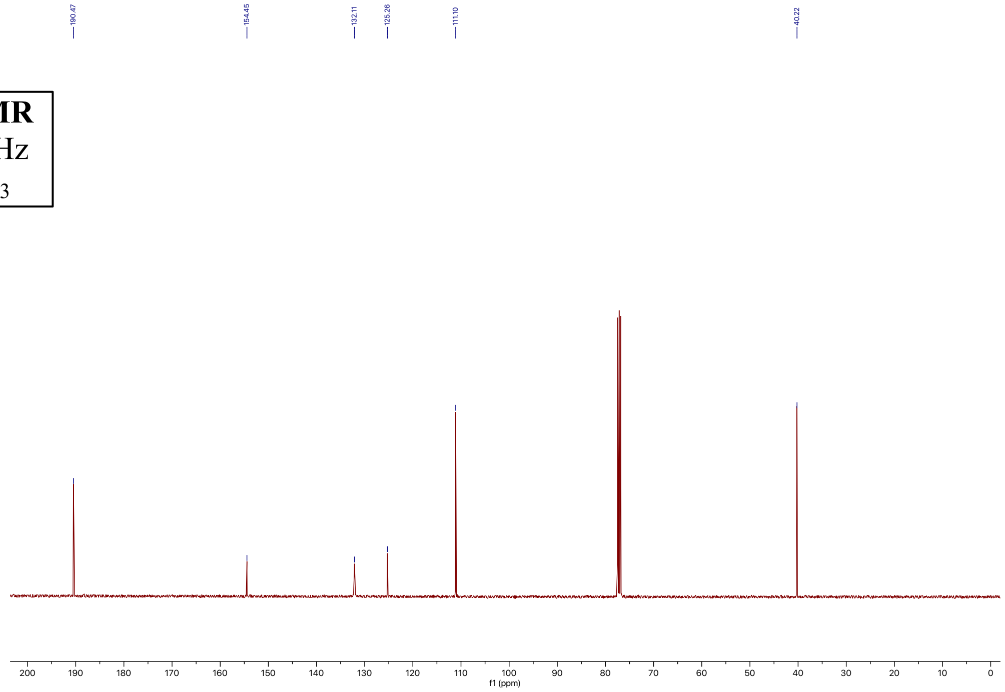

Nicotinaldehyde (26)

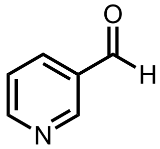

^{1}H-NMR
400 MHz
$CDCl_3$

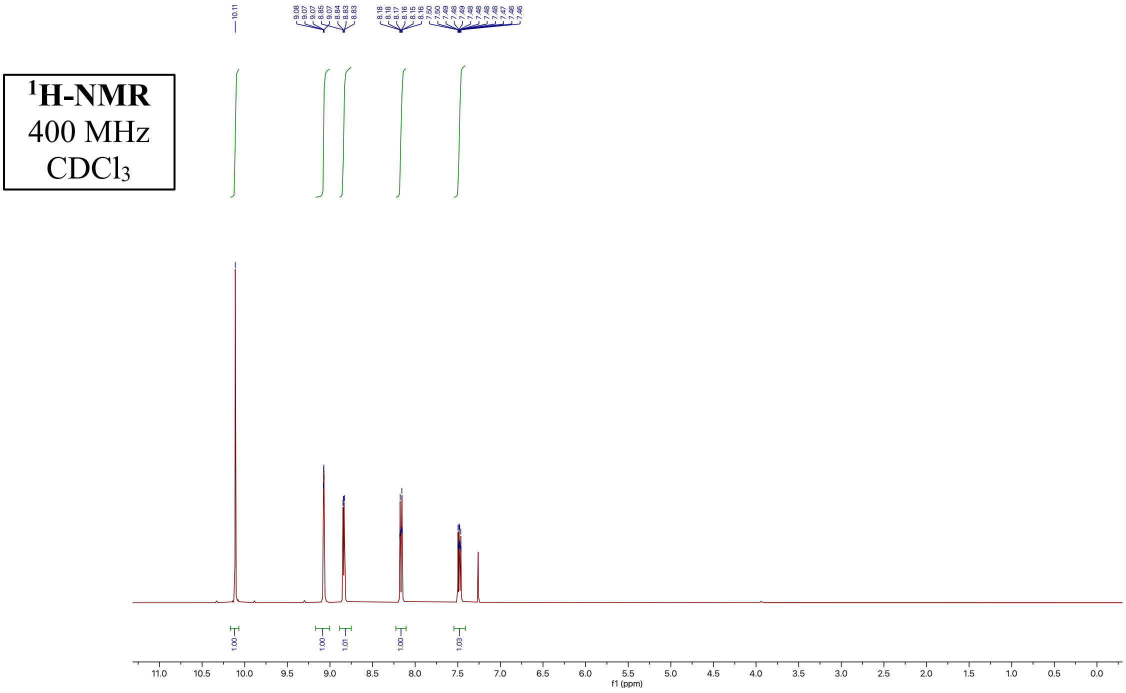

^{13}C-NMR
101 MHz
$CDCl_3$

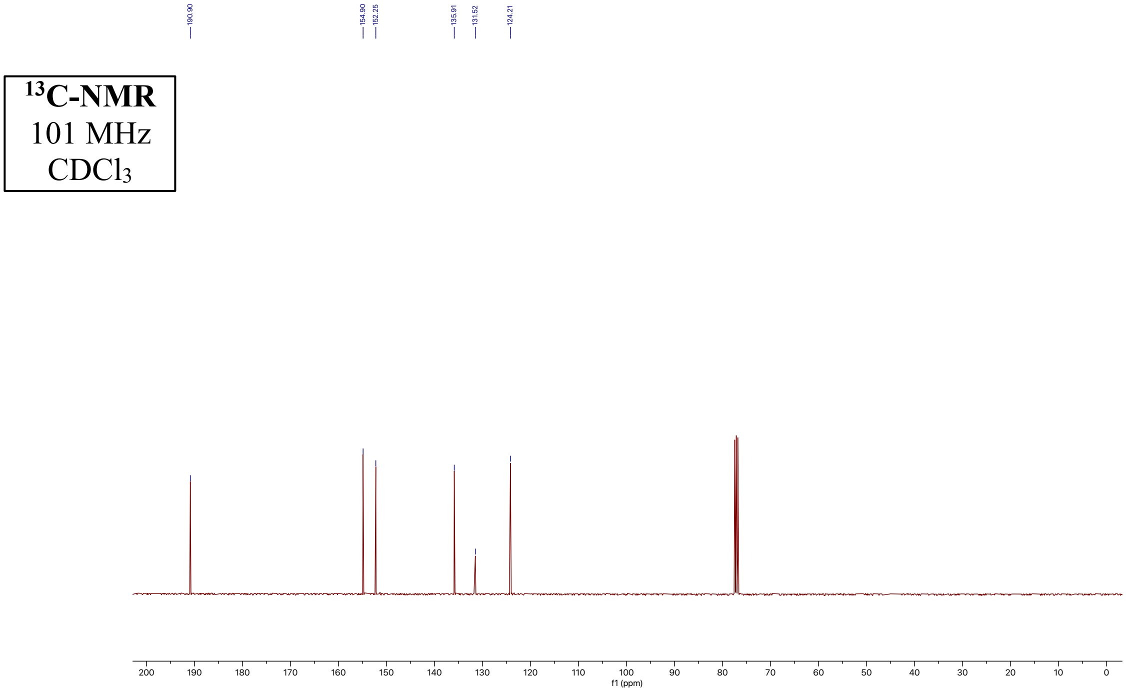

1H-indole-3-carbaldehyde (27)

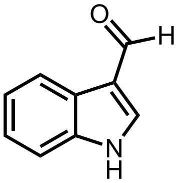

^{1}H-NMR
400 MHz
DMSO-d_6

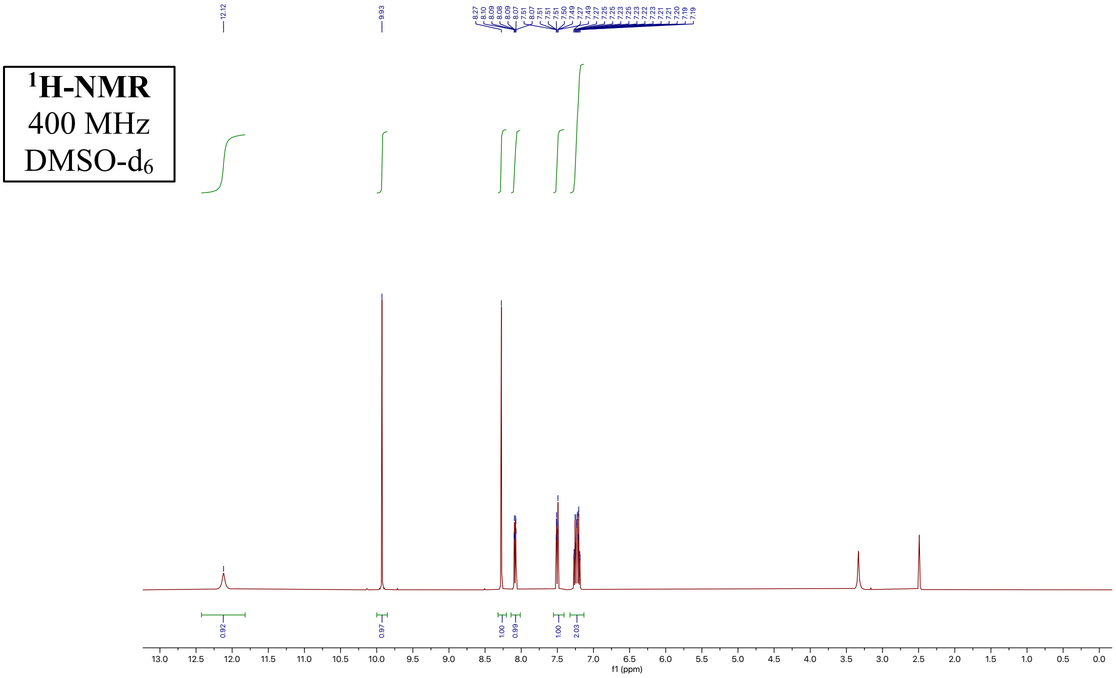

^{13}C-NMR
101 MHz
DMSO-d_6

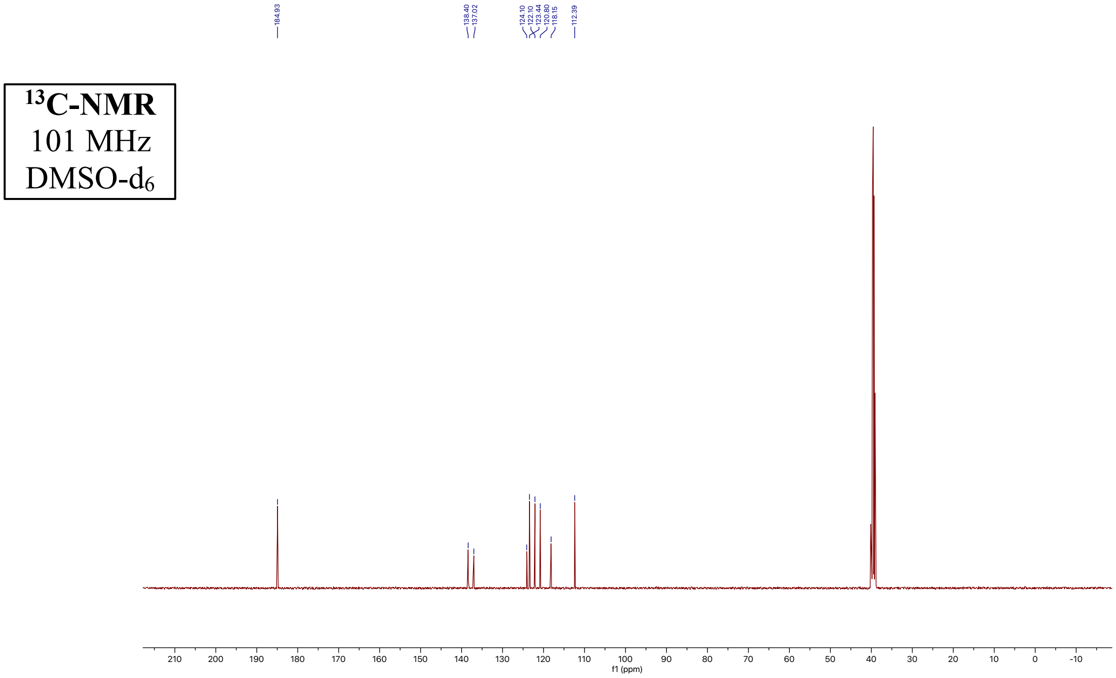

2-Methyl-2-phenyl-1,3-dioxolane (28)

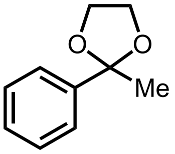

^{1}H-NMR
400 MHz
$CDCl_3$

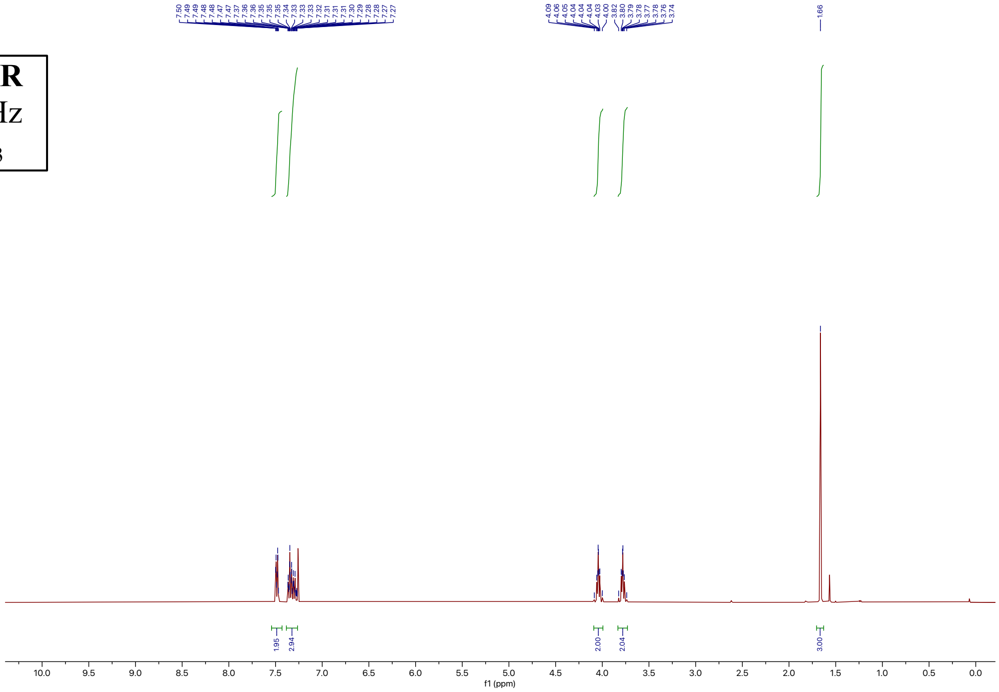

^{13}C-NMR
101 MHz
$CDCl_3$

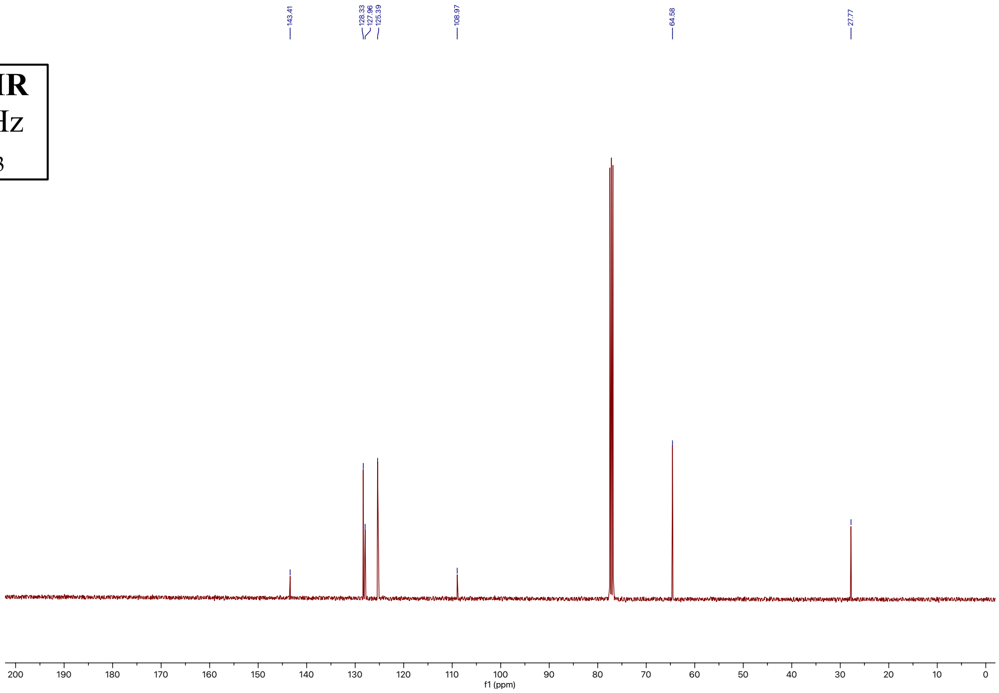

2-Methyl-2-phenyl-1,3-dithiane (29)

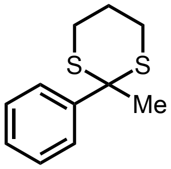

^{1}H-NMR
400 MHz
$CDCl_3$

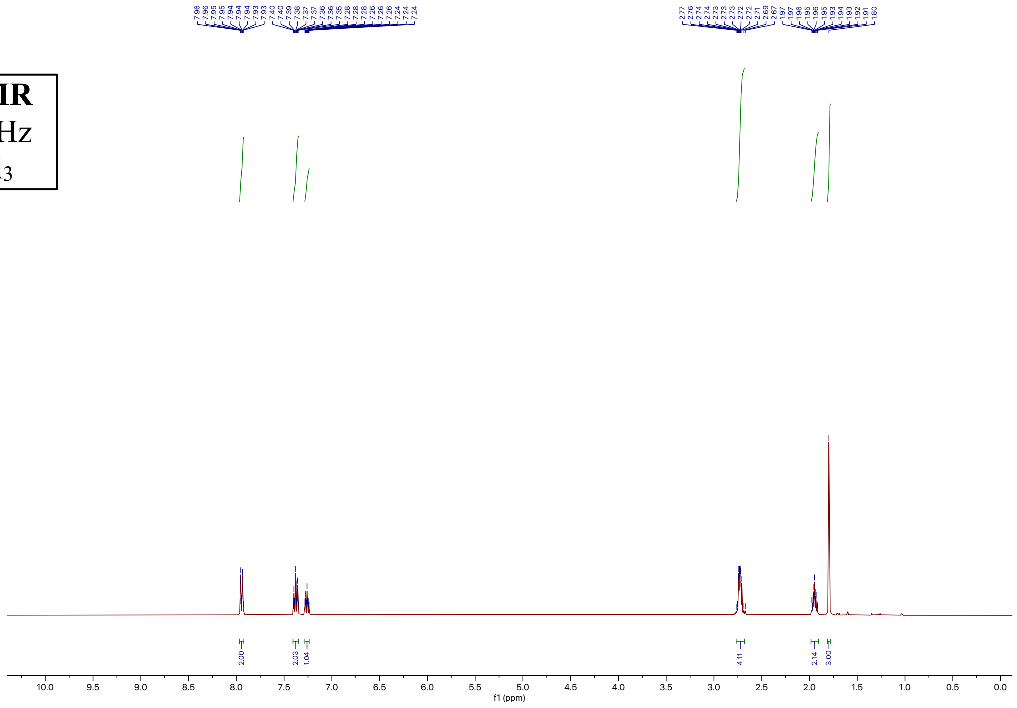

^{13}C-NMR
101 MHz
$CDCl_3$

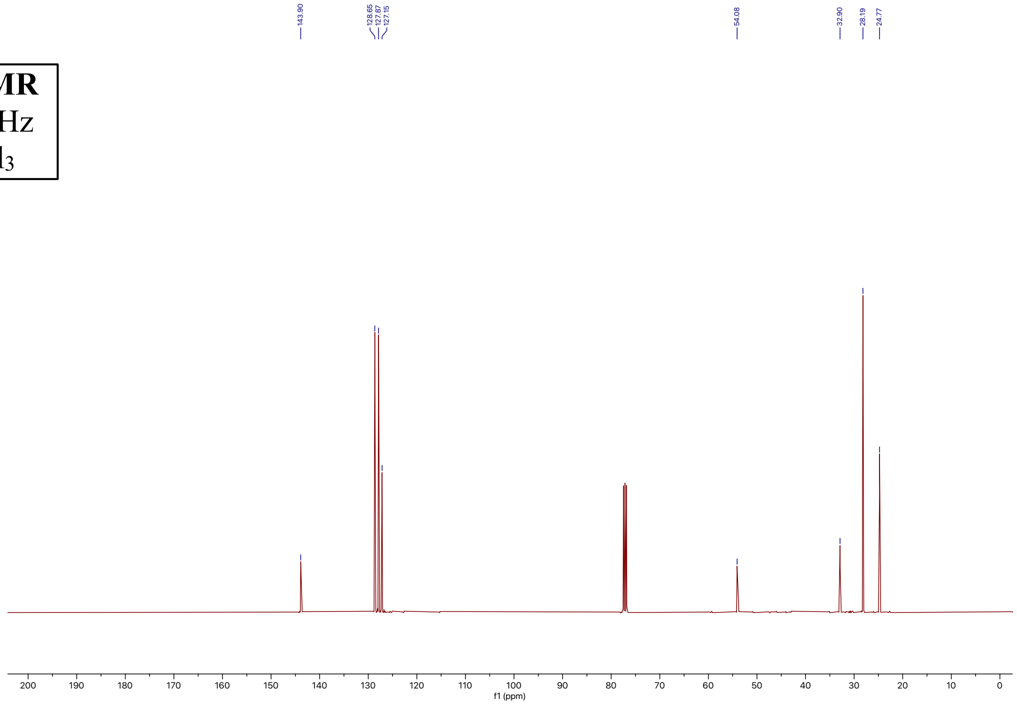

2-Methyl-2-phenyl-1,3-oxathiolane (30)

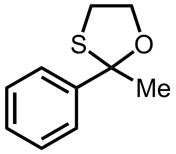

^{1}H-NMR
400 MHz
$CDCl_3$

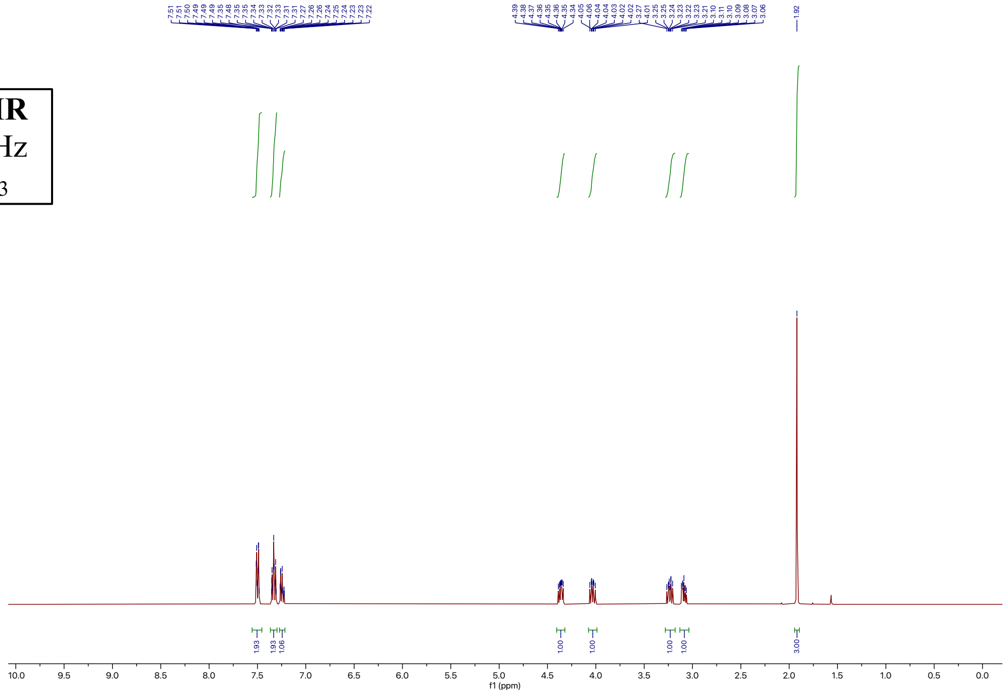

^{13}C-NMR
101 MHz
$CDCl_3$

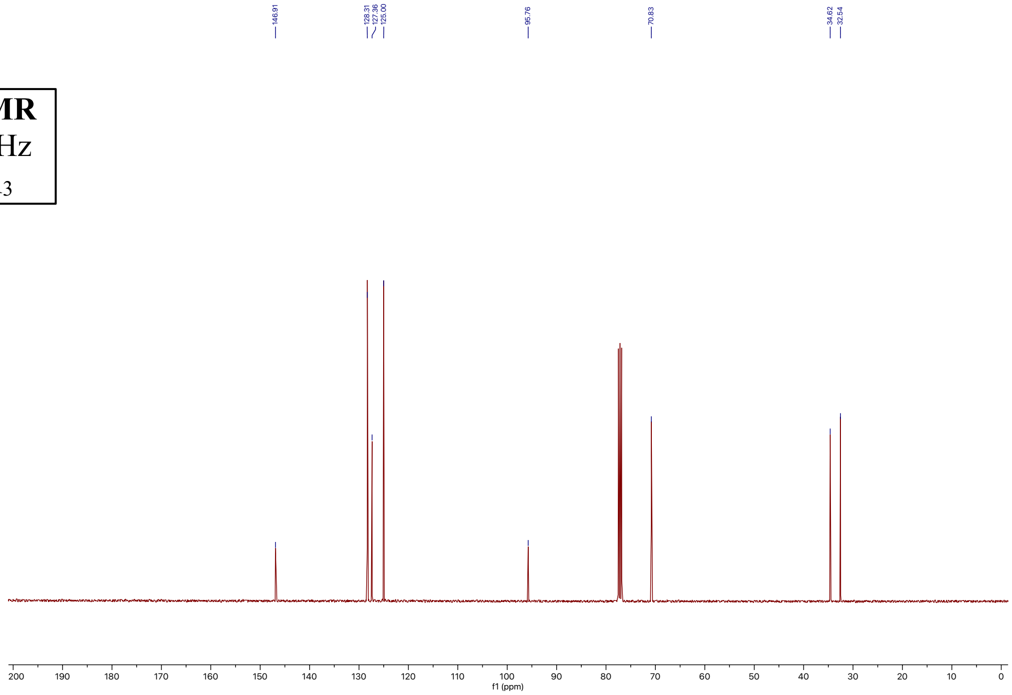

Supplement: Supplementary file 1 [file ol5c01137_si_001.pdf]
